# Supplementary material for: Activation of the Notch Signaling Pathway In Vivo Elicits Changes in CSL Nuclear Dynamics
Source: Dev Cell. 2018 Mar 12;44(5):611–623.e7. doi: 10.1016/j.devcel.2018.01.020 (PMC5855320; doi:10.1016/j.devcel.2018.01.020)
Supplement: Document S1. Figures S1–S7 and Tables S1–S5 [file mmc1.pdf]

Developmental Cell, Volume *44*

Supplemental Information

Activation of the Notch Signaling Pathway *In Vivo* Elicits Changes in CSL Nuclear Dynamics

Maria J. Gomez-Lamarca, Julia Falo-Sanjuan, Robert Stojnic, Sohaib Abdul Rehman, Leila Muresan, Matthew L. Jones, Zoe Pillidge, Gustavo Cerda-Moya, Zhenyu Yuan, Sarah Baloul, Phillippe Valenti, Kerstin Bystricky, Francois Payre, Kevin O'Holleran, Rhett Kovall, and Sarah J. Bray

Supplementary material

This PDF file includes:

Supplementary Figures S1 to S7 and legends
Tables S1 to S5 and legends

Other Supplementary Materials for this manuscript includes the following:

Movies S1 to S2

Figure S1

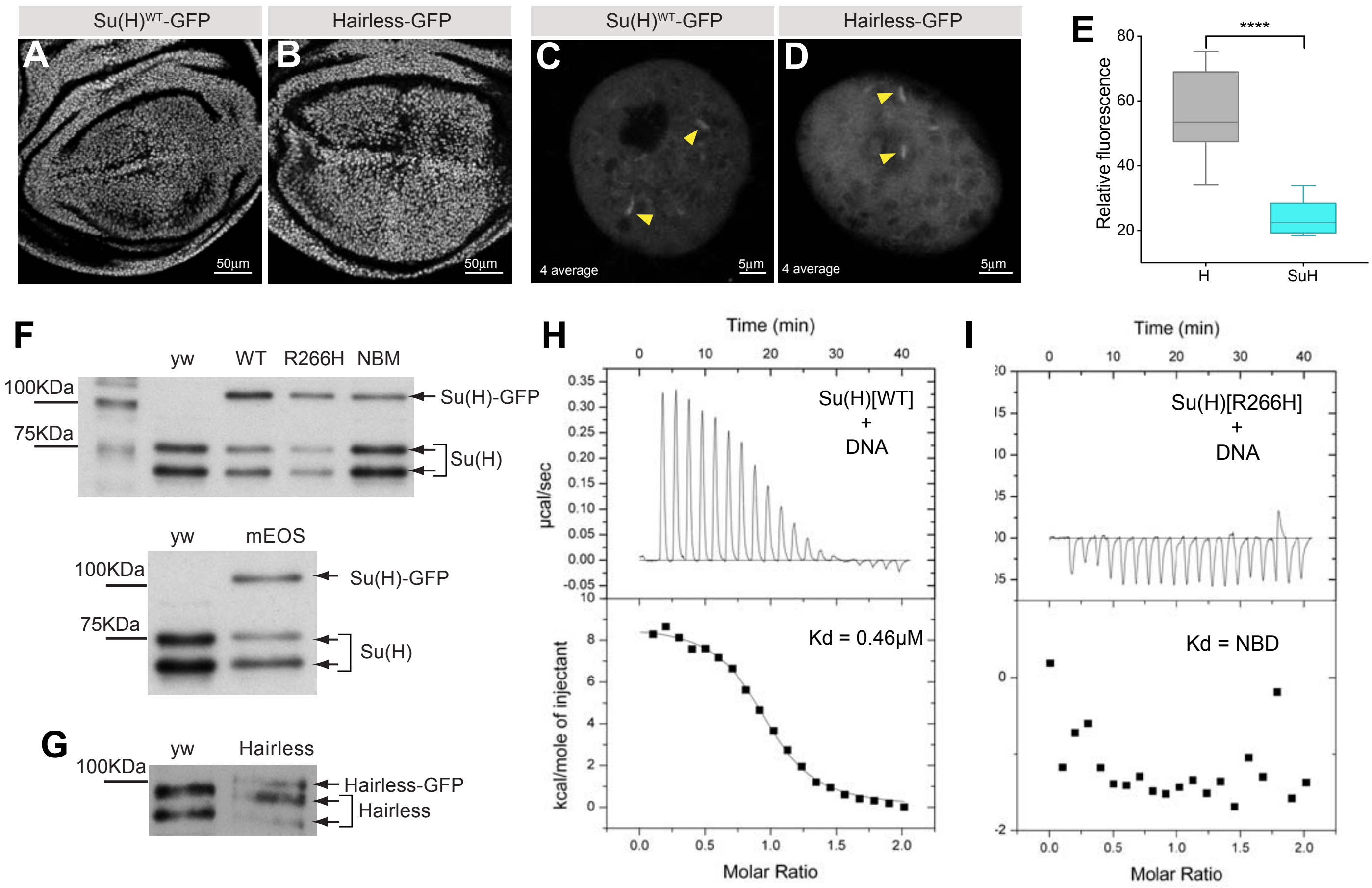

Su(H)WT-GFP
Hairless-GFP
Su(H)WT-GFP
Hairless-GFP
A
B
C
D
E
50µm
50µm
4 average
5µm
4 average
5µm
Relative fluorescence
****
H
SuH
F
yw
WT
R266H
NBM
100KDa
75KDa
Su(H)-GFP
Su(H)
yw
mEOS
100KDa
75KDa
Su(H)-GFP
Su(H)
G
yw
Hairless
100KDa
Hairless-GFP
Hairless
H
Time (min)
µcal/sec
Su(H)[WT]
+
DNA
kcal/mole of injectant
Kd = 0.46µM
Molar Ratio
I
Time (min)
Su(H)[R266H]
+
DNA
Kd = NBD
Molar Ratio

Figure. S1; Related to Figure 1. Characteristics of Su(H)^{WT}::GFP, Hairless::GFP, Su(H)^{R266H}::GFP

(**A-B**) Wing imaginal disc expressing Su(H)^{WT} (**A**) or Hairless::GFP (**B**). (**C-D**) Salivary gland cells expressing Su(H)^{WT} (**C**) or Hairless::GFP (**D**), imaged with 4 frame averages, note binding at several loci in the polytene chromosomes (arrowheads). (**E**) Fluorescence levels of Su(H)^{WT}::GFP and Hairless::GFP in salivary gland nuclei (n=9, Box and whiskers, Min-to-Max, ****$p < 0.0001$, unpaired two-tailed t test). (**F,G**) Fusion proteins are expressed at similar levels to endogenous. (**F**) Levels of Su(H) detected by western blot in controls (yw) or flies expressing Su(H)^{WT}::GFP, Su(H)^{R266H}::GFP, Su(H)^{NBM}::GFP, Su(H)::mEOS; bands corresponding to endogenous Su(H) or Su(H)::GFP are indicated by arrows. Extracts were prepared from salivary glands of larvae homozygous for the transgene except for Su(H)^{NBM}::GFP which were prepared from heterozygous larva, with only 1 copy of the transgene. (**G**) Levels of Hairless detected in controls (yw) or flies expressing Hairless::GFP, as in F. (**H-I**) Isothermal calorimetry measurements for Su(H)^{WT} (**H**) or Su(H)^{R266H} (**I**) with DNA.

Figure S2

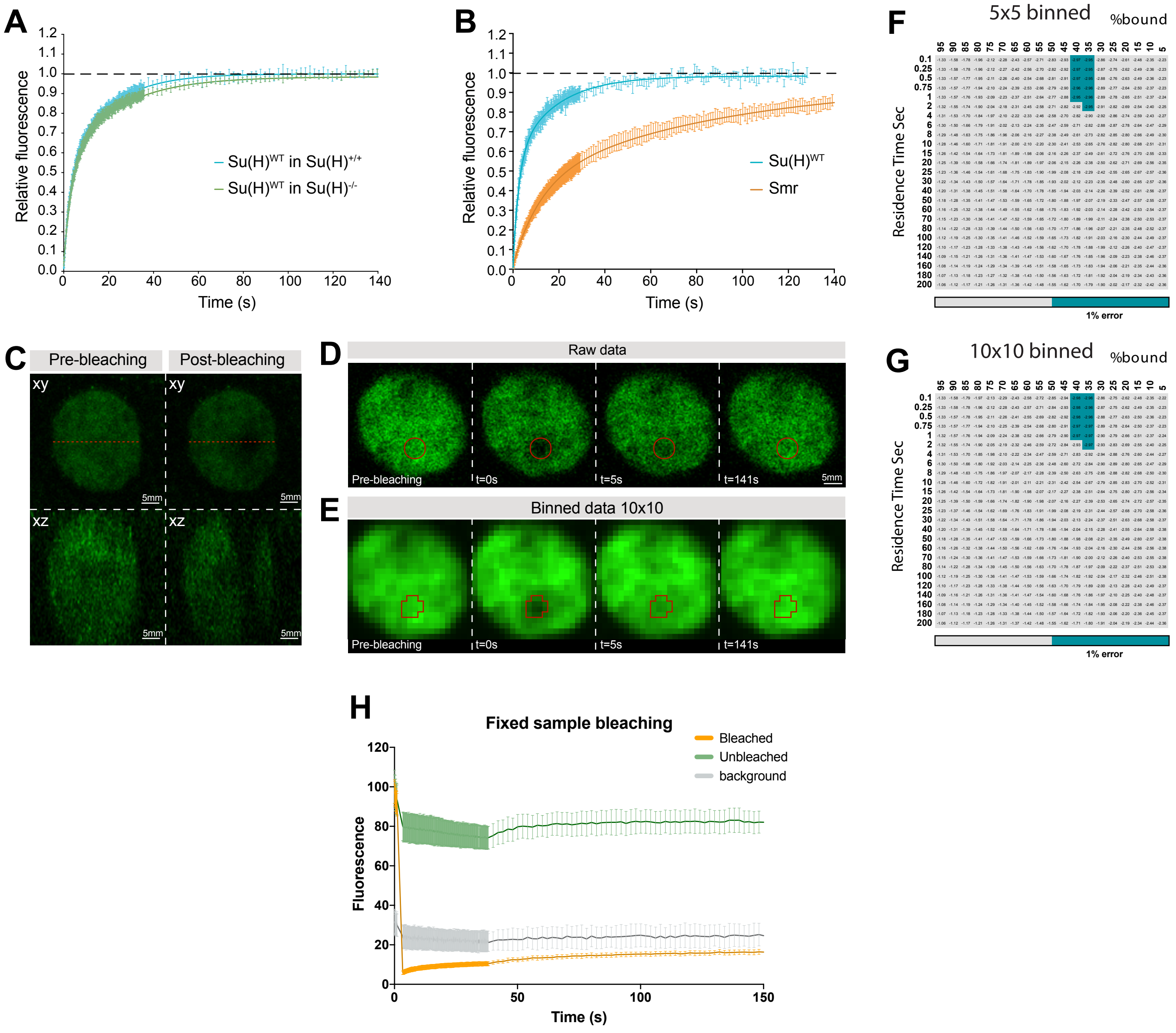
A
Relative fluorescence
Time (s)
Su(H)WT in Su(H)+/+
Su(H)WT in Su(H)-/-
B
Relative fluorescence
Time (s)
Su(H)WT
Smr
C
Pre-bleaching
Post-bleaching
xy
xz
5mm
D
Raw data
Pre-bleaching
t=0s
t=5s
t=141s
5mm
E
Binned data 10x10
Pre-bleaching
t=0s
t=5s
t=141s
F
5x5 binned
%bound
Residence Time Sec
1% error
G
10x10 binned
%bound
Residence Time Sec
1% error
H
Fixed sample bleaching
Bleached
Unbleached
background
Fluorescence
Time (s)

Figure S2; Related to Figure 1. FRAP profiles and extra controls, including SMRTER::YFP FRAP curve

(**A**) Comparison between FRAP curves obtained for Su(H)^{WT}-N-eGFP in a WT background or in a transheterozygote flies carrying two Su(H) null alleles, Su(H)^{SF8} and Su(H)^{A9}, following point-bleaching at random positions in the nuclei (Mean ± SEM). (**B**) FRAP curves obtained for the indicated proteins, following half nucleus bleaching (Mean ± SEM). (**C**) Bleaching profile following point-bleaching at random positions in the nucleus of a fixed sample. (**D-E**) Example images extracted at the indicated times from FRAP movies showing raw data (**D**) or 10x10 binned data for modelling (**E**). (**F-G**) Comparison of combinations of residence time and percentage of bound molecules giving best-fit to FRAP data, using the same image with 5x5 or 10x10 pixel binning before the modelling. Grey-blue indicates combinations with ≤1% error around the optimal value. (**H**) Whole cell bleaching in a fixed sample shows only a small contribution from reversible photobleaching to the recovery curve, with levels below the background signal (Mean ± SEM).

Figure S3

A

50ms exposure time

	State F1/F2	State F2/B2	State B1a	State B1b
Diff. Coef.	Blurred out 68.5%	0.4µm²/s	0.07µm²/s	0.02µm²/s
% of 10ms loc.		6.3	11	14.2
mean time		111ms	155ms	560ms

B

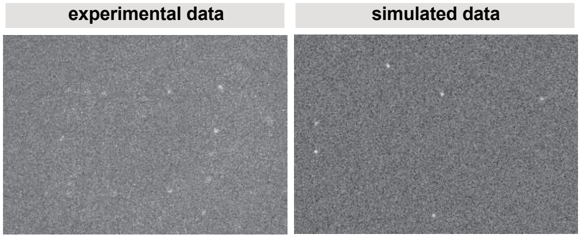

C

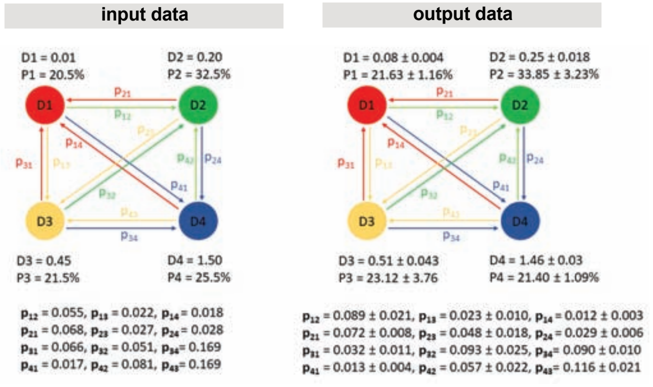

Figure S3; Related to Figure 2. Extra SMT analysis of Su(H)::mEOS.

(**A**) Table of Diffusion coefficients, proportions of molecules belonging to different groups and mean times for each state in Notch^{OFF} conditions, calculated as in (Persson et al., 2013) from 50ms data (no constraints), and adjusted for the proportion of molecules that were blurred out with this exposure setting (See Figure 2A,B) (**B**) Comparison between a real SMT image and a simulated one. (**C**) Diagram of Diffusion coefficients, proportions of molecules belonging to different groups and dwell times for each state in the simulations performed, calculated as in (Persson et al., 2013).

Figure S4

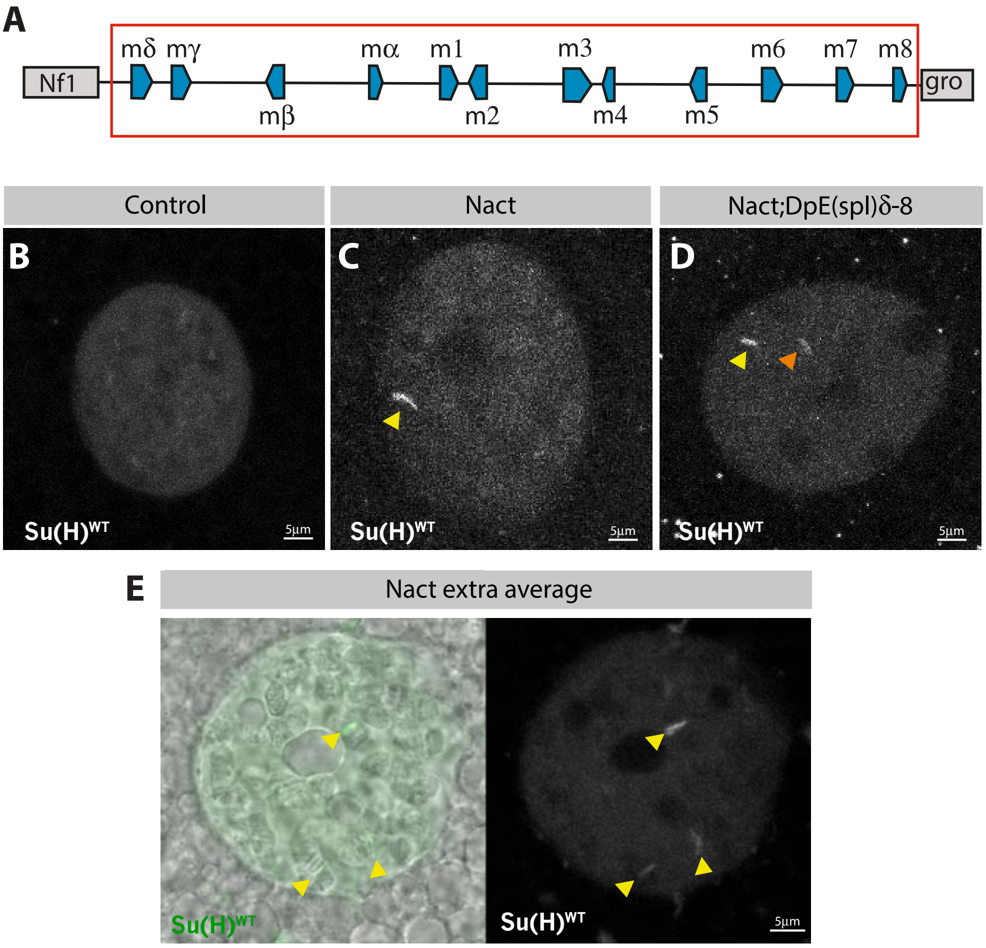
A
Nf1
mδ
mγ
mβ
mα
m1
m2
m3
m4
m5
m6
m7
m8
gro
Control
Nact
Nact;DpE(spl)δ-8
B
C
D
Su(H)WT
5µm
E
Nact extra average
Su(H)WT
Su(H)WT
5µm

Figure S4; Related to Figure 3. Ectopic site of Su(H):GFP recruitment in nuclei containing duplicated *E(spl)-C*.

(**A**) Schematic representation of the *E(spl)-C* genomic region, indicating the region included in the BAC, inserted in DpE(spl)∂-8. (**B-D**) Live imaging of Su(H)^{WT} in the conditions indicated. When the BAC is present, two sites of Su(H)^{WT}::GFP recruitment are detected in Notch ON cells, at the endogenous *E(spl)-C* (**D**, yellow arrowhead) and at the BAC copy (**D** orange arrowhead), compare to control cells with Notch only (**C**). Note the differing intensity of Su(H)::GFP bands due to only a single copy of the BAC being present. (**E**) Images showing extra SuH(H)::GFP bands observed in Notch ON cells.

Figure S5

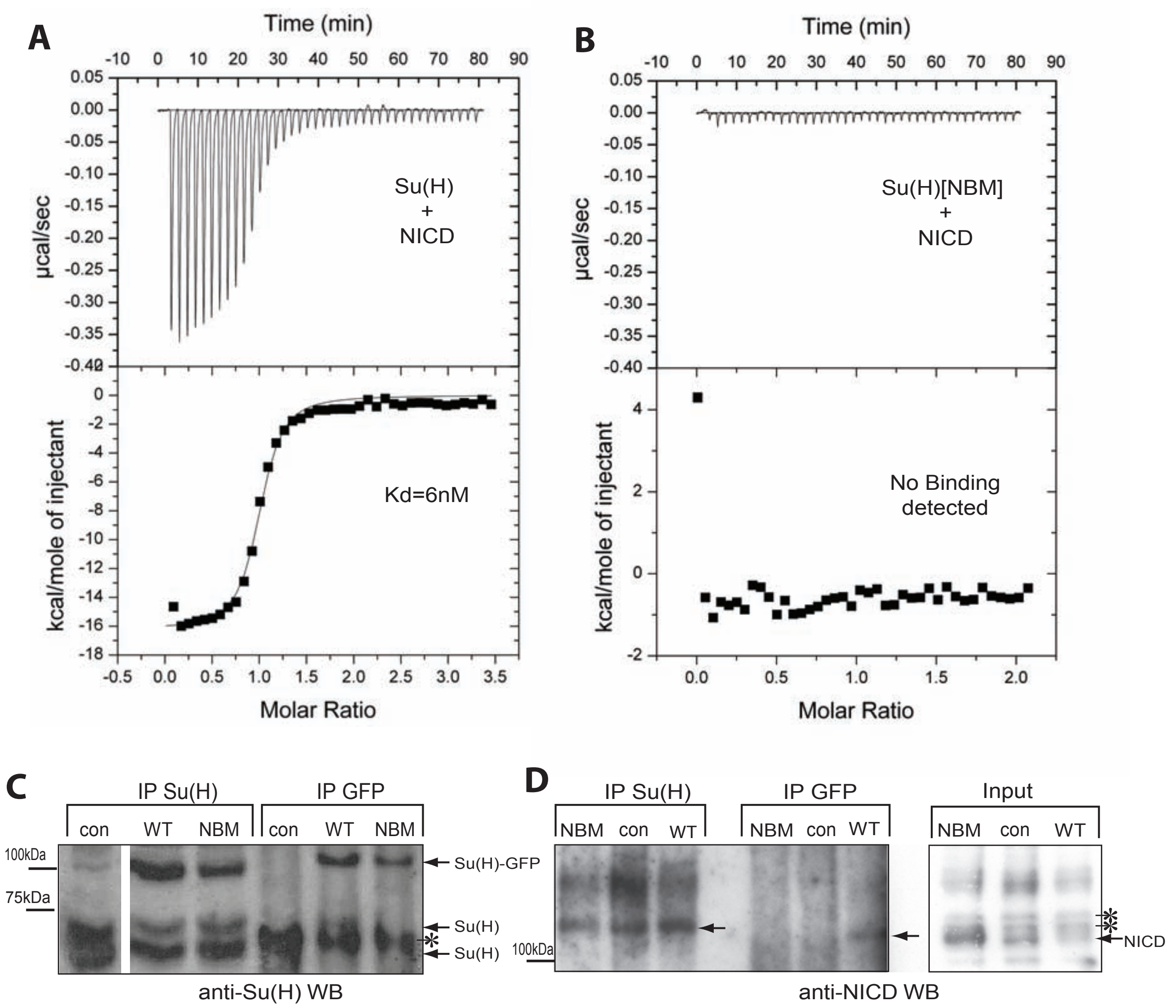
A
Time (min)
µcal/sec
Su(H)
+
NICD
kcal/mole of injectant
Kd=6nM
Molar Ratio
B
Time (min)
µcal/sec
Su(H)[NBM]
+
NICD
kcal/mole of injectant
No Binding
detected
Molar Ratio
C
IP Su(H)
IP GFP
con
WT
NBM
con
WT
NBM
100kDa
75kDa
Su(H)-GFP
Su(H)
*
Su(H)
anti-Su(H) WB
D
IP Su(H)
IP GFP
Input
NBM
con
WT
NBM
con
WT
NBM
con
WT
100kDa
NICD
anti-NICD WB

Figure S5; Related to Figure 4. Su(H)^{NBM} mutation blocks binding to NICD

(**A-B**) Isothermal calorimetry measurements of complexes produced between Su(H)^{WT} (**A**) or Su(H)^{NBM} (**B**) and NICD; with Su(H)^{NBM} no interaction is detected. (**C-D**) Co-immunoprecipitation (Co-IP) using anti-Su(H) or anti-GFP antibodies as indicated, and immunoblotted using anti-Su(H) (**C**) or anti-NICD (**D**). In C, positions for endogenous Su(H) or Su(H)::GFP are indicated by arrows, asterisk indicates non-specific band. In (**D**) NICD is indicated by arrows, and is not co-precipitated with Su(H)^{NBM}. Extracts for Co-IPs were prepared from larval heads with salivary glands.

Figure S6

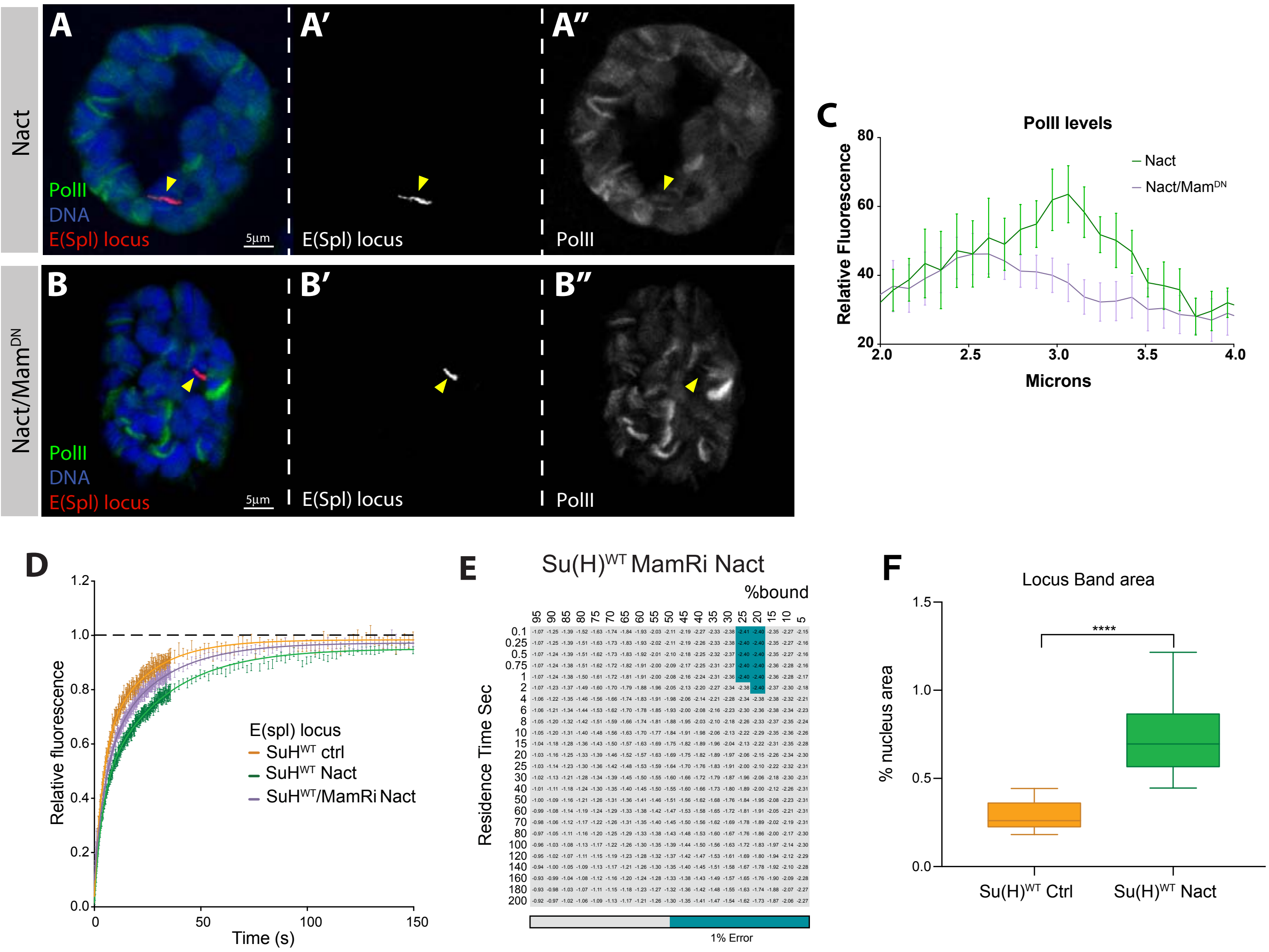

A
A′
A″
Nact
PolII
DNA
E(Spl) locus
5μm
E(Spl) locus
PolII
B
B′
B″
Nact/MamDN
PolII
DNA
E(Spl) locus
5μm
E(Spl) locus
PolII
C
PolII levels
Nact
Nact/MamDN
Relative Fluorescence
Microns
80
60
40
20
2.0
2.5
3.0
3.5
4.0
D
Relative fluorescence
Time (s)
E(spl) locus
SuHWT ctrl
SuHWT Nact
SuHWT/MamRi Nact
0.0
0.2
0.4
0.6
0.8
1.0
1.2
0
50
100
150
E
Su(H)WT MamRi Nact
%bound
Residence Time Sec
1% Error
F
Locus Band area
% nucleus area
****
Su(H)WT Ctrl
Su(H)WT Nact
0.0
0.5
1.0
1.5

Figure S6; Related to Figure 4. Effects of Mam depletion

(**A-B**) Co-localization of phosphorylated PolII (Ser10) in salivary gland cells with *E(spl)-C* locus tagged (arrows) in Notch ON cells. MamDN (**B**) impairs recruitment of PolII to *E(spl)-C*. (**C**) Quantification of PolII levels across *E(spl)-C* in indicated genotypes (n=19, Mean ± SEM). (**D**) FRAP curves obtained from focused point bleaching of $Su(H)^{WT}$ specifically at *E(spl)-C* in the conditions indicated (Mean ± SEM). (**E**) Combinations of residence time and percentage of bound molecules giving best-fit to FRAP data, with grey-blue indicating combinations with ≤1% error around the optimal value. (**F**) Quantification of the tagged E(spl) locus area, calculated as percentage of total nucleus area (n=11, Box and whiskers Min-to-Max, ****$p < 0.0001$, unpaired two-tailed t test).

Figure S7

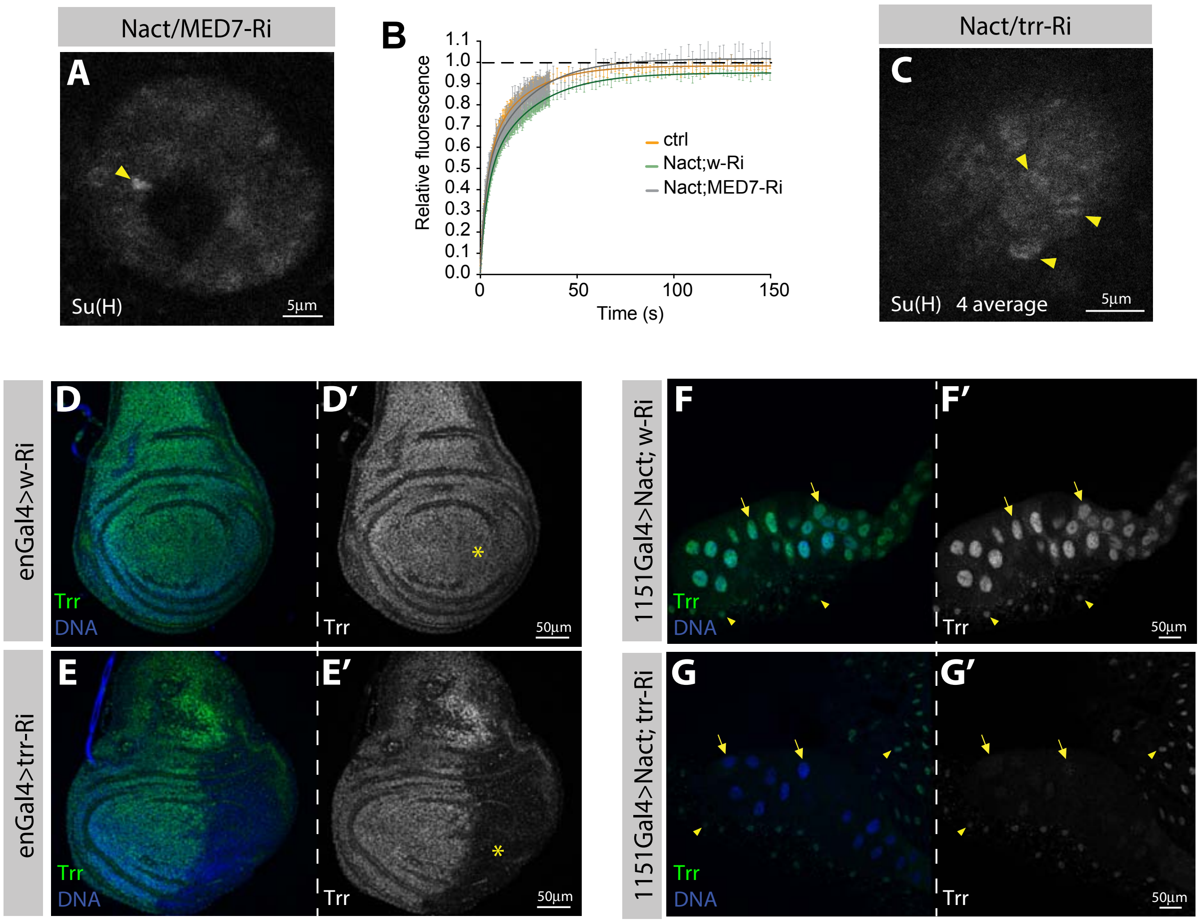

Nact/MED7-Ri
A
Su(H)
5µm
B
Relative fluorescence
Time (s)
ctrl
Nact;w-Ri
Nact;MED7-Ri
Nact/trr-Ri
C
Su(H) 4 average
5µm
enGal4>w-Ri
D
D'
Trr
DNA
Trr
50µm
enGal4>trr-Ri
E
E'
Trr
DNA
Trr
50µm
1151Gal4>Nact; w-Ri
F
F'
Trr
DNA
Trr
50µm
1151Gal4>Nact; trr-Ri
G
G'
Trr
DNA
Trr
50µm

Figure S7; Related to Figure 6. Effects of MED7 depletion and validation of trr-Ri

(**A**) Live imaging of Su(H)^{WT} in the conditions indicated. *MED7* knock-down does not affect Su(H)^{WT}::GFP recruitment in Notch ON cells. (**B**) Comparison between FRAP curves obtained for Su(H)^{WT}::GFP following point-bleaching at the *E(spl)-C* region in the genotypes indicated (Mean ± SEM). Note the faster recovery in the *MED7* KD, compared to the control Nact. (**C**) Live imaging of Su(H)^{WT} with extra averaging, in the conditions indicated, showed that there Su(H)::GFP is still recruited to chromosomes in *trr* KD. (**D-G**) Levels of Trr protein in the genotypes indicated (green in D-G, white in D'G'). *trr* KD reduces the levels of Trr in the wing disc when expressed in the posterior compartment (asterisk, **E**), compared to a WT disc (asterisk, **D**). Also in salivary glands, the expression of a *trr-Ri* reduces the levels of Trr in the polytene cells (arrows, **F-G**) but not in the surrounding fat body (arrowhead).

Supplementary Tables

Table S1; Related to Figure 2. Summary of SMT data analysis for Notch-OFF cells showing four diffusive states, their diffusion coefficients (D), dwell times, proportions and transition probabilities, computed using variational Bayesian treatment of Hidden Markov models based on (Persson et al., 2013).

	State B1	State B2	State F2	State F1	
**D[um^2/s]**					
E1	0.095611	0.25718	0.52097	1.8928	n=8180
E2	0.087384	0.2222	0.46726	1.8028	n=7792
E3	0.089451	0.20991	0.47016	1.8459	n=14064
E4	0.0787	0.2078	0.5302	2.0207	n=7340
**MEAN:**	**0.0877865**	**0.2242725**	**0.4971475**	**1.89055**	
**Proportion of molecules**					
E1	0.25784	0.34973	0.15989	0.23254	
E2	0.23394	0.33737	0.20399	0.2247	
E3	0.1536	0.29858	0.29634	0.25148	
E4	0.2138	0.3548	0.261	0.1704	
**MEAN:**	**0.214795**	**0.33512**	**0.230305**	**0.21978**	
**Av Time (s)**					
E1	0.085075	0.064843	0.036804	0.035313	
E2	0.10514	0.081294	0.034961	0.037498	
E3	0.07615	0.07574	0.046844	0.041131	
E4	0.105	0.088	0.041	0.033	
**MEAN:**	**0.09284125**	**0.07746925**	**0.03990225**	**0.0367355**	
**Transition probability per time step:**					
	**State B1**	**State B2**	**State F2**	**State F1**	
E1	0.88241	0.079774	0.020565	0.017251	*from B1*
	0.11601	0.84567	0.0016371	0.036683	*from B2*
	0.0054658	0.053628	0.7275	0.21341	*from F2*
	0.031774	0.11672	0.1353	0.71621	*from F1*
E2	0.9049	0.054931	0.022124	0.01804	*from B1*
	0.068136	0.87694	0.026886	0.028038	*from B2*
	0.066241	0.051301	0.71329	0.16917	*from F2*
	0.017287	0.081167	0.16881	0.73274	*from F1*
E3	0.86859	0.10375	0.0091448	0.018513	*from B1*
	0.071504	0.86792	0.046039	0.014535	*from B2*

	0.03207	0.065551	0.78635	0.11603	*from F2*
	0.018829	0.026434	0.19813	0.7566	*from F1*
E4	0.9053	0.0615	0.0328	0.0004	*from B1*
	0.0712	0.8868	0.012	0.03	*from B2*
	0.022	0.0964	0.7562	0.1254	*from F2*
	0.0277	0.0339	0.2329	0.7055	*from F1*
**Mean**	0.8903	0.07498875	0.02115845	0.013551	*from B1*
	0.0817125	0.8693325	0.021640525	0.027314	*from B2*
	0.0314442	0.06672	0.745835	0.1560025	*from F2*
	0.0238975	0.06455525	0.183785	0.7277625	*from F1*

Table S2; Related to Figure 2. Summary of SMT data analysis for Notch-OFF cells showing three diffusive states, their diffusion coefficients (D), dwell times, proportions and transition probabilities, computed using variational Bayesian treatment of Hidden Markov models based on (Persson et al., 2013).

	**State B1/B2**	**State F2**	**State F1**	
**D[um^2/s]**				
E1	0.10691	0.32626	1.7732	
E2	0.097838	0.30269	1.6751	
E3	0.12204	0.37456	1.765	
E4	0.098181	0.3352	1.7715	
**MEAN:**	**0.10624225**	**0.3346775**	**1.7462**	
**Proportion of molecules**				
E1	0.32154	0.41432	0.26414	
E2	0.30513	0.43579	0.25909	
E3	0.30707	0.41768	0.27525	
E4	0.33695	0.44183	0.22122	
**MEAN:**	**0.3176725**	**0.427405**	**0.254925**	
**Av Time (s)**				
E1	0.106	0.048726	0.04342	
E2	0.106	0.048726	0.04342	
E3	0.13349	0.05639	0.048199	
E4	0.12925	0.053746	0.044759	
**MEAN:**	**0.118685**	**0.051897**	**0.0449495**	
**Transition probability per time step:**				
	**State B1/B2**	**State F2**	**State F1**	
E1	0.90567	0.081629	0.012699	*from B1/B2*
	0.10964	0.79459	0.095769	*from F2*
	0.024477	0.20621	0.76931	*from F1*
E2	0.90921	0.07733	0.013456	*from B1/B2*
	0.10044	0.81258	0.086985	*from F2*
	0.019252	0.19342	0.78733	*from F1*
E3	0.92512	0.064868	0.01011	*from B1/B2*
	0.093392	0.82258	0.084028	*from F2*
	0.020624	0.18703	0.79234	*from F1*

E4	0.92267	0.075379	0.0019507	*from B1/B2*
	0.099438	0.81381	0.086752	*from F2*
	0.016253	0.20757	0.77618	*from F1*
**Mean**	0.9156675	0.0748015	0.009553925	*from B1/B2*
	0.1007275	0.81089	0.0883835	*from F2*
	0.0201515	0.1985575	0.78129	*from F1*

Table S3; Related to Figure 3. Summary of SMT data analysis for Notch-ON cells showing three diffusive states, their diffusion coefficients (D), dwell times, proportions and transition probabilities, computed using variational Bayesian treatment of Hidden Markov models based on (Persson et al., 2013).

	**State B1/B2**	**State F2**	**State F1**	
**D[um^2/s]**				
E1	0.1303	0.3923	1.5507	
E2	0.1291	0.3752	1.7563	
E3	0.1159	0.3379	1.7095	
E4	0.1365	0.3791	1.8279	
**MEAN:**	**0.12795**	**0.371125**	**1.7111**	
**Proportion of molecules**				
E1	0.3871	0.4328	0.18	
E2	0.3284	0.4008	0.2708	
E3	0.2604	0.4043	0.3352	
E4	0.2579	0.446	0.2961	
**MEAN:**	**0.30845**	**0.420975**	**0.270525**	
**Av Time (s)**				
E1	0.1493	0.0457	0.0327	
E2	0.1193	0.0452	0.0391	
E3	0.118	0.0479	0.0434	
E4	0.1063	0.0514	0.0387	
**MEAN:**	**0.123225**	**0.04755**	**0.038475**	
**Transition probability per time step:**				
	**State B1/B2**	**State F2**	**State F1**	
E1	0.933	0.0566	0.0103	*from B1/B2*
	0.115	0.7811	0.104	*from F2*
	0.0344	0.2717	0.6939	*from F1*
E2	0.9162	0.0628	0.021	*from B1/B2*
	0.1076	0.7788	0.1137	*from F2*
	0.0332	0.2223	0.7445	*from F1*
E3	0.9152	0.0637	0.0211	*from B1/B2*
	0.0802	0.7912	0.1285	*from F2*
	0.0256	0.2048	0.7696	*from F1*

E4	0.9059	0.084	0.01	*from B1/B2*
	0.0719	0.8055	0.1226	*from F2*
	0.0373	0.2209	0.7418	*from F1*
**Mean**	0.917575	0.066775	0.0156	*from B1/B2*
	0.093675	0.78915	0.1172	*from F2*
	0.032625	0.229925	0.73745	*from F1*

Table S4; Related to all figures. Table summarizing genotypes of flies used in each figure.

**Fig1**
Su(H)^{WT}-N-eGFP
Hairless^{WT}-C-eGFP
NRE-GFP
Fkh-GFP
Su(H)^{R266H}-N-GFP
1151-Gal4;;UAS-nls-GFP
Su(H)^{SF8};Su(H)^{WT}-N-eGFP x Su(H)^{A9};Su(H)^{WT}-N-eGFP

**Fig2**
Su(H)^{WT}-N-mEOS3.2

**Fig3**
1151-Gal4;; Su(H)^{WT}-N-GFP
1151-Gal4;; Su(H)^{WT}-N-GFP x UAS-N$^{\Delta ECD}$
1151-Gal4;; Su(H)^{R266H}-N-GFP x UAS-N$^{\Delta ECD}$
1151-Gal4;; Su(H)^{WT}-N-GFP,p31A-mCherry x m8intA
1151-Gal4;; Su(H)^{WT}-N-GFP,p31A-mCherry x UAS-N$^{\Delta ECD}$; m8intA
1151-Gal4;; Su(H)^{WT}-N-mEOS x UAS-N$^{\Delta ECD}$;Su(H)^{WT}-N-mEOS

**Fig 4**
1151-Gal4;; Su(H)^{WT}-N-GFP, p31A-mCherry x m8intA
1151-Gal4;; Su(H)^{WT}-N-GFP,p31A-mCherry x UAS-N$^{\Delta ECD}$; m8intA
1151-Gal4;; Su(H)^{WT}-N-mEOS,p31A-GFP x Su(H)^{WT}-N-mEOS,m8intA
1151-Gal4;; Su(H)^{WT}-N-mEOS,p31A-GFP x UAS-N$^{\Delta ECD}$; Su(H)^{WT}-N-mEOS,m8intA

**Fig5**
1151-Gal4;; Su(H)^{WT}-N-GFP x UAS-N$^{\Delta ECD}$
1151-Gal4;; Su(H)^{NBM}N-GFP x UAS-N$^{\Delta ECD}$
1151-Gal4;UAS-Mam^{DN} x UAS-N$^{\Delta ECD}$; Su(H)^{WT}-N-GFP
1151-Gal4;; Su(H)^{WT}-N-GFP x UAS-N$^{\Delta ECD}$;UAS-H-Ri
1151-Gal4;; Su(H)^{WT}-N-GFP x UAS-H-Ri
1151-Gal4;; Su(H)^{NBM}-N-GFP x UAS-N$^{\Delta ECD}$;UAS-H-Ri
1151-Gal4;; Su(H)^{WT},p31A-mCherry x m8intA
1151-Gal4;; Su(H)^{WT},p31A-mCherry x UAS-N$^{\Delta ECD}$; m8intA

**Fig6**
1151-Gal4;;p31A-mCherry x m8intA
1151-Gal4;;p31A-mCherry x UAS-N$^{\Delta ECD}$; m8intA

1151-Gal4;; Su(H)^{WT}-N-GFP x UAS-LacZ
1151-Gal4;; Su(H)^{WT}-N-GFP x UAS-N$^{\Delta ECD}$
1151-Gal4; Hairless-C-GFP x UAS-p31A-mCherry, m8intA
1151-Gal4; Hairless-C-GFP x UAS-N$^{\Delta ECD}$;UAS-p31A-mCherry, m8intA

**Fig7**
1151-Gal4;; Su(H)^{WT}-N-GFP x UAS-N$^{\Delta ECD}$
1151-Gal4;; Su(H)^{WT}-N-GFP x UAS-E(z)-RNAi
1151-Gal4;; Su(H)^{WT}-N-GFP x UAS-N$^{\Delta ECD}$;UAS-w-Ri
1151-Gal4;; Su(H)^{WT}-N-GFP x UAS-N$^{\Delta ECD}$;UAS-Trr-Ri
1151-Gal4;UAS-Mam^{DN} x UAS-N$^{\Delta ECD}$; Su(H)^{WT}-N-GFP

**SupFig1**
yw
Su(H)^{WT}-N-eGFP
Hairless^{WT}-C-eGFP
Su(H)^{R266H}-N-eGFP
Su(H)^{WT}-N-mEOS3.2

**SupFig2**
Su(H)^{WT}-N-eGFP
Su(H)^{SF8};Su(H)^{WT}-N-eGFP x Su(H)^{A9};Su(H)^{WT}-N-eGFP
Smr-YFP
Hairless-C-GFP

**SupFig3**
Su(H)^{WT}-N-mEOS3.2

**SupFig4**
1151-Gal4;;Su(H)^{WT}-N-GFP
1151-Gal4;; Su(H)^{WT}-N-GFP x UAS-N$^{\Delta ECD}$
1151-Gal4;DpE(Spl)mδ-m8 x UAS-N$^{\Delta ECD}$;Su(H)^{WT}-N-GFP

**SupFig5**
yw
Su(H)^{WT}-N-GFP
Su(H)^{NBM}-N-GFP

**SupFig6**
1151-Gal4 x UAS-N$^{\Delta ECD}$;p31A-mcherry, m8intA
1151-Gal4;UAS-Mam^{DN} x UAS-N$^{\Delta ECD}$; p31A-mCherry, m8intA

1151-Gal4;; Su(H)^{WT},p31A-mCherry x m8intA
1151-Gal4;; Su(H)^{WT},p31A-mCherry x UAS-N$^{\Delta ECD}$; m8intA
1151-Gal4;UAS-Mam^{DN} x UAS-N$^{\Delta ECD}$; Su(H)^{WT}-N-GFP

**SupFig7**
1151-Gal4;; Su(H)^{WT} x UAS-N$^{\Delta ECD}$; MED7-Ri
1151-Gal4;; Su(H)^{WT} x UAS-N$^{\Delta ECD}$; trr-Ri
1151-Gal4;; Su(H)^{WT} x UAS-N$^{\Delta ECD}$; w-Ri
en-Gal4, Gal80ts;w-Ri
en-Gal4, Gal80ts;w-Ri x trr-Ri

Table S5; Related to STAR Methods. List of oligonucleotides.

Name	Sequence	Purpose
E(spl)-mα forward	GCAGGAGGACGAGGAGGATG	mRNA levels
E(spl)-mα reverse	GATCCTGGAATTGCATGGAG	mRNA levels
E(spl)-mβ forward	GCTGGACTTGAAACCGC	mRNA levels
E(spl)-mβ reverse	AGAAGTGAGCAGCAGCC	mRNA levels
E(spl)-m3 forward	AGCCCACCCACCTCAAC	mRNA levels
E(spl)-m3 reverse	GTCTGCAGCTCCATTAGTC	mRNA levels
Rpl32 forward	ATGCTAAGCTGTCGCACAAATG	mRNA levels
Rpl32 reverse	GTTCGATCCGTAACCGATGT	mRNA levels
Nextera PCR primer 1	AATGATACGGCGACCACCGAGATCTACACTCGTCGG CAGCGTCAGATGTG	ATAC
Nextera PCR primer 2.1	CAAGCAGAAGACGGCATACGAGATTCGCCTTAGTCT CGTGGGCTCGGAGA	ATAC
Nextera PCR primer 2.2	CAAGCAGAAGACGGCATACGAGATCTAGTACGGTCT CGTGGGCTCGGAG	ATAC
Nextera PCR primer 2.3	CAAGCAGAAGACGGCATACGAGATTTCTGCCTGTCT CGTGGGCTCGGAGA	ATAC
Nextera PCR primer 2.4	CAAGCAGAAGACGGCATACGAGATGCTCAGGAGTCT CGTGGGCTCGGAG	ATAC
Nextera PCR primer 2.5	CAAGCAGAAGACGGCATACGAGATAGGAGTCCGTCT CGTGGGCTCGGAGATGT	ATAC
Nextera PCR primer 2.6	CAAGCAGAAGACGGCATACGAGATCATGCCTAGTCT CGTGGGCTCGGAGA	ATAC
Nextera PCR primer 2.7	CAAGCAGAAGACGGCATACGAGATGTAGAGAGGTCT CGTGGGCTCGGAGATGT	ATAC
Nextera PCR primer 2.8	CAAGCAGAAGACGGCATACGAGATCCTCTCTGGTCT CGTGGGCTCGGAGA	ATAC
Nextera PCR primer 2.9	CAAGCAGAAGACGGCATACGAGATAGCGTAGCGTCT CGTGGGCTCGGAG	ATAC
Nextera PCR primer 2.10	CAAGCAGAAGACGGCATACGAGATCAGCCTCGGTCT CGTGGGCTCGGAGATGT	ATAC
Nextera PCR primer 2.11	CAAGCAGAAGACGGCATACGAGATTGCCTCTTGTCT CGTGGGCTCGGAGATGT	ATAC
Nextera PCR primer 2.12	CAAGCAGAAGACGGCATACGAGATTCCTCTACGTCT CGTGGGCTCGGAGATGT	ATAC
Nextera PCR primer 2.13	CAAGCAGAAGACGGCATACGAGATATCACGACGTCT CGTGGGCTCGGAGATGT	ATAC
Nextera PCR primer 2.14	CAAGCAGAAGACGGCATACGAGATACAGTGGTGTCT CGTGGGCTCGGAGATGT	ATAC
Nextera PCR primer 2.15	CAAGCAGAAGACGGCATACGAGATCAGATCCAGTCT CGTGGGCTCGGAGATGT	ATAC
Rab11 intron forward	ACTGAAAATGGGCCGTTTCG	ATAC
Rab11 intron reverse	AGGAGTGGTAATCGACGGTC	ATAC
Eip78C EcR forward	AGAAGTAGGGGCCGTCAAGT	ATAC

Eip78C EcR reverse	GTGTAAGACCCGTCGCATTT	ATAC
Negative 1 forward	GCATTTTTGTGGCAGAGGCA	ATAC
Negative 1 reverse	CTCTTTCGGTGTCGCCTTCT	ATAC
Mst87F forward	ATCCTTTGCCTCTTCAGTCC	ATAC
Mst87F reverse	AATAATGATACAAAATCTGGTTACGC	ATAC
mβ gene forward	AGAAGTGAGCAGCAGCCATC	ATAC
mβ gene reverse	GCTGGACTTGAAACCGCACC	ATAC
mβ peak forward	AGAGGTCTGTGCGACTTGG	ATAC
mβ peak reverse	GGATGGAAGGCATGTGCT	ATAC
mα peak forward	AAGCCAGTGGACTCTGCTCT	ATAC
mα peak reverse	TGATCTCCAAGCGGAGTATG	ATAC
mα gene forward	GCAGGAGGACGAGGAGGATG	ATAC
mα gene reverse	GATCCTGGAATTGCATGGAG	ATAC
m3 peak forward	ACACACACAAACACCCATCC	ATAC
m3 peak reverse	CGAGGCAGTAGCCTATGTGA	ATAC
m3 gene forward	CGTCTGCAGCTCAATTAGTC	ATAC
m3 gene reverse	AGCCCACCCACCTCAACCAG	ATAC
m8 gene forward	CAATTCCACGAAGCACAGTC	ATAC
m8 gene reverse	GAGGAGCAGTCCATCGAGTT	ATAC
