# Supplementary material for: Activation of the Notch Signaling Pathway In Vivo Elicits Changes in CSL Nuclear Dynamics
Source: Dev Cell. 2018 Mar 12;44(5):611–623.e7. doi: 10.1016/j.devcel.2018.01.020 (PMC5855320; doi:10.1016/j.devcel.2018.01.020)
Supplement: Document S2. Article plus Supplemental Information [file mmc4.pdf]

Article

Developmental Cell

Activation of the Notch Signaling Pathway *In Vivo* Elicits Changes in CSL Nuclear Dynamics

Highlights

- Live imaging reveals that CSL binds very dynamically in Notch-OFF conditions
- Recruitment of CSL is enhanced in Notch-ON cells, and its residence time is increased
- Notch locally increases chromatin accessibility to promote loading of CSL complexes
- Longer CSL dwell times are conferred by the Notch-co-activator Mastermind

Authors

Maria J. Gomez-Lamarca, Julia Falo-Sanjuan, Robert Stojnic, ..., Kevin O'Holleran, Rhett Kovall, Sarah J. Bray

Correspondence

sjb32@cam.ac.uk

In Brief

Gomez-Lamarca et al. explore the basis of Notch signaling responsiveness at the transcriptional level through *in vivo*, real-time analysis of key DNA-binding factor CSL. CSL is highly dynamic with transient DNA residence in the Notch-OFF state. Notch activity increases chromatin accessibility and confers a longer dwell time for response sensitivity.

Check for updates

Gomez-Lamarca et al., 2018, Developmental Cell *44*, 611–623
March 12, 2018 © 2018 The Authors. Published by Elsevier Inc.
https://doi.org/10.1016/j.devcel.2018.01.020

CellPress

Developmental Cell
Article

Activation of the Notch Signaling Pathway *In Vivo* Elicits Changes in CSL Nuclear Dynamics

Maria J. Gomez-Lamarca,[1] Julia Falo-Sanjuan,[1,6] Robert Stojnic,[1,6] Sohaib Abdul Rehman,[2] Leila Muresan,[2] Matthew L. Jones,[1] Zoe Pillidge,[1] Gustavo Cerda-Moya,[1] Zhenyu Yuan,[3] Sarah Baloul,[1] Phillippe Valenti,[4] Kerstin Bystricky,[5] Francois Payre,[4] Kevin O'Holleran,[2] Rhett Kovall,[3] and Sarah J. Bray[1,7,*]

[1]Department of Physiology Development and Neuroscience, University of Cambridge, Downing Street, Cambridge CB2 3DY, UK
[2]Cambridge Advanced Imaging Centre, University of Cambridge, Downing Street, Cambridge CB2 3DY, UK
[3]University of Cincinnati College of Medicine, Department of Molecular Genetics, Biochemistry and Microbiology, 231 Albert Sabin Way, Cincinnati, OH 45267-0524, USA
[4]Centre de Biologie du Développement/UMR5547, CBI (Centre de Biologie Intégrative) University of Toulouse/CNRS, 118 Rte de Narbonne, 31062 Toulouse, France
[5]LBME/UMR5099, CBI (Centre de Biologie Intégrative) University of Toulouse/CNRS, 118 Rte de Narbonne, 31062 Toulouse, France
[6]These authors contributed equally
[7]Lead Contact
*Correspondence: sjb32@cam.ac.uk
https://doi.org/10.1016/j.devcel.2018.01.020

SUMMARY

A key feature of Notch signaling is that it directs immediate changes in transcription via the DNA-binding factor CSL, switching it from repression to activation. How Notch generates both a sensitive and accurate response—in the absence of any amplification step—remains to be elucidated. To address this question, we developed real-time analysis of CSL dynamics including single-molecule tracking *in vivo*. In Notch-OFF nuclei, a small proportion of CSL molecules transiently binds DNA, while in Notch-ON conditions CSL recruitment increases dramatically at target loci, where complexes have longer dwell times conferred by the Notch co-activator Mastermind. Surprisingly, recruitment of CSL-related corepressors also increases in Notch-ON conditions, revealing that Notch induces cooperative or "assisted" loading by promoting local increase in chromatin accessibility. Thus, *in vivo* Notch activity triggers changes in CSL dwell times and chromatin accessibility, which we propose confer sensitivity to small input changes and facilitate timely shut-down.

INTRODUCTION

Notch is the receptor in a highly conserved cell-cell signaling pathway, whose normal function is essential both during development and throughout adult life (Borggrefe and Oswald, 2009; Bray, 2016; 2006; Kopan and Ilagan, 2009). In addition, aberrations in Notch signaling underpin many diseases and are causal in some types of cancers (Nowell and Radtke, 2017; Ntziachristos et al., 2014). Understanding the mechanism transducing Notch activity is thus of widespread relevance. Canonical Notch signaling results in release of the Notch intracellular domain (NICD), which directly interacts with a DNA-binding protein called Suppressor of Hairless in flies (Su(H), also known as CBF1 or Lag-1 in other species), generally referred to as CSL and, together with the co-activator Mastermind (Mam), triggers the transcription of target genes (Bray, 2016; 2006; Kopan and Ilagan, 2009).

In the absence of Notch, CSL works as a repressor through a different set of partners, including the co-repressor Hairless (Barolo et al., 2002; Kulic et al., 2015; Morel et al., 2001). The prevailing, yet untested, model is that, following Notch activation, NICD displaces co-repressors, while CSL remains bound to DNA (Borggrefe and Oswald, 2009). Recent evidence has challenged this model, as the affinity of NICD for CSL is similar to that of the co-repressors, making it unclear how it could displace them (Collins et al., 2014; VanderWielen et al., 2011; Yuan et al., 2016). In addition, the occupancy level of CSL complexes at target loci differs in Notch-ON versus Notch-OFF conditions, as deduced from chromatin immunoprecipitation assays in fixed samples (Castel et al., 2013; Krejci and Bray, 2007; Wang et al., 2014), even though there is no evidence for any change in DNA affinity or specificity conferred by NICD (Del Bianco et al., 2010; Wilson and Kovall, 2006). At some loci with appropriately paired motifs, dimerization between NICD molecules could contribute to enhanced binding (Arnett et al., 2010; Hass et al., 2015), but many loci lack the appropriate sites for this to occur. Existing concepts thus fail to explain how both levels and duration of NICD signal are quantitatively integrated to confer proper transcriptional outputs (Dallas et al., 2005; Delaney, 2005; Guentchev and McKay, 2006; Mazzone et al., 2010). These considerations argue that an alternative model is needed and highlight that an understanding of Notch signal transduction will require quantifying the dynamics of Notch nuclear effectors, ideally in an *in vivo* system. To tackle this problem, we engineered a comprehensive series of *in vivo* molecular tools for the live analysis of Su(H), the *Drosophila* CSL, to determine its behavior and binding in Notch-OFF and Notch-ON conditions.

This is an open access article under the CC BY license (http://creativecommons.org/licenses/by/4.0/).

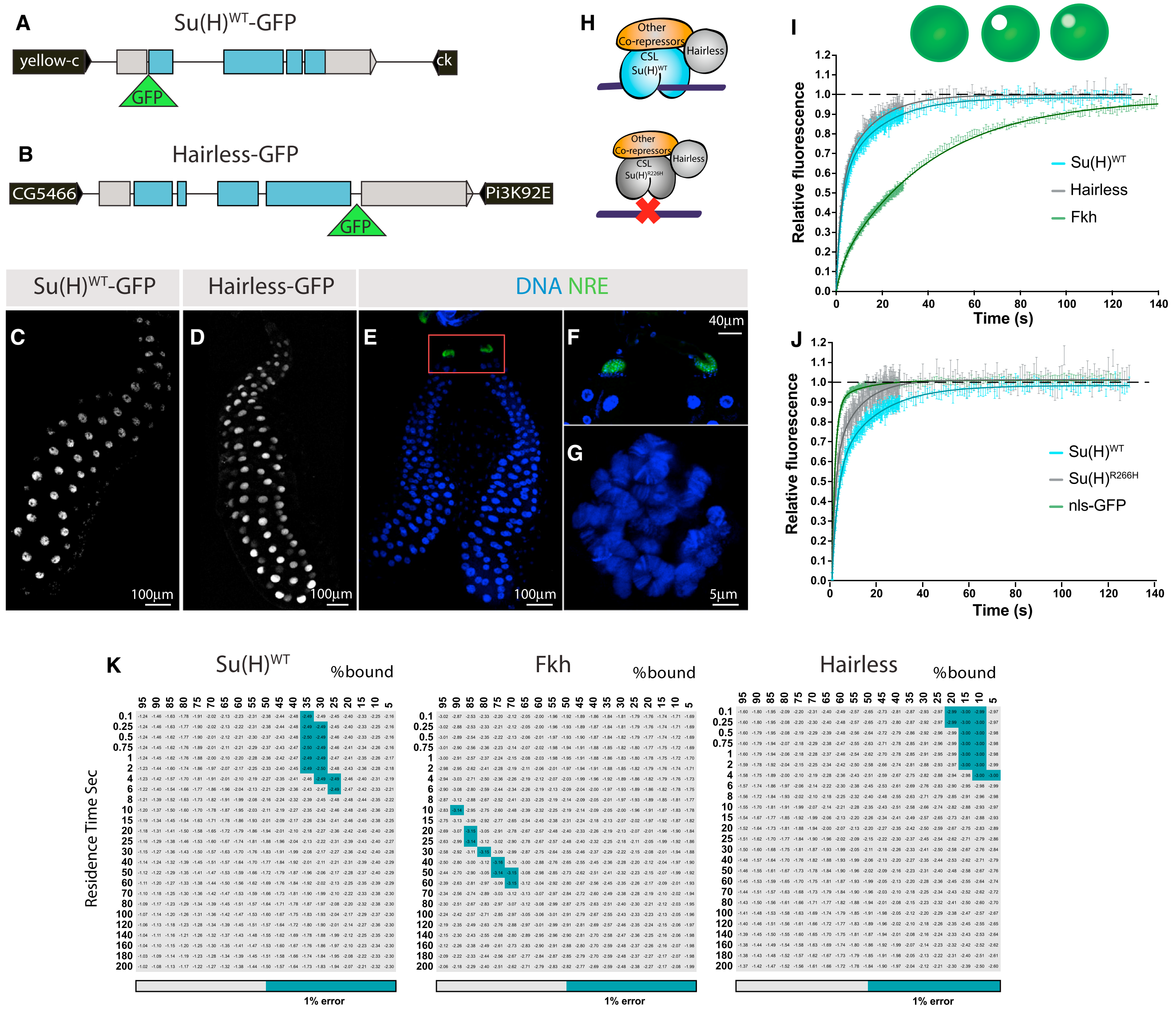

Figure 1. Su(H) and Hairless Display Fast Nuclear Dynamics

(A and B) Schematic of GFP-tagged genomic rescue constructs for Su(H) (A) and Hairless (B).

(C and D) Salivary glands with nuclear Su(H)WT::GFP (C) and Hairless::GFP (D).

(E–G) Salivary gland with NRE-GFP expression (green) and DNA staining (blue); higher magnifications show NRE-GFP expression in ring cells (F) and single nucleus with polytene chromosomes (G).

(H) Diagram illustrating wild-type (top) and R266H (bottom) Su(H) co-repressor complexes.

(I and J) FRAP curves obtained for the indicated proteins, following point-bleaching at random positions in the nuclei. Mean ± SEM.

(K) Combinations of residence time and percentage of bound molecules giving best-fit to FRAP data, with grey-blue indicating combinations with $\leq$1% error around the optimal value. Note that Su(H)WT refers to Su(H)WT::GFP in a Su(H) mutant background.

See also Figures S1 and S2.

RESULTS

Su(H) Is Transiently Bound to DNA in Notch-OFF Conditions

As a first step toward visualizing the dynamics of Notch nuclear effectors in living tissues, we generated EGFP-tagged transgenes of both *Su(H)* and its co-repressor *Hairless* (Figures 1A and 1B). Both fusions, here referred to as Su(H)::GFP and Hairless::GFP for simplicity, recapitulated endogenous expression and rescued to viability null mutants for the cognate gene (Figures 1C, 1D, and S1). To analyze the dynamics of Su(H) and Hairless, we first performed fluorescence recovery after photobleaching (FRAP) (van Royen et al., 2009), using point-bleaching directed at a random position in each nucleus. We took advantage of larval salivary glands that display large nuclei, where Notch is normally OFF, as shown by the absence of Notch reporter expression (Figures 1E–1G). Strikingly, FRAP data showed that CSL repressor complexes are highly dynamic.

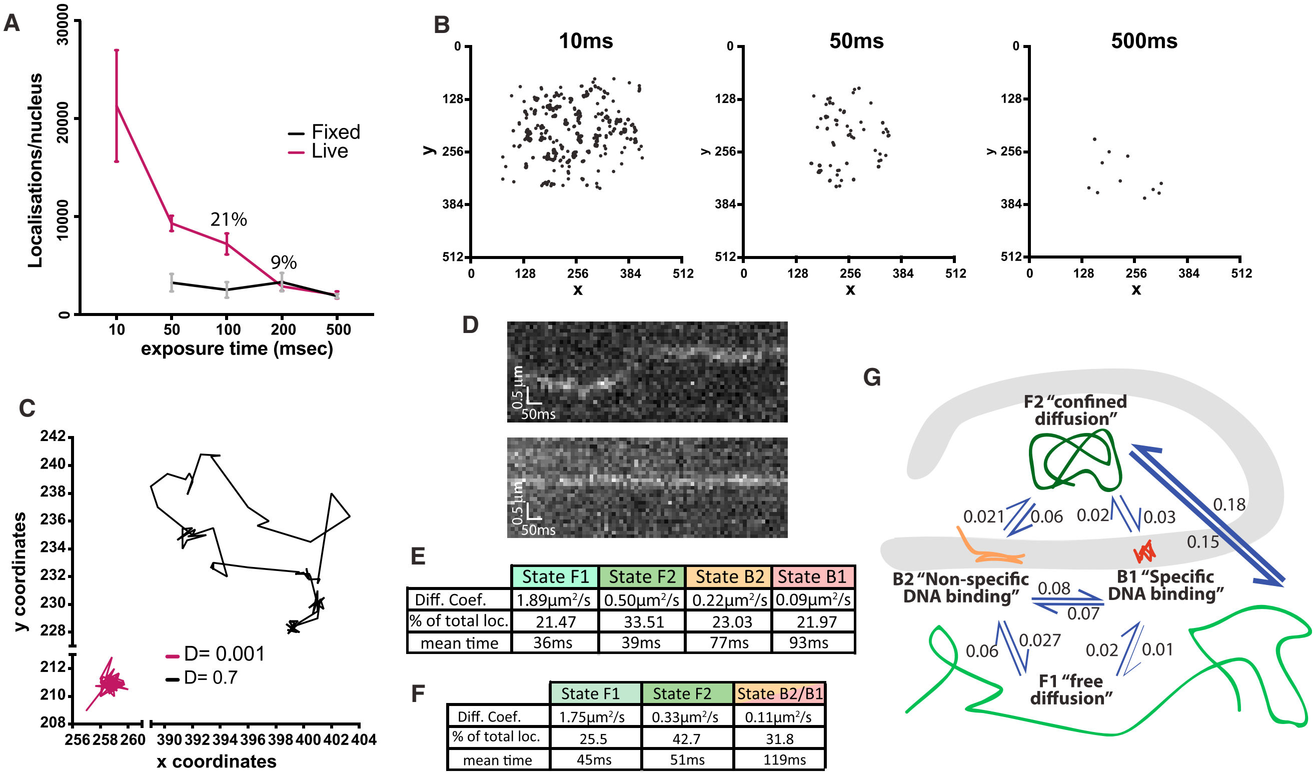

E

	State F1	State F2	State B2	State B1
Diff. Coef.	1.89μm²/s	0.50μm²/s	0.22μm²/s	0.09μm²/s
% of total loc.	21.47	33.51	23.03	21.97
mean time	36ms	39ms	77ms	93ms

F

	State F1	State F2	State B2/B1
Diff. Coef.	1.75μm²/s	0.33μm²/s	0.11μm²/s
% of total loc.	25.5	42.7	31.8
mean time	45ms	51ms	119ms

Figure 2. SMT Analysis of Su(H) Molecules Show that Few Have Longer Residence Times

(A and B) Motion-blurring experiment. (A) Su(H)::mEOS localizations per nucleus detected with different exposure times after photoconversion remain constant in fixed samples (black) and decrease in live samples (red), due to blurring of moving molecules in longer exposure times. (B) Examples of Su(H)::mEOS localizations detected during 1 s imaging in live samples using different exposure times. n = 7 nuclei/exposure time; mean ± SEM.

(C) Tracks of single Su(H)::mEOS molecules with distinct diffusion coefficients imaged with 10 ms exposure time.

(D) x-t kymographs of molecules in (C).

(E and F) Tables of diffusion coefficients and proportions of molecules belonging to different groups with mean times for each state, calculated as in (Persson et al., 2013) from 10 ms data either with no constraints (E, four states) or constrained to three states (F). Note that the percentage bound in (F) is less than the sum of B1 and B2 in (E), because high diffusion coefficient outliers assigned to B2 in the four-state system will be assigned to F2 in the three-state model.

(G) Schematic representation of different behaviors from (E), indicating the probabilities of switching states.

See also Tables S1 and S2, Figure S3 and Movies S1 and S2.

Su(H)::GFP recovery time (t1/2 = 3.6 s) was indeed considerably faster than that of Forkhead::GFP (Fkh::GFP, t1/2 = 24.4 s), a lineage-specific transcription factor (Figure 1I). Similar fast dynamics were also observed for Hairless::GFP (Figure 1I) but were not merely a property of repressors, since the unrelated co-repressor SMRTER exhibited much slower recovery (Figure S2). A mutation in Su(H) that abrogates its DNA-binding affinity *in vitro* (Su(H)R266H; Figure S1) led to an even faster recovery time (t1/2 = 1.9 s) when assayed *in vivo* (Figure 1J), showing that the dynamics of wild-type Su(H)/Hairless complexes encompass DNA-binding events. In contrast, Su(H)::GFP recovery in homozygous mutant background was similar to controls, indicating that the presence of unlabelled Su(H) has minimal impact. Thus, in Notch-OFF conditions, Su(H) normally undergoes transient DNA residency, which must nevertheless be sufficient for any repression it confers (Barolo et al., 2002; Kulic et al., 2015; Morel et al., 2001; Morel and Schweisguth, 2000).

FRAP kinetics depend on two distinct parameters: (1) the relative proportions of diffusible versus DNA-bound molecules and (2) the time each molecule remains bound to DNA (residence time). We therefore used a reaction-diffusion model to infer these parameters (see the STAR Methods; Figure S2), first estimating the diffusion constant of the unbound molecules from the recovery of the non-binding Su(H)R266H (D = 2.2 $\mu m^2/s$; see the STAR Methods). Fkh FRAP data were best fit by models where >75% Fkh molecules were bound to DNA, with a residence time of 30–60 s (Figure 1K). In contrast, optimal models for Su(H) implied that only 25%–35% molecules were bound, with residence times of 0.5–2 s (Figure 1K). Hairless residence time was similar to Su(H), albeit with a higher proportion of free molecules (<20% bound; Figure 1K). Thus, when compared with Fkh, a relatively small fraction of Su(H) and Hairless molecules are bound to DNA at any one time and they have considerably shorter residence times.

To further investigate Su(H) properties, we performed single-molecule tracking (SMT) of photo-convertible Su(H)::mEOS in live salivary glands (Figure 2). Containing the monomeric mEOS3.2, Su(H)::mEOS can be irreversibly photo-converted from green to red emission in response to 405 nm light. For our SMT assays, only a small proportion of the total Su(H)::mEOS

population was photo-converted so that single red emitting molecules could be detected and individually tracked. Freely diffusing (or very transiently bound) molecules become "blurred" in long exposure times (>50 ms) (Etheridge et al., 2014), while stationary molecules, such as those bound to DNA, remain resolved. Indeed we found that a modest proportion (21%) of photo-converted Su(H)::mEOS molecules stayed unblurred with 100 ms exposure, consistent with a low abundance of Su(H) repressor complexes bound to DNA (Figures 2A and 2B). Analysis of SMT from shorter exposure times (10 ms) further revealed that individual Su(H) complexes exhibit different patterns of mobility (Figures 2C and 2D; Movies S1 and S2) and when the characteristics from all the 10 ms tracks were extracted (using variational Bayes SPT; [Persson et al., 2013]) they revealed that Su(H) can transition through at least four different states (Figures 2E and 2G; Table S1). These range from "freely diffusing" (state F1; 21%, D = 1.89 $\mu m^2/s$), which had a similar diffusion constant to that estimated for the non-binding Su(H) R266H from FRAP, to essentially immobile molecules, likely engaged in specific DNA binding (state B1; 22% molecules, D = 0.09 $\mu m^2/s$). Intermediate states may relate to molecules whose diffusion is confined by obstacles (e.g., chromosomes; state F2, D = 0.50 $\mu m^2/s$) and to those undergoing non-specific interactions with the chromatin (state B2; D = 0.22 $\mu m^2/s$). When the model was constrained to three states, B1 and B2 coalesced into a single "bound" population that accounted for 32% of Su(H) molecules (Figure 2F; Table S2), i.e., a value within the range inferred from FRAP.

SMT results also suggested a very short dwell time (~100 ms) for Su(H)-bound molecules, below that estimated from FRAP. However, short exposure SMT is likely to underestimate dwell times, because of gaps in tracking caused by transient disappearance of molecules and because of photobleaching. Indeed, we detected some B1 tracks >400 ms, indicative of longer residence time for some molecules. Furthermore, data extracted from intermediate exposure times (50 ms) that circumvent some of those losses led to a mean dwell time of 560 ms, at the low end of the range inferred from the FRAP (Figure S3). This suggests that the estimates from the 10 ms data are curtailed by photobleaching. However, as relatively few tracks exhibited stationary behavior throughout (<2%), it is unlikely that the residence times are greatly in excess of those inferred from 50 ms SMT data, which are approaching those obtained from FRAP.

Altogether, these *in vivo* data indicate that one-third, at best, of Su(H) molecules are specifically bound to DNA and display a remarkably short dwell time, revealing the very dynamic binding of Su(H) complexes in Notch-OFF conditions.

Notch-ON Increases the Recruitment and Dwell Time of Su(H) Complexes

Having established that CSL complexes are highly dynamic in Notch-OFF cells, we then assayed whether Notch activity influences their binding properties, as suggested by chromatin immunoprecipitation experiments in both *Drosophila* and mammalian cells (Castel et al., 2013; Krejci and Bray, 2007; Wang et al., 2014). Hence, we analyzed Su(H) behavior in tissues supplied with activated Notch. Unexpectedly we found, both by FRAP and by SMT, that there was no global change in properties of Su(H) (Figures 3A and 3B; Table S3). However, there was a striking change in Su(H) intra-nuclear distribution in the live-imaged nuclei, from a general diffuse distribution in Notch-OFF nuclei to one with a prominent chromosomal band in Notch-ON nuclei (Figures 3C and 3D; note that several less prominent bands were also detected, Figure S4). The polytene chromosomes of salivary glands contain hundreds of aligned DNA copies, favoring visualization of transcription factor-binding events (Lis, 2007). The prominent band observed for Su(H) was thus likely representing recruitment of Su(H) at a target locus following Notch activation. Consistently, no such band was detected with the Su(H)R266H mutant unable to bind DNA (Figure 3E). These data thus clearly reveal that, *in vivo*, Notch modifies the dynamics of Su(H) chromosomal interactions in a way that is DNA binding dependent.

To further investigate this conclusion, we used complementary systems to manipulate and visualize a key chromosomal target of Su(H). In flies, the *Enhancer of split-Complex* (*E(spl)-C*) contains multiple highly responsive Notch target genes, making this an attractive candidate for the prominent Su(H) recruitment. To investigate this, we first examined nuclei bearing an extra transgenic copy of *E(spl)-C* (Chanet et al., 2009). These displayed a second strong Su(H) band (Figure S4), as predicted if the *in vivo* recruitment of Su(H) is occurring at *E(spl)-C*. Second, we implemented a DNA-tagging technique based on the ParB/*int* prokaryotic system, which allows visualization of a given locus in living cells (Saad et al., 2014; P.V., M.L.J., S.J.B. and F.P., unpublished data). Briefly, the ParB protein binds to a small DNA segment (*int*) and spreads along adjacent DNA, creating robust ParB foci that can be detected in fluorescence microscopy. We used CRISPR/Cas9 genome editing to introduce *int* within the distal part of *E(spl)-C*, and generated transgenic constructs for the targeted expression of ParB::mCherry. When driven in salivary cells, ParB specifically bound to the tagged locus, producing a strong signal that allowed us to specifically visualize the *E(spl)-C.* The Su(H) band in Notch-ON nuclei clearly co-localized with the tagged locus (Figures 3F–3H), demonstrating unequivocally that Su(H) is recruited to *E(spl)-C* in Notch-ON conditions.

The locus-tag provided us with a powerful way to focus photobleaching specifically on *E(spl)-C,* so that we could compare the recovery in Notch-OFF and Notch-ON conditions at a Notch-regulated target. Strikingly, the FRAP recovery for Su(H)::GFP was significantly slower in Notch-ON conditions compared with Notch-OFF (Figure 4A). Modeling the behavior of Su(H)::GFP around the *E(spl)-C* locus revealed a dramatic increase in the inferred residence times in Notch-ON nuclei, which approached 10–15 s (Figure 4B). We note that the overall proportion of bound molecules was largely unaffected, indicating that only a small fraction of Su(H)::GFP complexes change their behavior. Similarly, by performing SMT in Notch-ON nuclei with the tagged *E(spl)-C* locus we also revealed a striking enrichment for stationary (D = 0.08 $\mu m^2/s$) Su(H) molecules around the tagged *E(spl)-C* locus (Figures 4C–4G). Indeed the proportion of molecules that had properties of specific binding (B1) were almost doubled, with 38% B1 tracks at the locus versus 20% B1 tracks outside this region.

Taken together these data therefore provide compelling evidence that Notch increases the recruitment of Su(H) complexes

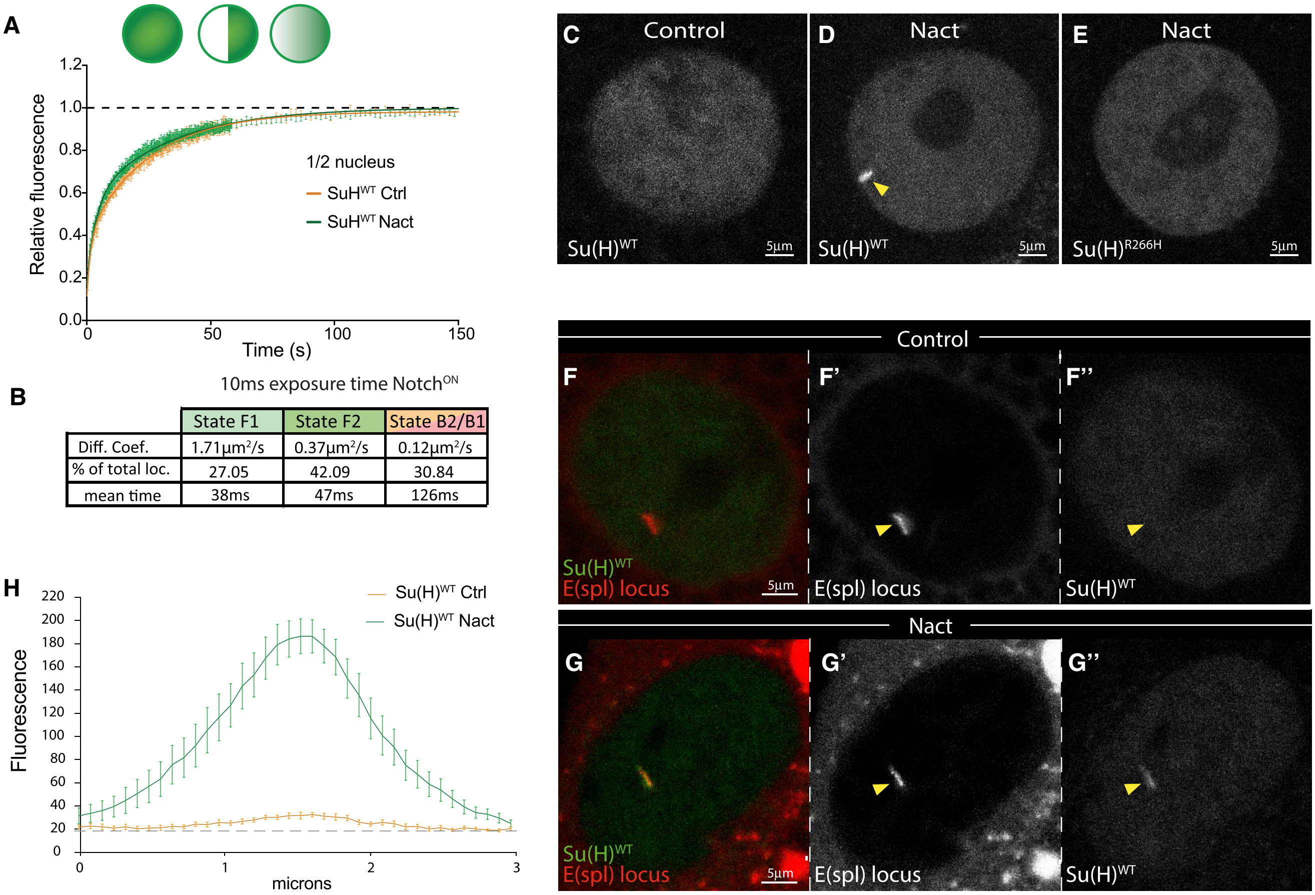

Figure 3. Notch Enhances Recruitment of Su(H) to *E(spl)-C*

(A) FRAP curves obtained from half nuclei bleaching of Su(H)WT in Notch-OFF and Notch-ON conditions. Mean ± SEM.

(B) Table of diffusion coefficients and proportions of photo-converted Su(H)::mEOS molecules belonging to different groups with mean times for each state, calculated as in (Persson et al., 2013) from 10 ms data in Notch-ON nuclei, n > 20,000 tracks.

(C–E) Live imaging of Su(H)::GFP. Chromosomal recruitment of Su(H)WT is stimulated by active Notch (D) (arrowhead), compared to control cells (C), and is abolished in Su(H)R266H (E). Nact refers to expression of constitutively active Notch, NΔecd.

(F–G″) Su(H) recruitment—green in (F) and (G), white in (F″) and (G″)—is localized to *E(spl)-C*, labeled by ParB::RFP—red in (F) and (G), white in (F′) and (G′) (see yellow arrowheads).

(H) Quantification of Su(H)::GFP intensity across the *E(spl)-C* locus. n = 11; mean ± SEM.

See also Figure S4.

to *E(spl)*-C target genes. Furthermore, our results demonstrate that Notch activity can change the dynamics of Su(H)-complex binding at target loci.

Differential Effects of NICD and Mam on Su(H) Recruitment and Dwell Times

We next sought to investigate the mechanisms underlying the increased Su(H) recruitment and dwell times in response to Notch activity. As a first step, we assessed whether Su(H) recruitment at *E(spl)*-C directly relies on its interaction with NICD, by engineering a Su(H) variant (NBM) in which five critical NICD-binding residues were mutated. While these mutations do not impinge on Hairless interaction (Yuan et al., 2016), they eliminate binding to NICD as assayed both *in vitro* and *in vivo* (Figure S5). Recruitment of Su(H)NBM to *E(spl)-C* was greatly compromised in Notch-ON nuclei, as evident from decreased GFP intensity and faster FRAP recovery (Figures 5B, 5C, 5E, and 5J). Indeed, the behavior of Su(H)NBM in Notch-ON cells resembled that of un-mutated Su(H) in Notch-OFF cells, with residence times of <1 s (Figure 5K). Despite its reduced residence, a low level of residual recruitment of Su(H)NBM to *E(spl)-C* was still evident in Notch-ON nuclei. We hypothesize that this is an indirect effect of NICD brought to the locus by endogenous untagged Su(H), as discussed further below.

Second, as a means to assay whether co-activators could stabilize Su(H) occupancy upon Notch activation, we co-expressed a dominant negative form of Mam (MamDN; Figure 5A). Mam normally recruits p300/CBP and other factors, forming a higher-order complex necessary to promote transcription (Fryer et al., 2002; Wallberg et al., 2002), although it is not essential to stabilize the Su(H)-NICD complex in *Drosophila* as it is in mammalian cells (Contreras et al., 2015). As in other contexts, when expressed in the salivary gland MamDN interfered with endogenous Mam, preventing polymerase II recruitment and

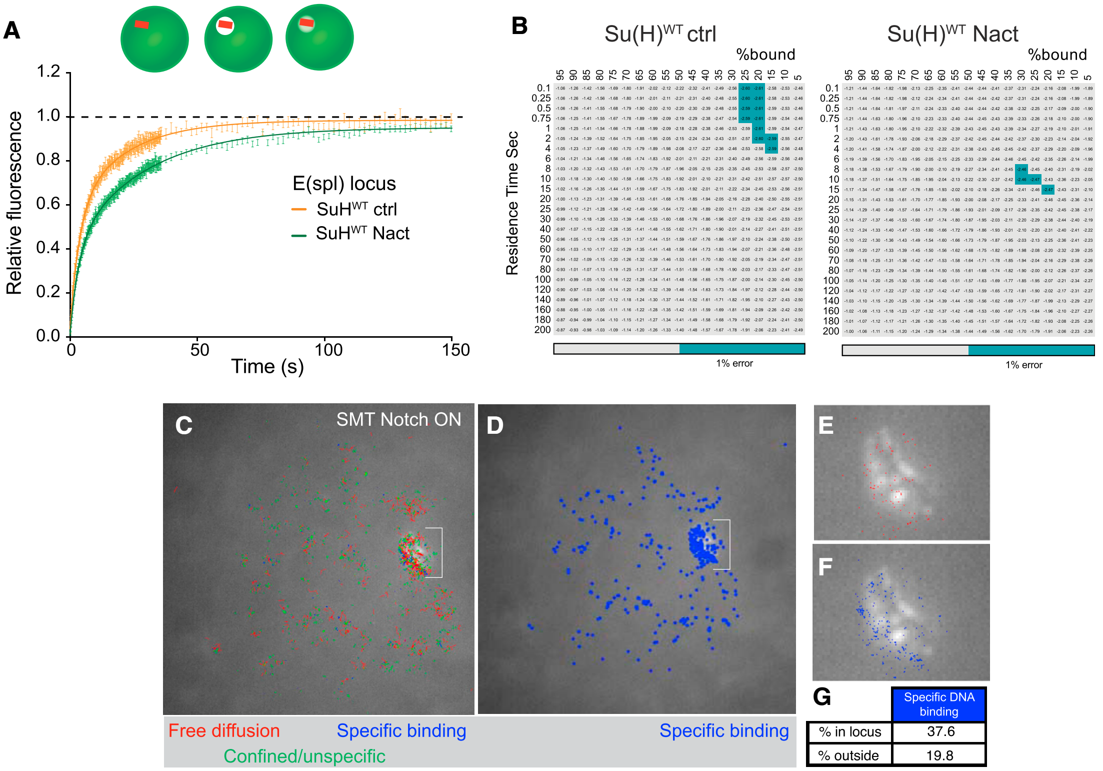

Figure 4. Notch Increases Su(H) Dwell Times at *E(spl)-C*

(A and B) FRAP curves obtained from focused point bleaching of Su(H)WT specifically at *E(spl)-C* in the conditions indicated (A). Mean ± SEM. Combinations of residence time and percentage of bound molecules giving best-fit to FRAP data, with grey-blue indicating combinations with $\leq$1% error around the optimal value (B).

(C–G) SMT tracks in Notch-ON nuclei in relation to *E(spl)-C* locus, labeled by ParB::GFP (white) and indicated by brackets in (C) and (D). (C) Distribution of SMT tracks of indicated types relative to *E(spl)-C* locus, with (D) indicating locations of specific binding tracks only. (E and F) Higher magnification of locus region and locations of freely diffusing molecules (red) and specific binding molecules (blue). (G) Proportion of tracks with characteristics of specific DNA binding (D = 0.08 $\mu m^2/s$) in the locus region versus elsewhere in Notch-ON nuclei (n > 7,000 tracks).

blocking transcriptional activation of *E(spl)* genes (Figures 5I and S6) (Fryer et al., 2002; Helms et al., 1999; Nam et al., 2006; 2003; Wallberg et al., 2002; Wilson and Kovall, 2006). Unexpectedly, live imaging revealed that MamDN did not reduce levels of Su(H) at *E(spl)-C* (Figures 5B, 5D, and 5E), indicating that the increased recruitment of Su(H) *per se* is not dependent on a transcriptionally competent complex. Strikingly however, MamDN led to a faster recovery time of Su(H) after photobleaching at *E(spl)-C* in Notch-ON nuclei (Figure 5J), with the best fit models indicating residence times of <1 s, resembling those of Notch-OFF despite the presence of NICD (Figure 5K). Similar alterations in Su(H) dynamics were obtained when Mam levels were depleted by RNAi (Figure S6). Functional Mam is therefore necessary for the increased dwell time of Su(H) in Notch-ON conditions, arguing that it does not rely on NICD alone, but Mam appears dispensable for the enhanced recruitment.

NICD Promotes Chromatin Opening and Assisted Loading of Su(H) Complexes

The fact that Su(H)NBM but not MamDN (or Mam RNAi) reduced the amount of Su(H)::GFP recruited, argues that NICD also exerts an effect that is independent of Mam. If our interpretation that the residual Su(H)NBM enrichment in Notch-ON nuclei relies on endogenous untagged Su(H) that retains the capability to bind NICD is correct, then it implies that NICD exerts an indirect effect on Su(H) recruitment. This model would predict that one Su(H)-NICD complex can affect the behavior of another Su(H) molecule(s), whether or not bound to NICD. One way that NICD could evoke an indirect effect on Su(H) recruitment, is by promoting a more open chromatin conformation, as suggested by its effects on histone modifications in other cell types (Oswald et al., 2016; Skalska et al., 2015; Wang et al., 2014). In agreement, the volume occupied by the locus tag in Notch-ON nuclei was expanded, as expected for less-compact chromatin (Figure S6). Levels of H3K27 acetylation and H3K4 monomethylation, two histone modifications associated with active enhancers, were also significantly increased at *E(spl)-C* locus in Notch-ON nuclei (Figures 6A–6H). To investigate further we measured the openness of chromatin at *E(spl)-C* using the assay for transposase-accessible chromatin (ATAC) method. A substantial increase in accessibility was detected at several regions across *E(spl)-C* locus in Notch-ON nuclei (Figures 6I and 6J). Thus, one consequence of NICD recruitment is a localized chromatin remodeling, which makes the region more accessible and favors Su(H) binding.

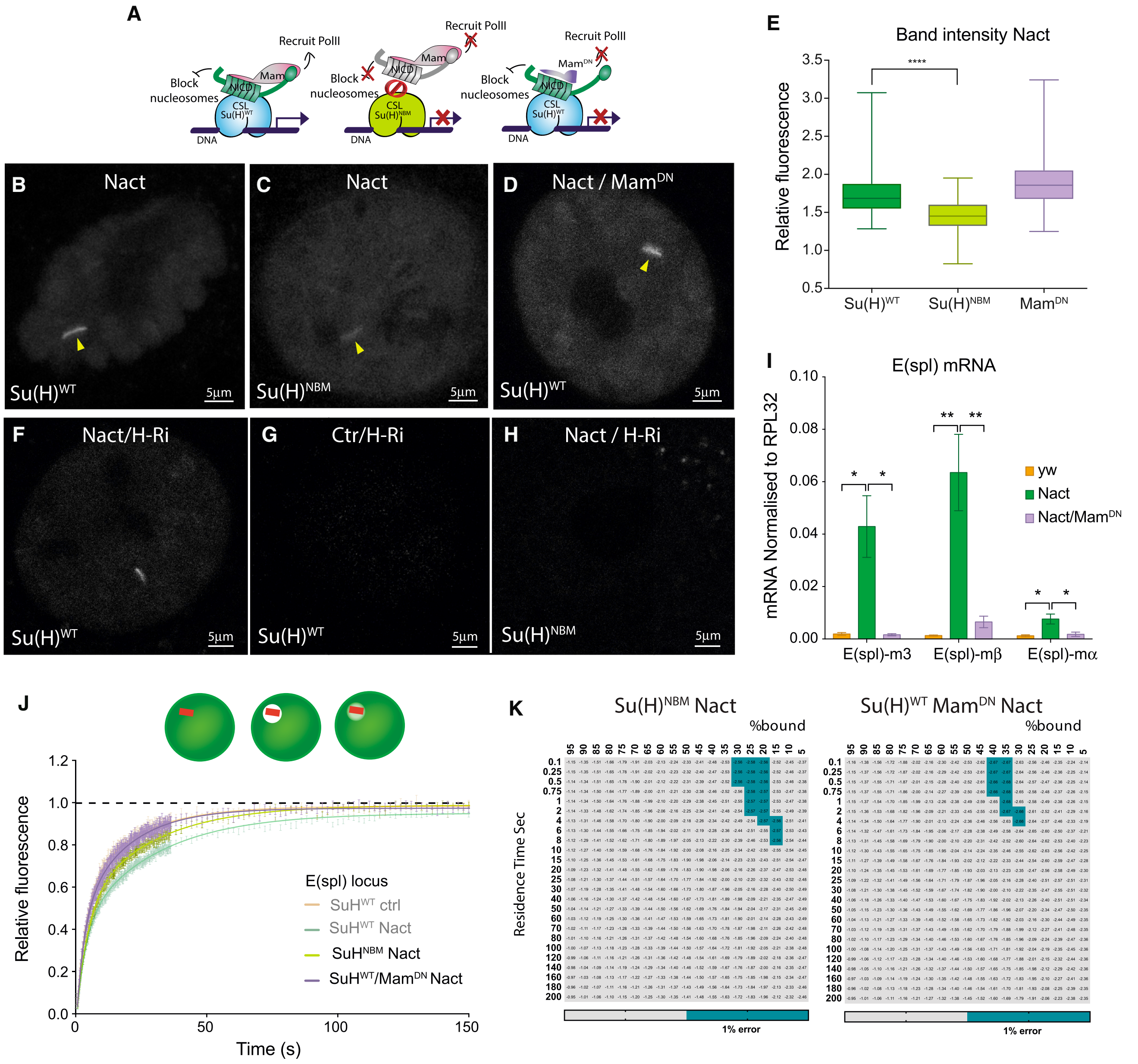

Figure 5. Effects of NICD and Mam on Su(H) Recruitment and Dwell Times

(A) Diagram of co-activator complexes, control (left), Su(H)NBM (middle), or MamDN (right).

(B–D) Live imaging of Su(H)WT (B and D) and Su(H)NBM (C) in Notch-ON cells. Su(H)NBM has reduced recruitment (C) (arrowhead) compared with Su(H)WT (B) (arrowhead). MamDN does not reduce Su(H)WT recruitment (D) (arrowhead).

(E) Relative fluorescence of Su(H) bands in the indicated genotypes (n > 35). Box and whiskers min to max; ****$p < 0.0001$, unpaired two-tailed t test.

(F–H) Live imaging of Su(H)WT (F and G) or Su(H)NBM (H) in the conditions indicated.

(I) As detected by qPCR of salivary gland samples, increased mRNA levels of three *E(spl)* genes in Notch-ON cells is prevented when MamDN is co-expressed. n = 3; mean ± SEM; *$p < 0.05$, **$p < 0.0332$, one-way ANOVA.

(J) FRAP curves obtained from focused point-bleaching of Su(H)WT specifically at the *E(spl)-C* locus in the conditions indicated. Mean ± SEM. Note: Notch-OFF and Notch-ON are the same as in Figure 4A.

(K) Combinations of residence time and percentage of bound molecules giving best-fit to FRAP data, with grey-blue indicating combinations with ≤1% error around the optimal value.

See also Figures S5 and S6.

If NICD promotes Su(H) recruitment by changing chromatin accessibility, it would not discriminate against Su(H) that is complexed with other factors, including the Hairless co-repressor. As opposed to current views, our model thus makes the surprising prediction that Hairless recruitment to target loci should also be enhanced following Notch activation. We therefore quantified

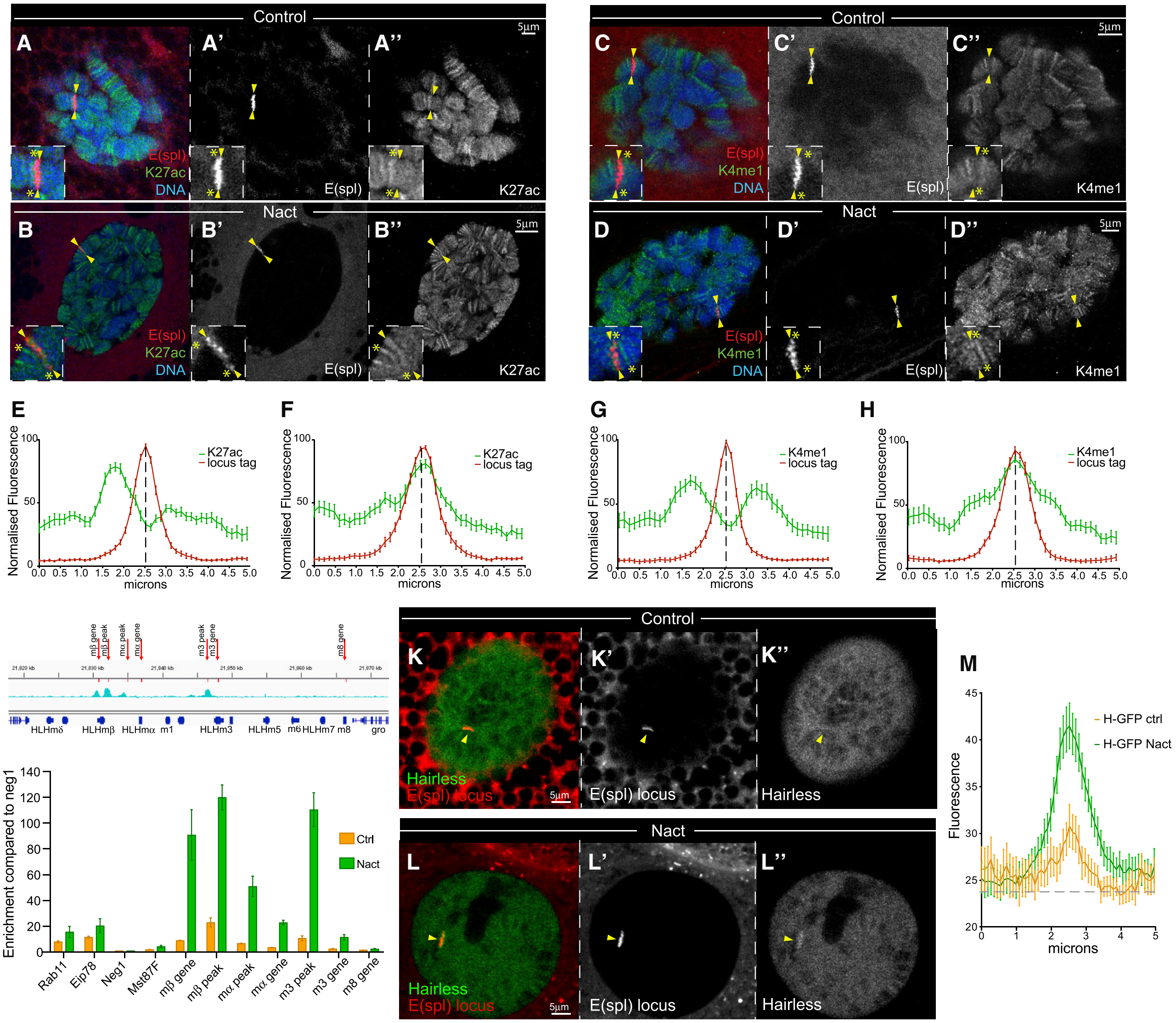

Figure 6. Notch Induces Chromatin Opening and Promotes Assisted Loading

(A–D″) Levels of H3K27ac—green in (A) and (B), white in (A″) and (B″)—and H3K4me1—green in (C) and (D), white in (C″) and (D″)—histone modifications at *E(spl)-C* (arrowheads) labeled by ParB::RFP—red in (A)–(D), white in (A′)–(D′)—in Control (A and C) and Notch-ON cells (B and D). Higher magnifications, insets in (A)–(D), show unchanged flanking loci (yellow asterisk) compared with increase at *E(spl)-C* locus (arrowheads).

(E–J) Quantification of the indicated histone modifications across the *E(spl)-C* locus, both H3K27ac and H3K4me1 are increased in Notch-ON cells. n > 25; mean ± SEM. (I–J) Chromatin accessibility across *E(spl)-C* (I) measured by enrichment of fragments to transposon tagging with ATAC (J); positions of primers used in (J) are indicated in (I) relative to the gene models (dark blue) and Su(H)-binding profile in Kc cells (cyan). Rab11 intron and Eip78C EcR are predicted open chromatin control regions, while Negative1 and Mst87F are closed chromatin control regions, none of which are subject to Notch regulation. Shown is the fold enrichment compared with the Neg1 control region. n = 3; mean ± SEM.

(K–L″) Increased Hairless-GFP recruitment—green in (K) and (L), white in (K″) and (L″)—at *E(spl)-C*—red in (K) and (L), white in (K′) and (L′)—in Notch-ON cells (L) (arrowhead) compared with Notch-OFF cells (K) (arrowhead).

(M) Quantification of Hairless::GFP intensity across *E(spl)-C*. n > 25; mean ± SEM.

Hairless levels at *E(spl)-C* in Notch-OFF versus Notch-ON conditions. Indeed, like Su(H), we measured a clear increase in Hairless levels upon Notch activation (Figures 6K–6M). Furthermore, the residual recruitment of Su(H)NBM observed in Notch-ON nuclei was abolished when Hairless was knocked down (Figures 5F and 5H), suggesting that it is Hairless-bound Su(H)NBM that is recruited. We noted, however, that the levels of Su(H) were strongly reduced in these conditions (Figures 5G and 5H), arguing that Hairless is necessary for Su(H) stability as reported recently (Yuan et al., 2016), which may have compromised our ability to detect any binding. Nevertheless, recruitment of un-mutated Su(H) was still obvious even in the Hairless-depleted Notch-ON nuclei (Figure 5F). Together the data indicate that NICD triggers a substantial change in the accessibility of its *E(spl)-C* target locus, and likely other target loci too, which favors recruitment of Su(H), affecting both the repressor and the activator complexes.

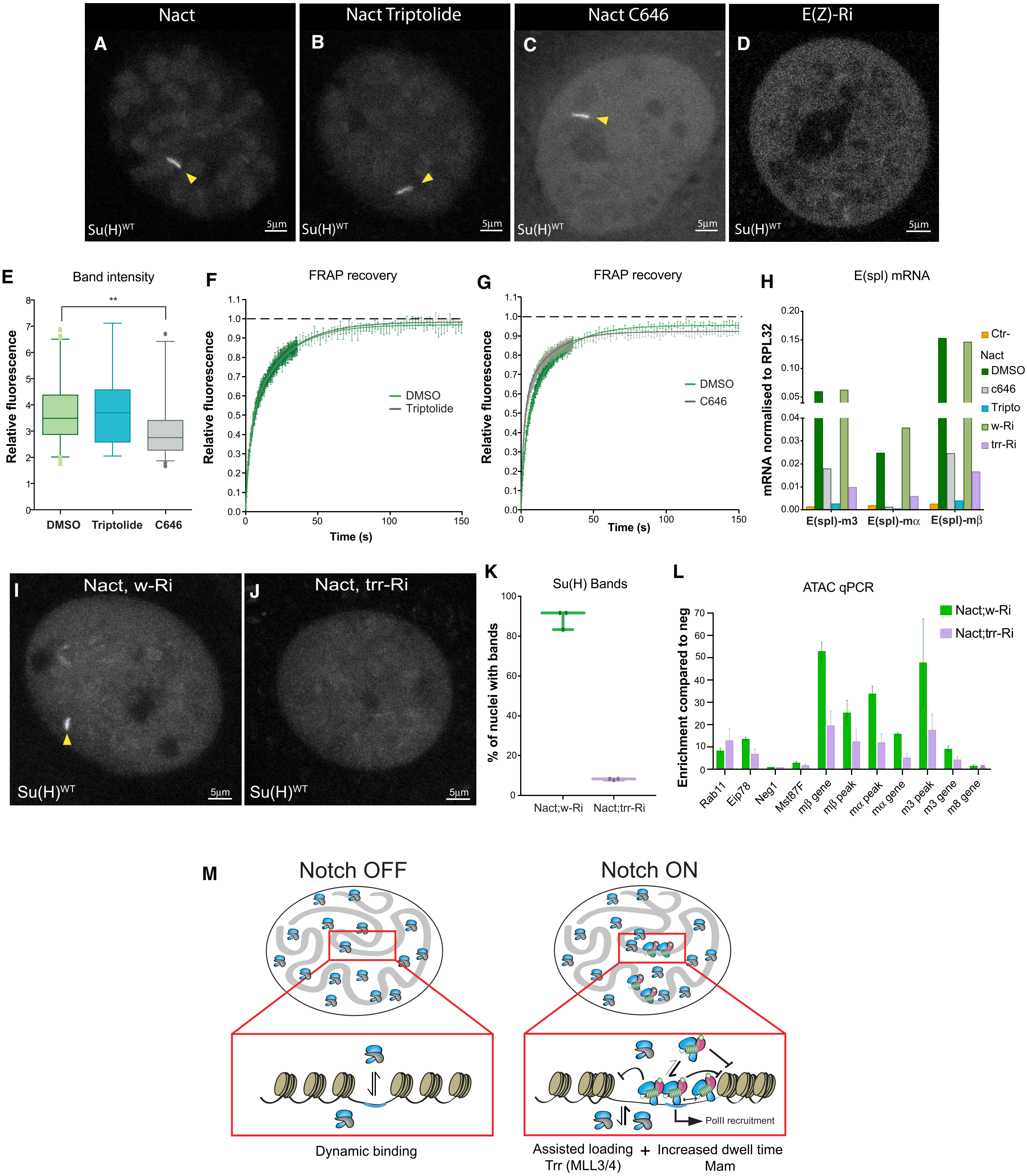

Figure 7. CBP and Trithorax-Related Are Required for Enhanced Su(H) Recruitment in Notch-ON Cells

(A–C) Live imaging of Su(H)WT in Notch-ON cells treated with DMSO (A), triptolide (10 μM) (B), or C646 CBP inhibitor (30 μM) (C). Yellow arrowheads indicate Su(H) recruitment.

(D) Live imaging of Su(H)WT in Notch-OFF cells treated with E(z) *RNAi*.

(E) Relative fluorescence of Su(H) bands in the indicated genotypes. n > 30; box and whiskers 5–95 percentile; **p < 0.0332, unpaired two-tailed t test.

(F and G) FRAP curves obtained from focused point-bleaching of Su(H)WT specifically at the band region in the conditions indicated. Mean ± SEM.

(H) Effects of the indicated treatments on up-regulation of *E(spl)* gene mRNA levels in Notch-ON cells, measured by RT-qPCR.

(legend continued on next page)

CBP and Trithorax-Related Contribute to NICD-Induced Changes in Su(H) Dynamics

We next asked whether the change in Su(H) recruitment elicited by NICD was an indirect consequence of increased transcription initiation, by testing the effects of triptolide, a potent inhibitor of the TFIIH complex required for promoter opening (Krebs et al., 2017) Indeed, triptolide treatment abolished the NICD-induced expression of *E(spl)-C* mRNAs (Figure 7H). However, triptolide had no effect on the levels of Su(H) recruitment or FRAP recovery in Notch-ON cells (Figures 7A, 7B, and 7E–7F), indicating that these events are independent of transcription initiation. Earlier steps in pre-initiation complex formation involve the Mediator complex and the acetyltransferase CBP/p300, which is also responsible for H3K27 acetylation and which has been shown to interact with Mam (Fryer et al., 2002; Wallberg et al., 2002). To test whether CBP was necessary for the change in Su(H) dynamics, we treated Notch-ON tissues with C646, a potent inhibitor of CBP catalytic activity that causes its dissociation from chromatin (Boija et al., 2017). Although CBP inhibition resulted in a significant decrease in the expression of *E(spl)-C* mRNAs (Figure 7H), it produced only a modest decrease in recruitment of Su(H) (Figures 7C and 7E). The C646 treatment also slightly modified Su(H) FRAP recovery kinetics in Notch-ON tissues, indicating that the Su(H)-binding dynamics were altered (Figure 7G), but the effects were less pronounced than occurred with MamDN. Similar change in FRAP occurred when Mediator function was perturbed by depleting levels of its Med7 subunit, suggesting that Mediator contributes to the increase in dwell time (Figure S7). These results, together with the fact that there is still a clear band in Notch-ON cells treated with C646, demonstrate that CBP activity is not sufficient to account for all the changes in Su(H) behavior in Notch-ON cells, and they suggest it is likely to have an important role at subsequent steps in Notch induced transcription.

Key factors involved in the interplay between active and inactive enhancers include members of Polycomb (repressive) and Trithorax (activating) complexes (Schuettengruber et al., 2017). Reasoning that activity of these complexes might be important for Su(H) recruitment in Notch-ON cells, we examined the consequences of their RNAi-mediated depletion. First, we asked whether loss of repressive complexes could phenocopy Notch-ON conditions, i.e., facilitate Su(H) recruitment in Notch-OFF nuclei. However, depletion of repressive Polycomb group members (*Enhancer of zeste [E(z)]*, *Polycomb*, *polyhomoetic proximal*, and *pipsqueak*) was not sufficient to promote a detectable Su(H) "band" in Notch-OFF cells (e.g., Figure 7D), nor could *E(z)* depletion further enhance recruitment in Notch-ON conditions (data not shown). Second, we tested the converse hypothesis, i.e., that chromatin regulators of the activating Trithorax group (e.g., *ash1*, *Trithorax-like,* and *trithorax-related*) would be required for Su(H) recruitment in Notch-ON nuclei. Of those tested, only depletion of Trithorax-related (Trr), the *Drosophila* orthologue of MLL3/4 H3K4 mono-methylase (also known as KTM2D), produced a discernible effect. Strikingly, the Su(H) enrichment in Notch-ON cells was abolished in the Trr-depleted nuclei (Figures 7I–7K and S7) as was the increase in *E(spl)-C* mRNA expression, which resembled that in Notch-OFF conditions (Figure 7H). Likewise, residual chromosomal Su(H) recruitment still occurred in Trr-depleted cells, even though the enriched "band" was lost (Figures 7J and S7). Finally, ATAC assays indicated that the chromatin accessibility at *E(spl)-C* in Notch-ON cells was also reduced in the absence of Trr (Figure 7L), more resembling that in Notch-OFF tissue (Figure 6J), a result that fits well with evidence that MLL3/4 promotes chromatin opening at enhancers in mammalian cells (Dorighi et al., 2017) and positively regulates Notch outputs in pre-T cells (Oswald et al., 2016).

DISCUSSION

Until recently, most existing models have portrayed CSL as a molecule with long DNA residence that serves as a static platform for exchange between NICD and co-repressors. Our analysis, using a combination of FRAP and SMT to measure Su(H) dynamics, reveals a very different story and highlights two important characteristics. First, in Notch-OFF conditions, Su(H) normally undergoes very transient DNA residency, despite the fact that it is important for repression of the target loci. This implies that prolonged binding is not a prerequisite for repression. It also argues against a model where co-factors are exchanged while CSL remains bound to DNA. Second, in Notch-ON conditions, there is a striking enrichment of Su(H) at *E(spl)-C*, its primary target locus, where its dwell time is significantly increased. These changes in CSL-binding dynamics, can enable a sensitive and accurate response to NICD at its target sites.

Here we find that NICD enhances both Su(H) recruitment and residence time at its target locus *E(spl)-C*, via a combination of mechanisms. One key step is that NICD-Su(H) complexes induce local changes in chromatin, which requires Trr (MLL3/4), a long-range co-activator that can contribute to chromatin opening (Herz et al., 2012). Notably, the consequence of NICD-induced chromatin opening is that it renders the target enhancers more accessible for additional complexes, regardless of whether they contain NICD or Hairless. Since binding of Hairless and NICD to Su(H) are mutually exclusive, it is likely that these represent discrete activator (Su(H)-NICD) and repressor (Su(H)-Hairless) complexes, although we have not formally shown Hairless recruitment relies on Su(H). This enhanced recruitment by NICD resembles that described for the glucocorticoid receptor (Voss et al., 2011) and other factors (Madsen et al., 2014),

(I and J) Live imaging of Su(H)WT in Notch-ON cells co-expressing control (I) or Trr (J) RNAi. Arrowheads indicate Su(H) recruitment in control.

(K) Percentage of nuclei with measurable Su(H) bands in the indicated genotypes. Three independent experiments, 12 nuclei scored in each experiment; box and whiskers min to max.

(L) Chromatin accessibility across the *E(spl)-C* locus in the conditions indicated, measured by enrichment of fragments to transposon tagging with ATAC, normalized to Neg1. n = 3; mean ± SEM.

(M) Model summarizing the changes in DNA binding of CSL complexes at target loci between Notch-OFF and Notch-ON nuclei that can lead to signaling amplification. CSL, blue; co-repressor, gray; NICD, green; Mam, pink.

See also Figure S7.

referred to as “assisted loading,” whereby the binding of one protein complex helps the binding of another. We propose that the localized chromatin remodeling brought about by Su(H)-NICD reduces obstacles (e.g., moves nucleosomes) to facilitate DNA binding, i.e., effectively increasing K_{ON} (Figure 7M). Such indirect cooperativity would render the response very sensitive to signal levels (Koshland et al., 1982; Sneppen et al., 2008; Zhang et al., 2013).

A second aspect helps explain how the transiently bound Su(H)-NICD complexes can successfully activate transcription. Although at genomic locations with paired binding motifs the dimerization of NICD could enhance binding (Arnett et al., 2010; Hass et al., 2015), our data argue that the presence of Mam itself confers a longer dwell time to the activator complex, most likely by favoring contacts with additional chromatin-associated factors, such as Mediator complex. One candidate to mediate these effects was CBP, a histone acetyltransferase that interacts with Mam and is necessary for its ability to stimulate transcription (Fryer et al., 2002; Wallberg et al., 2002). However, inhibiting CBP or depleting the Mediator subunit Med7 only slightly modified the Su(H) dynamics, suggesting that each makes at best a modest contribution to the change in its behavior. As neither manipulation fully replicated the effects of Mam inhibition/depletion, despite preventing transcriptional activation, it is likely that they also act at a later step in the initiation process. Thus Mam is likely to exert its early effects on Su(H) recruitment through a combination of other chromatin factors besides CBP. The interaction of the tripartite Su(H)-NICD-Mam complex with these chromatin factors, although still transient, could confer a probabilistic switch between an inactive state and an active state, by leaving a longer-lasting modification or reorganization of the chromatin template or initiation complex (Coulon et al., 2013; Lickwar et al., 2012).

The fact that the Su(H)-NICD activator complex also enhances recruitment of Hairless co-repressor complexes was entirely unexpected based on prevailing models, and has several important consequences. First, it will bring opposing enzymatic activities (e.g., both histone acetyl-transferases and histone deacetylases), which could create a covalent modification cycle with switch-like properties (Ferrell and Ha, 2014; Koshland et al., 1982; Sneppen et al., 2008), potentially further sensitizing responses to Notch. Second, enhanced recruitment of Hairless would ensure that genes are rapidly turned off after the signal decays, the switch operating in the converse direction when NICD levels decrease. Such “facilitated repression,” where transcriptional activators promote global chromatin decondensation to facilitate loading of repressors, has also been described during circadian gene regulation where it operates as an amplitude rheostat (Zhu et al., 2015).

In conclusion, our *in vivo* analysis of the mechanisms underlying the transcriptional response to Notch signaling reveal the fundamental importance of changes in DNA-binding dynamics and highlight how different mechanisms combine to enhance Su(H) recruitment and dwell time at *E(spl)-C* in Notch-ON cells. Whether both mechanisms operate at all Notch-regulated loci remains to be established, but they will likely be relevant for most genes where CSL occupancy was found to increase in Notch-ON conditions (Castel et al., 2013; Krejci and Bray, 2007; Wang et al., 2014). Furthermore, this new insight into Notch signaling leads us to propose that similar changes in the dynamics of nuclear effectors may also operate to deliver proper transcriptional outputs of other key signaling pathways.

STAR★METHODS

Detailed methods are provided in the online version of this paper and include the following:

- KEY RESOURCES TABLE
- CONTACT FOR REAGENT AND RESOURCE SHARING
- EXPERIMENTAL MODEL AND SUBJECT DETAILS
  - Experimental Animals
  - Fly Stocks
  - Generation of GFP Tagged Su(H) and Hairless Flies
  - Locus Tag
- METHOD DETAILS
  - Salivary Gland Cultures and Drug Treatments
  - Live Imaging and FRAP Setup
  - FRAP Modeling
  - PALM Imaging Using Su(H)-mEOS and SMT Analysis
  - Immunostainings
  - Immuno-Precipitation and Western Blots
  - mRNA Extraction and Quantitative RT-PCR
  - Measuring Chromatin Accessibility by ATAC
  - Isothermal Titration Calorimetry Measurements
  - Band Assay
  - Enrichment of Marks at the *E(spl)* Locus
  - Statistical Analyses

SUPPLEMENTAL INFORMATION

Supplemental Information includes seven figures, five tables, and two movies and can be found with this article online at https://doi.org/10.1016/j.devcel.2018.01.020.

ACKNOWLEDGMENTS

We thank members of the Bray Lab and of the Notch community for helpful discussions and Bill Harris for comments on the manuscript. This work was supported by grants from the Medical Research Council to S.J.B. (MR/L007177/1) and to K.H. (MR/K015850/1), from Wellcome Trust (L.M.; 099130/Z/12/Z) to CAIC, from NIH to R.K. (CA178974), from ANR (ChronoNet) and CNRS to F.P. and P.V., and by studentships from the Wellcome Trust for J.F.-S. and M.J. (099744/Z/12/Z), from EPSRC for S.A.R. (EP/L015455/1), and from BBSRC for Z.P. (BB/J014540/1). S.B. was an Amgen Scholar.

AUTHOR CONTRIBUTIONS

M.J.G.-L., J.F.-S., R.S., K.O., R.K., and S.J.B. designed the experiments. M.J.G.-L., J.F.-S., S.A.R., Z.P., S.B., and Z.Y. performed the experiments. M.J.G.-L., J.F.-S., R.S., S.A.R., L.M., K.O., R.K., Z.P., and S.J.B. analyzed the data. G.C.-M., M.L.J., P.V., K.B., and F.P. generated essential reagents. M.J.G.-L., J.F.-S., R.S., F.P., R.K., and S.J.B. wrote the manuscript.

DECLARATION OF INTERESTS

The authors declare no competing interests.

Received: June 2, 2017
Revised: November 27, 2017
Accepted: January 23, 2018
Published: February 22, 2018

REFERENCES

Aldaz, S., Escudero, L.M., and Freeman, M. (2010). Live imaging of *Drosophila* imaginal disc development. Proc. Natl. Acad. Sci. USA *107*, 14217–14222.

Arnett, K.L., Hass, M., McArthur, D.G., Ilagan, M.X.G., Aster, J.C., Kopan, R., and Blacklow, S.C. (2010). Structural and mechanistic insights into cooperative assembly of dimeric Notch transcription complexes. Nat. Struct. Mol. Biol. *17*, 1312–1317.

Barolo, S., Stone, T., Bang, A.G., and Posakony, J.W. (2002). Default repression and Notch signaling: hairless acts as an adaptor to recruit the corepressors Groucho and dCtBP to Suppressor of Hairless. Genes Dev. *16*, 1964–1976.

Beaudouin, J., Mora-Bermúdez, F., Klee, T., Daigle, N., and Ellenberg, J. (2006). Dissecting the contribution of diffusion and interactions to the mobility of nuclear proteins. Biophys. J. *90*, 1878–1894.

Boija, A., Mahat, D.B., Zare, A., Holmqvist, P.-H., Philip, P., Meyers, D.J., Cole, P.A., Lis, J.T., Stenberg, P., and Mannervik, M. (2017). CBP regulates recruitment and release of promoter-proximal RNA polymerase II. Mol. Cell *68*, 491–503.e5.

Borggrefe, T., and Oswald, F. (2009). The Notch signaling pathway: transcriptional regulation at Notch target genes. Cell. Mol. Life Sci. *66*, 1631–1646.

Bray, S.J. (2016). Notch signalling in context. Nat. Rev. Mol. Cell Biol. *17*, 722–735.

Bray, S.J. (2006). Notch signalling: a simple pathway becomes complex. Nat. Rev. Mol. Cell Biol. *7*, 678–689.

Buenrostro, J.D., Wu, B., Chang, H.Y., and Greenleaf, W.J. (2015). ATAC-seq: a method for assaying chromatin accessibility genome-wide. Curr. Protoc. Mol. Biol. *109*, 21.29.1–21.29.9.

Castel, D., Mourikis, P., Bartels, S.J., Brinkman, A.B., Tajbakhsh, S., and Stunnenberg, H.G. (2013). Dynamic binding of RBPJ is determined by Notch signaling status. Genes Dev. *27*, 1059–1071.

Chanet, S., Vodovar, N., Mayau, V., and Schweisguth, F. (2009). Genome engineering-based analysis of bearded family genes reveals both functional redundancy and a nonessential function in lateral inhibition in *Drosophila*. Genetics *182*, 1101–1108.

Chenouard, N., Bloch, I., and Olivo-Marin, J. (2013). Multiple hypothesis tracking for cluttered biological image sequences. IEEE Trans. Pattern Anal. Mach. Intell. *35*, 2736–3750.

Collins, K.J., Yuan, Z., and Kovall, R.A. (2014). Structure and function of the CSL-KyoT2 corepressor complex: a negative regulator of notch signaling. Structure *22*, 70–81.

Contreras, A.N., Yuan, Z., and Kovall, R.A. (2015). Thermodynamic binding analysis of Notch transcription complexes from *Drosophila melanogaster*. Protein Sci. *24*, 812–822.

Coulon, A., Chow, C.C., Singer, R.H., and Larson, D.R. (2013). Eukaryotic transcriptional dynamics: from single molecules to cell populations. Nat. Rev. Genet. *14*, 572–584.

Dallas, M.H., Varnum-Finney, B., Delaney, C., Kato, K., and Bernstein, I.D. (2005). Density of the Notch ligand Delta1 determines generation of B and T cell precursors from hematopoietic stem cells. J. Exp. Med. *201*, 1361–1366.

Del Bianco, C., Vedenko, A., Choi, S.H., Berger, M.F., Shokri, L., Bulyk, M.L., and Blacklow, S.C. (2010). Notch and MAML-1 complexation do not detectably alter the DNA binding specificity of the transcription factor CSL. PLoS One *5*, e15034.

Delaney, C. (2005). Dose-dependent effects of the Notch ligand Delta1 on ex vivo differentiation and in vivo marrow repopulating ability of cord blood cells. Blood *106*, 2693–2699.

Dorighi, K.M., Swigut, T., Henriques, T., Bhanu, N.V., Scruggs, B.S., Nady, N., Still, C.D., Garcia, B.A., Adelman, K., and Wysocka, J. (2017). Mll3 and Mll4 facilitate enhancer RNA synthesis and transcription from promoters independently of H3K4 monomethylation. Mol. Cell *66*, 568–576.e4.

Etheridge, T.J., Boulineau, R.L., Herbert, A., Watson, A.T., Daigaku, Y., Tucker, J., George, S., Jonsson, P., Palayret, M., Lando, D., et al. (2014). Quantification of DNA-associated proteins inside eukaryotic cells using single-molecule localization microscopy. Nucleic Acids Res. *42*, e146.

Ferrell, J.E., Jr., and Ha, S.H. (2014). Ultrasensitivity part I: michaelian responses and zero-order ultrasensitivity. Trends Biochem. Sci. *39*, 496–503.

Fortini, M.E., Rebay, I., Caron, L.A., and Artavanis-Tsakonas, S. (1993). An activated Notch receptor blocks cell-fate commitment in the developing *Drosophila* eye. Nature *365*, 555–557.

Fryer, C.J., Lamar, E., Turbachova, I., Kintner, C., and Jones, K.A. (2002). Mastermind mediates chromatin-specific transcription and turnover of the Notch enhancer complex. Genes Dev. *16*, 1397–1411.

Guentchev, M., and McKay, R.D.G. (2006). Notch controls proliferation and differentiation of stem cells in a dose-dependent manner. Eur. J. Neurosci. *23*, 2289–2296.

Hass, M.R., Liow, H.-H., Chen, X., Sharma, A., Inoue, Y.U., Inoue, T., Reeb, A., Martens, A., Fulbright, M., Raju, S., et al. (2015). SpDamID: marking DNA bound by protein complexes identifies notch-dimer responsive enhancers. Mol. Cell *59*, 685–697.

Helms, W., Lee, H., Ammerman, M., Parks, A.L., Muskavitch, M.A., and Yedvobnick, B. (1999). Engineered truncations in the *Drosophila* mastermind protein disrupt Notch pathway function. Dev. Biol. *215*, 358–374.

Herz, H.M., Mohan, M., Garruss, A.S., Liang, K., Takahashi, Y.H., Mickey, K., Voets, O., Verrijzer, C.P., and Shilatifard, A. (2012). Enhancer-associated H3K4 monomethylation by Trithorax-related, the *Drosophila* homolog of mammalian Mll3/Mll4. Genes Dev. *26*, 2604–2620.

Housden, B.E., Millen, K., and Bray, S.J. (2012). *Drosophila* reporter vectors compatible with ΦC31 integrase transgenesis techniques and their use to generate new notch reporter fly lines. G3 (Bethesda) *2*, 79–82.

Izeddin, I., Récamier, V., Bosanac, L., Cissé, I.I., Boudarene, L., Dugast-Darzacq, C., Proux, F., Bénichou, O., Voituriez, R., Bensaude, O., et al. (2014). Single-molecule tracking in live cells reveals distinct target-search strategies of transcription factors in the nucleus. Elife *3*, https://doi.org/10.7554/eLife.02230.

Kopan, R., and Ilagan, M.X. (2009). The canonical Notch signaling pathway: unfolding the activation mechanism. Cell *137*, 216–233.

Koshland, D.E., Jr., Goldbeter, A., and Stock, J.B. (1982). Amplification and adaptation in regulatory and sensory systems. Science *217*, 220–225.

Krebs, A.R., Imanci, D., Hoerner, L., Gaidatzis, D., Burger, L., and Schübeler, D. (2017). Genome-wide single-molecule footprinting reveals high RNA polymerase II turnover at paused promoters. Mol. Cell *67*, 411–422.e4.

Krejci, A., and Bray, S. (2007). Notch activation stimulates transient and selective binding of Su(H)/CSL to target enhancers. Genes Dev. *21*, 1322–1327.

Kulic, I., Robertson, G., Chang, L., Baker, J.H.E., Lockwood, W.W., Mok, W., Fuller, M., Fournier, M., Wong, N., Chou, V., et al. (2015). Loss of the Notch effector RBPJ promotes tumorigenesis. J. Exp. Med. *212*, 37–52.

Lickwar, C.R., Mueller, F., Hanlon, S.E., McNally, J.G., and Lieb, J.D. (2012). Genome-wide protein–DNA binding dynamics suggest a molecular clutch for transcription factor function. Nature *484*, 251–255.

Lis, J.T. (2007). Imaging *Drosophila* gene activation and polymerase pausing in vivo. Nature *450*, 198–202.

Lowe, N., Rees, J.S., Roote, J., Ryder, E., Armean, I.M., Johnson, G., Drummond, E., Spriggs, H., Drummond, J., Magbanua, J.P., et al.; The UK *Drosophila* Protein Trap Screening Consortium, Lilley, K.S., Russell, S., St Johnston, D. (2014). Analysis of the expression patterns, subcellular localisations and interaction partners of *Drosophila* proteins using a pigP protein trap library. Development *141*, 3994–4005.

Madsen, M.S., Siersbæk, R., Boergesen, M., Nielsen, R., and Mandrup, S. (2014). Peroxisome proliferator-activated receptor γ and C/EBPα synergistically activate key metabolic adipocyte genes by assisted loading. Mol. Cell. Biol. *34*, 939–954.

Maier, D., Nagel, A.C., Johannes, B., and Preiss, A. (1999). Subcellular localization of Hairless protein shows a major focus of activity within the nucleus. Mech. Dev. *89*, 195–199.

Mazzone, M., Selfors, L.M., Albeck, J., Overholtzer, M., Sale, S., Carroll, D.L., Pandya, D., Lu, Y., Mills, G.B., Aster, J.C., et al. (2010). Dose-dependent

induction of distinct phenotypic responses to Notch pathway activation in mammary epithelial cells. Proc. Natl. Acad. Sci. USA *107*, 5012–5017.

Morel, V., Lecourtois, M., Massiani, O., Maier, D., and Preiss, A. (2001). Transcriptional repression by suppressor of hairless involves the binding of a hairless-dCtBP complex in *Drosophila*. Curr. Biol. *11*, 789–792.

Morel, V., and Schweisguth, F. (2000). Repression by suppressor of hairless and activation by Notch are required to define a single row of single-minded expressing cells in the *Drosophila* embryo. Genes Dev. *14*, 377–388.

Mueller, F., Wach, P., and McNally, J.G. (2008). Evidence for a common mode of transcription factor interaction with chromatin as revealed by improved quantitative fluorescence recovery after photobleaching. Biophys. J. *94*, 3323–3339.

Nam, Y., Sliz, P., Song, L., Aster, J.C., and Blacklow, S.C. (2006). Structural basis for cooperativity in recruitment of MAML coactivators to notch transcription complexes. Cell *124*, 973–983.

Nam, Y., Weng, A.P., Aster, J.C., and Blacklow, S.C. (2003). Structural requirements for assembly of the CSL.intracellular Notch1.Mastermind-like 1 transcriptional activation complex. J. Biol. Chem. *278*, 21232–21239.

Nowell, C.S., and Radtke, F. (2017). Notch as a tumour suppressor. Nat. Rev. Cancer *17*, 145–159.

Ntziachristos, P., Lim, J.S., Sage, J., and Aifantis, I. (2014). From fly wings to targeted cancer therapies: a centennial for notch signaling. Cancer Cell *25*, 318–334.

Olivo-Marin, J.C. (2002). Extraction of spots in biological images using multiscale products. Pattern Recognit. *35*, 1989–1996.

Oswald, F., Rodriguez, P., Giaimo, B.D., Antonello, Z.A., Mira, L., Mittler, G., Thiel, V.N., Collins, K.J., Tabaja, N., Cizelsky, W., et al. (2016). A phospho-dependent mechanism involving NCoR and KMT2D controls a permissive chromatin state at Notch target genes. Nucleic Acids Res. *44*, 4703–4720.

Persson, F., Lindén, M., Unoson, C., and Elf, J. (2013). Extracting intracellular diffusive states and transition rates from single-molecule tracking data. Nat. Methods *10*, 265–269.

Phair, R.D., Gorski, S.A., and Misteli, T. (2004). Measurement of dynamic protein binding to chromatin in vivo, using photobleaching microscopy. Methods Enzymol. *375*, 393–414.

Rebay, I., Fehon, R.G., and Artavanis-Tsakonas, S. (1993). Specific truncations of *Drosophila* Notch define dominant activated and dominant negative forms of the receptor. Cell *74*, 319–329.

Saad, H., Gallardo, F., Dalvai, M., Tanguy-le-Gac, N., Lane, D., and Bystricky, K. (2014). DNA dynamics during early double-strand break processing revealed by non-intrusive imaging of living cells. PLoS Genet. *10*, e1004187.

Schneider, C.A., Rasband, W.S., and Eliceiri, K.W. (2012). NIH Image to ImageJ: 25 years of image analysis. Nat. Methods *9*, 671–675.

Schuettengruber, B., Bourbon, H.M., Di Croce, L., and Cavalli, G. (2017). Genome regulation by Polycomb and Trithorax: 70 years and counting. Cell *171*, 34–57.

Skalska, L., Stojnic, R., Li, J., Fischer, B., Cerda-Moya, G., Sakai, H., Tajbakhsh, S., Russell, S., Adryan, B., and Bray, S.J. (2015). Chromatin signatures at Notch-regulated enhancers reveal large-scale changes in H3K56ac upon activation. EMBO J. *34*, 1889–1904.

Sneppen, K., Micheelsen, M.A., and Dodd, I.B. (2008). Ultrasensitive gene regulation by positive feedback loops in nucleosome modification. Mol. Syst. Biol. *4*, 182.

van Royen, M.E., Farla, P., Mattern, K.A., Geverts, B., Trapman, J., and Houtsmuller, A.B. (2009). Fluorescence recovery after photobleaching (FRAP) to study nuclear protein dynamics in living cells. Methods Mol. Biol. *464*, 363–385.

VanderWielen, B.D., Yuan, Z., Friedmann, D.R., and Kovall, R.A. (2011). Transcriptional repression in the notch pathway. J. Biol. Chem. *286*, 14892–14902.

Voss, T.C., Schiltz, R.L., Sung, M.-H., Yen, P.M., Stamatoyannopoulos, J.A., Biddie, S.C., Johnson, T.A., Miranda, T.B., John, S., and Hager, G.L. (2011). Dynamic exchange at regulatory elements during chromatin remodeling underlies assisted loading mechanism. Cell *146*, 544–554.

Wallberg, A.E., Pedersen, K., Lendahl, U., and Roeder, R.G. (2002). p300 and PCAF act cooperatively to mediate transcriptional activation from chromatin templates by notch intracellular domains in vitro. Mol. Cell. Biol. *22*, 7812–7819.

Wang, H., Zang, C., Taing, L., Arnett, K.L., Wong, Y.J., Pear, W.S., Blacklow, S.C., Liu, X.S., and Aster, J.C. (2014). NOTCH1-RBPJ complexes drive target gene expression through dynamic interactions with superenhancers. Proc. Natl. Acad. Sci. USA *111*, 705–710.

Wilson, J.J., and Kovall, R.A. (2006). Crystal structure of the CSL-notch-mastermind ternary complex bound to DNA. Cell *124*, 985–996.

Yuan, Z., Praxenthaler, H., Tabaja, N., Torella, R., Preiss, A., Maier, D., and Kovall, R.A. (2016). Structure and function of the Su(H)-hairless repressor complex, the major antagonist of notch signaling in *Drosophila melanogaster*. PLoS Biol. *14*, e1002509.

Zhang, M., Chang, H., Zhang, Y., Yu, J., Wu, L., Ji, W., Chen, J., Liu, B., Lu, J., Liu, Y., et al. (2012). Rational design of true monomeric and bright photoactivatable fluorescent proteins. Nat. Methods *9*, 727–729.

Zhang, Q., Bhattacharya, S., and Andersen, M.E. (2013). Ultrasensitive response motifs: basic amplifiers in molecular signalling networks. Open Biol. *3*, 130031.

Zhu, B., Gates, L.A., Stashi, E., Dasgupta, S., Gonzales, N., Dean, A., Dacso, C.C., York, B., and O'Malley, B.W. (2015). Coactivator-dependent oscillation of chromatin accessibility dictates circadian gene amplitude via REV-ERB loading. Mol. Cell *60*, 769–783.

STAR★METHODS

KEY RESOURCES TABLE

REAGENT or RESOURCE	SOURCE	IDENTIFIER
Antibodies		
Rabbit anti-PolII	Abcam	Cat#5095; RRID: AB_304749
Rabbit anti-H3K27ac	Abcam	Cat# ab4729; RRID: AB_2118291
Rabbit anti-H3K4me1	Abcam	Cat# ab8895; RRID: AB_306847
Rabbit anti-Trr	(Herz et al., 2012)	N/A
Goat anti-Su(H)	Santa Cruz Biotechnology	Cat# sc-15813; RRID: AB_672840
Rabbit anti-Su(H)	Santa Cruz Biotechnology	Cat# sc-28713; RRID: AB_2179304
Rabbit anti-GFP	ThermoFisher Scientific	Cat# A-11122; RRID: AB_221569
Mouse anti-NICD	Developmental Studies Hybridoma Bank	Cat# c17.9c6; RRID: AB_528410
Rabbit anti-Hairless	(Maier et al., 1999)	N/A
Donkey anti-Rabbit FITC	Jackson ImmunoResearch Laboratories, Inc	Cat# 711-095-152; RRID: AB_2315776
Goat anti-Rabbit HRP	BioRad/ AbD Serotec	Cat# 170-6515; RRID: AB_11125142
Goat anti-Mouse HRP	BioRad/ AbD Serotec	Cat# 170-6516; RRID: AB_11125547
Chemicals, Peptides, and Recombinant Proteins		
Shield and Sang M3 insect medium	Sigma Aldrich	Cat#: S3652
Fetal Bovin Serum	Sigma Aldrich	Cat#: F9665
Antibiotic-antimicotic	ThermoFisher Scientific	Cat#: 15240062
Methyl-cellulose	Sigma Aldrich	Cat#: M0387
Triptolide	Sigma Aldrich	Cat#: T3652
C646	Sigma Aldrich	Cat#: SML0002
TRIzol	ThermoFisher Scientific	Cat#: 15596026
Oligo(dT)^{15} primers	Promega	Cat#: C1101
M-MLV reverse transcriptase	Promega	Cat#: M1701
NEBNext High-Fidelity 2x PCR Master Mix	New England Biolabs	Cat#: M0541S
SYBR green	ThermoFisher Scientific	Cat#: S7563
Hoechst 33258	SigmaAldrich	Cat#: 94403-1ML
Vectashield mounting medium	Vector laboratories	Cat# H-1000; RRID: AB_2336789
cOmplete™ Protease Inhibitor Cocktail	Roche (SigmaAldrich)	Cat#: 11697498001
G-agarose beads	Santa Cruz Biotechnology	Cat# sc-2002; RRID: AB_10200697
Amersham ECL Western Blotting Detection Reagent	GE Healthcare Life Sciences	Cat#: RPN2109
Glutathione-Sepharose column	GE Healthcare Life Sciences	Cat#: 17528201
PreScission Protease	GE Healthcare Life Sciences	Cat#: 27084301
Critical Commercial Assays		
Ambion's DNA-free DNA removal kit	ThermoFisher Scientific	Cat#: AM1906
LightCycler 480 SYBR Green I Master PCR kit	Roche	Cat#: 04707516001
Nexera DNA Library preparation kit	Illumina	Cat#: FC-121-1030
MinElute PCR purification kit	Qiagen	Cat#: 28004
DNeasy Blood and Tissue Kit	Qiagen	Cat#: 69504
Experimental Models: Organisms/Strains		
D. melanogaster 1151-Gal4	Laboratory of Lingadahalli S. Shashidhara	FBti0007229
D. melanogaster UAS-NDECD	(Fortini et al., 1993; Rebay et al., 1993)	N/A
D. melanogaster UAS-Hairless-RNAi	Bloomington Drosophila Stock Center	FBst0027315
D. melanogaster UAS-Trr-RNAi	Bloomington Drosophila Stock Center	FBst0036916

(*Continued on next page*)

Continued

REAGENT or RESOURCE	SOURCE	IDENTIFIER
D. melanogaster UAS-Mam-RNAi	Bloomington Drosophila Stock Center	FBst0028046
D. melanogaster UAS-MamDN	(Helms et al., 1999)	N/A
D. melanogaster NRE-GFP	(Housden et al., 2012)	N/A
D. melanogaster Dup E(spl)d-8	(Chanet et al., 2009)	N/A
D. melanogaster UAS-nlsGFP	Bloomington Drosophila Stock Center	FBst0065402
D. melanogaster Fkh::GFP	Bloomington Drosophila Stock Center	FBst0043951
D. melanogaster SMRTR::YFP	Kyoto Stock Center	FBst0324767
D. melanogaster yw	Bloomington Drosophila Stock Center	FBst0001495
D. melanogaster vas-phiC31;; 3xP3-RFP.attP-86Fb	Bloomington Drosophila Stock Center	FBst0024749
D. melanogaster vas-phiC31; 3xP3-RFP.attP-51D	Bloomington Drosophila Stock Center	FBst0024483
D. melanogaster $Su(H)^{AR9}$ Null allele	Bloomington Drosophila Stock Center	FBst0030477
D. melanogaster $Su(H)^{SF8}$ Null allele	Kyoto Stock Center	FBst0300297
D. melanogaster $Hairless^{P8}$ Null allele	(Maier et al., 1999)	N/A
D. melanogaster $Hairless^{1}$ Null allele	Bloomington Drosophila Stock Center	FBst0000515
D. melanogaster UAS-ParB1-mCherry	This paper	N/A
Oligonucleotides		
Oligonucleotides for mRNA levels measurement and ATAC qPCR	This paper	Table S5
Recombinant DNA		
pHD-DsRed	Addgene	Cat#: 51434
Software and Algorithms		
FRAP parameter estimation code	Github	https://github.com/rstojnic/suh_frap
ImageJ v1.48c	(Schneider et al., 2012)	N/A
GraphPad Prism 7	GraphPad Software, Inc.	N/A
Origin Software	OriginLab	N/A

CONTACT FOR REAGENT AND RESOURCE SHARING

Further information and requests for resources and reagents should be directed to and will be fulfilled by the Lead Contact, Sarah J. Bray (sjb32@cam.ac.uk).

EXPERIMENTAL MODEL AND SUBJECT DETAILS

Experimental Animals

Species: *Drosophila melanogaster*. Flies were grown and maintained on food consisting of the following ingredients: Glucose 76g/l, Cornmeal flour 69g/l, Yeast 15g/l, Agar 4.5g/l, Methylparaben 2.5ml/l

Animals of both sexes were used for this study.

Fly Stocks

For genetic manipulations the Gal4 driver line 1151-Gal4 (Lingadahalli S. Shashidhara, Centre for Cellular and Molecular Biology, Hyderabad, India) was used and combined with UAS-$N^{\Delta ECD}$ to provide constitutively active Notch (Fortini et al., 1993; Rebay et al., 1993). These were combined with RNAi lines as listed in Table S4 and STAR Methods key resources table, including UAS-Hairless-RNAi (Bloomington Drosophila Stock Center, BL-27315), UAS-Mam-RNAi (BL 28046), UAS-Trr-RNAi (BL36916) or with UAS-MamDN to block Mam activity (Helms et al., 1999). Crosses were maintained at 25°C. Other lines used include NRE-GFP (Housden et al., 2012), DpE(spl)∂-8 (Chanet et al., 2009), UAS-nls-GFP (Bloomington 65402), Fkh::GFP (Bloomington 43951), SMRTR::YFP (DGRC 115513) (Lowe et al., 2014) and yw (Bloomington 1495). Further details are provided in Table S4.

Generation of GFP Tagged Su(H) and Hairless Flies

To generate a genomic *Su(H)::GFP* construct, AttB plasmids containing the genomic region 2L:15038840-15045039 were constructed, with the coding sequences of eGFP (eGFP CloneTech; here referred to as Su(H)::GFP for simplicity) inserted at position

446 of the Su(H) transcript, (end of 5' UTR, 2L-15039933), to generate a protein fusion at the N-terminus. This plasmid was injected into a strain containing phiC31 integrase and AttP site in position 86F8 in chromosome 3 (Bloomington 24749) to generate transgenic *Su(H)::GFP* flies. Identical strategy was used to produce Su(H)::mEOS3.2 (here referred to as Su(H)::mEOS for simplicity) (M. Zhang et al., 2012). The functionality of the tagged proteins was assessed by their ability to rescue $Su(H)^{AR9}/Su(H)^{SF8}$ flies; the viability of the mutant flies was fully rescued indicating that the plasmid confers functional Su(H) activity. Using site-directed mutagenesis, we generated two mutated versions of Su(H)::GFP, one with impaired DNA binding, $Su(H)^{R266H}$ (R266H, G to A substitution in position 413 in exon 3) and another with impaired binding to NICD, $Su(H)^{NBM}$ (F309A, TT to GC in position 541 in exon 3, V311A, T to C in position 548 in exon 3, E446R, GA to CG in position 71 in exon 4, R470E, CGC to GAG in position 143 in exon 4 and E473R, GA to CG in position 152 in exon 4). Transgenic flies were then generated in the same way as for $Su(H)^{WT}$. All of the tagged Su(H) proteins were expressed at similar level to the endogenous (Figure S1)

The genomic *Hairless::GFP* AttB plasmids contained the genomic region 3R 20621141- 20628985 with the coding sequences of eGFP (eGFP CloneTech) inserted at position 5648, to generate a protein fusion at the C-terminus. This plasmid was injected into a strain containing phiC31 integrase and AttP site in position 51D in chromosome 2 (Bloomington stock 24483) to generate transgenic *Hairless::GFP* flies. The functionality of the protein was assessed by its ability to rescue Hairless mutants; the transgene fully rescued the viability of H^{P8}/H^{1} mutant flies to adulthood and the phenotypes of homozygous H^{P8} mutant clones in the wing disc indicating that the plasmid confers functional Hairless activity. Levels of expression were also similar to endogenous (Figure S1). We note however that the rescued H^{P8}/H^{1} adult flies were not fully fertile, most likely because the genomic fragment lacked regulatory sequences required for germ-line expression.

Locus Tag

Int1 sequences were inserted into an intergenic region in *E(spl)-C* via a two-step method using CRISPR/Cas9 genome editing and PhiC31 integrase mediated transgenesis. An AttP site was first integrated via Cas9/CRISPR mediated homology directed repair (HDR) using a single guide RNA (Sequence: AGAACCCTCAAGATTTGTAA, Chromosome 3R 26038865:26038884) and a template plasmid with ~1kb homology directly adjacent to the guideRNA cut site on both sides and a fluorescent marker (pHD-DsRed, AddGene). Int1 sequences were then introduced by standard phiC1 mediated integration and recovered using the mini-white marker. HDR injections used a molar ratio of 1 guide RNA: 2 Homology Directed Repair template and all injections used a final concentration of 1μg/μl. attP and Int1 insertions were genotyped by PCR. Strains containing UAS-ParB1-mcherry inserted into AttP.86Fb were generated by standard phiC1 mediated integration. Further details available on request.

METHOD DETAILS

Salivary Gland Cultures and Drug Treatments

Salivary glands of early third-instar larvae were dissected in dissecting media [Shields and Sang M3 insect medium (Sigma, S3652), supplemented with 5% FBS (Sigma, F9665) and 1× Antibiotic-Antimycotic (Gibco, 15240-062)]. Unharmed gland pairs were placed in a Poly-L-lysine treated observation chamber (Aldaz et al., 2010). The chamber was made with a double layer of double side tape (Sellotape acid free perforated using a hole puncher of 9-mm diameter hole). The tape was attached to a 22 × 50-mm coverslip. The coverslip was then attached to a metal slide with a cut-out panel. Finally, the chamber/hole was filled with medium and the discs placed in it and then covered with the semipermeable membrane and covered with viscous media [dissecting media + 2.5× wt/vol methyl-cellulose (Sigma-Aldrich)]. For PALM imaging of fixed samples, salivary glands were fixed in 4% Form-aldehyde for 15min, washed 3x 15min in PBT (PSB+ 0.3% Triton X-100) then left in PBT at 4°C overnight before mounting as for live samples.

For the inhibitor treatments, salivary glands of early third-instar larvae were dissected in dissecting media and incubated for 1h in the presence of DMSO (10 μl), Triptolide (10μM) or C646 (30μM). If salivary glands were to be used for imaging, were mounted in an observation chamber in viscous media containing the inhibitors or DMSO. For mRNA extraction, salivary glands were immediately transfer to TriZOL and proceed as explained below.

Live Imaging and FRAP Setup

Image acquisition was performed using a Leica TCS SP8 confocal microscope equipped with 40×/1.30 NA oil and 60X/1.40 NA oil HC PL APO CS2 objective lens. For live imaging of Su(H)::GFP and Hairless::GFP nuclei the pinhole was set to 3-Airy and Z-stacks spaced 0.5μm were taken to cover the volume of the nucleus using 512x512 resolution and scanning speed 600Hz. Two or four frame averages were used to increase the signal-to-noise ratio as indicated. >20 nuclei were imaged for each genotype, unless indicated, and were usually taken from 3 or more salivary glands.

For FRAP experiments, the pinhole was set to 3-Airy and single plane images were obtained using a scanning speed of 1400Hz. 10 images were acquired at 0.098s intervals before bleaching and then point-bleaching, directed to a single point in the nucleus, was performed for 1s, using 100% 488-nm laser power. These conditions were selected after different point bleaching conditions were tested to identify those that produced bleaching through the whole nucleus, yielding a column that had a similar depth of bleaching throughout (Figure S2). 300 images were then acquired at 0.098s intervals after bleaching followed by a further 100 images at 1s intervals. For the band FRAP experiments, 300 post-bleaching images were acquired at 0.115s intervals followed by 100 images at 2s intervals. In Figure 3A, half of the nucleus was bleached rather than a point. For each experiment, we measured fluorescence

intensities in total nucleus (T), bleached (B), unbleached (UB) and background (BG) regions over time using ImageJ. >15 nuclei were imaged for each genotype and were usually taken from 3 or more salivary glands.

To obtain the recovery curves, we perform a double normalization, where the signal in B is normalized to the average prebleach signal and, at the same time, considers the loss of total signal due to the bleach pulse and bleaching during post-bleach imaging. For that, we use the formula:

$$(T_{pre}\text{-}BG) \times (B_t\text{-}BG) / (T_t\text{-}BG) \times (B_{pre}\text{-}BG)$$

where BG=Is the mean BG along the experiment, T_{pre} is the mean fluorescence in T before bleaching, B_{pre} is the mean intensity in B before bleaching, T_t is the fluorescence in T over time and B_t is the fluorescence in B over time (Phair et al., 2004).

FRAP Modeling

The fluorescent recovery after photobleaching (FRAP) was modelled using the reaction-diffusion model, following the approach of (Beaudouin et al., 2006). The partial differential equations describing the movement of molecules were simulated using the finite difference approach. The simulation was performed separately for each nucleus replicate by initialising it with the real nucleus shape and bleaching profile derived by comparing the pre- and post-bleach images. To reduce the noise and speed up the simulations, we smoothed the images using a 5-pixel Gaussian kernel and then reduced the resolution of the images by averaging over 10x10 pixels. We also simulated higher resolution nuclei by averaging over 5x5 pixels, but this did not alter the results significantly (Figure S2). It is known that the initialisation step has a large influence on the accuracy of parameter estimation (Mueller et al., 2008). To reduce the noise in the initialisation, we further averaged the first three post-bleach images to create a single image which we used to initialise the simulations using the approach from (Beaudouin et al., 2006). We also scaled the total fluorescence within the bleached area by a number close to 1 so that the binned fluorescence within the binned bleached area exactly match the fluorescence within the original (full-resolution) bleach area. We monitored the recovery at two distinct places in the nucleus: at the circular bleaching location, and at a circular location at the opposite end of the nucleus. To improve the precision of parameter estimation we simultaneously fitted the recovery at both locations (van Royen et al., 2009).

We fitted the FRAP model where the behaviour of the protein is described by a reaction-diffusion reaction which a diffusion constant D, rate of binding to DNA k_{on} and unbinding from DNA k_{off}. Following the approach of (Beaudouin et al., 2006) we simulated the system using the finite difference approach where we tile the imaged 2D plane into discrete tiles (bins) and simulate their dynamics. We denote the free molecules along the spatial steps i and j at time t as $F(i, j, t)$ and the molecules bound to DNA as $C(i, j, t)$. This leads to the following equations:

$$\frac{\partial F(i,j,t)}{\partial t} = \frac{D}{p_i^2}(F(i-1,j,t)+F(i+1,j,t)-2F(i,j,t)) + \frac{D}{p_j^2}(F(i,j-1,t)+F(i,j+1,t)-2F(i,j,t)) - k_1(i,j)F(i,j,t) + k_{off}C(i,j,t)$$

$$\frac{\partial C(i,j,t)}{\partial t} = k_1(i,j)F(i,j,t) - k_{off}C(i,j,t)$$

The rate k_1 (i, j) depends on k_{on} and an unknown proportionality coefficient A that can be estimated from the steady-state image following the approach of Beaudouin et al. (2006, Appendix A) (written here in the matrix form):

$$k_1 = \frac{k_{off}}{Free}\left(\frac{i^{st}}{i^{st}_{avg}} - Free\right)$$

where *Free* is the average number of free molecules in the nucleus, i^{st} is the matrix of fluorescence intensities over the spatial grid, and i^{st}_{avg} is its average value. This equation assumes that the nucleus is in equilibrium and that therefore the fluorescence can be expressed as a sum of uniformly distributed free molecules plus non-uniformly distributed molecules bound to DNA.

In the situation when there is no binding, the equation simplifies considerably by setting $C(i, j, t) = 0$, which allows us to directly infer the distribution of free molecules before and after bleaching by looking at the fluorescence intensity.

We simulated recovery for a grid of parameter values to find the parameter combination that explains the data the best. To summarise the results over the replicates, we summed up the mean square errors (comparing observed and predicted recovery) for each of the replicates. We highlight the parameter combinations that have the lowest global error rate, i.e. whose deviation from the observed recovery is on average, over the whole recovery curve, within 1% of the best model. This is equivalent to the maximum likelihood estimate of the parameters assuming normal errors.

The code for the FRAP parameter estimation is available on github: https://github.com/rstojnic/suh_frap.

PALM Imaging Using Su(H)-mEOS and SMT Analysis

Cultured salivary gland cells were imaged using an inverted microscope frame customised for localisation microscopy. The output beams of a laser combiner (Omicron LightHUB) were collimated to a 12mm diameter using a reflective collimator and then circularly polarised using an achromatic quarter wave plate. The excitation (561nm) and activation (405nm) beams were then demagnified onto the sample through a 250mm tube lens and 60x silicone oil immersion objective (Olympus UPLSAPO60XS2, 1.3 NA). This resulted in

an illumination area at the sample plane with a diameter of 96 μm. Fluorescence was collected through the same objective lens, filtered (Chroma-ZT405/488/561/640rpc) to remove scattered and reflected excitation or activation light before being focused onto a sCMOS camera (Hamamatsu, Flash 4.0). A final band-pass filter (Semrock-FF01 600/52-25) was placed before the camera to ensure high attenuation of any remaining laser light.

For each experiment, we imaged a 50×50 μm region, sufficient to observe one nucleus, with continuous excitation beam (150-250 W/cm2) and regular bursts of activation beam (3-9 W/cm2). 5-8 cells were imaged at exposure times of 10, 50, 100, 200 and 500 ms.

Images were analysed using undecimated wavelet transform for detection of fluorescent proteins. Subsequent tracking of these diffusing protein molecules was carried out using multiple hypothesis tracking algorithm. For a conservative approach, tracks with detections in each consecutive frame were included in the analysis i.e. no detection gaps were allowed inside a track. Detection and tracking analysis was performed using icy-plugins based on (Chenouard et al., 2013; Olivo-Marin, 2002). Different diffusive states, their dwell times and transition probabilities were computed using variational Bayesian treatment of Hidden Markov models based on (Persson et al., 2013). The analysis was performed on tracks longer than three time points.

To benchmark the vbSPT analysis software in the context of our biological data, we simulated trajectories of random walk with four diffusion states, in a discretised cell geometry (with 15 nm resolution) as discussed in (Persson et al., 2013). Given the simulated single molecule positions we generated images by assuming a 2D Gaussian point spread function (σ = 120 nm). Poisson and Gaussian noise (σ = 1.1 e-, matching the readout noise of the camera used in our experiments) was subsequently added to the images. A representative experimental and simulated image is shown in Figure S3. Spot detection, tracking and analysis were performed using the procedure discussed above and results from ten data sets are shown in Figure S3. The vbSPT software successfully differentiated between different diffusion states for all the data sets, albeit yielding very slight variations in the diffusion coefficients due to the added noise (for D1-D3) and the confinement induced by cell boundaries (D4). For a more detailed discussion on the performance of vbSPT under different conditions see (Persson et al., 2013).

We used fixed samples to estimate the localization errors under the experimental conditions. The covariance of the computed localisations of the same fixed molecule over time gives a measure of the precision for our imaging system of approximately 55 nm (with the Spot Tracking plug-in of icy) and 33nm (with a custom written localisation software), within the range estimated in other SMT studies (e.g. 70nm, Izeddin et al., 2014). The average jump distance for a molecule with slowest diffusion coefficient in our experiments was D=0.08um^2/sec) suggesting that signal to noise ratio puts a limit on the slowest diffusion coefficient that can be measured from the images. We note that the estimated peak signal to noise ratio (PSNR) of the fixed sample images is ~19dB, which is lower than for the unfixed samples where the estimated PSNR is ~29 dB. This suggests that the localisation precision for the tracked molecules is better than 55nm. The PSNR of simulated images is ~32.8 dB.

Immunostainings

Salivary glands or wing discs of early third-instar larvae were dissected in PBS and fixed in 4% formaldehyde for 15 min. Glands were washed in PBS-T (0.3% Triton X-100 in PBS) and blocked in BBT (PBS-T + 1% BSA) before incubation with primary antibody overnight at 4°C. These included Rb anti-PolII (1:500, Abcam 5095), Rb anti-H3K27ac (1:500, Abcam 4729), Rb anti-H3K4me1 (1:1000, Abcam 8895), Rb anti-Trr (1:100, Herz et al., 2012). After several washes in PBS-T, glands were incubated with FITC secondary antibody, (Jackson ImmunoResearch Laboratories, Inc), for 2–4h at Room Temperature (RT) followed by washing in PBS-T and labelling with Hoechst 33258 (Sigma). Samples were mounted in Vectashield (Vector Laboratories) for imaging.

Immuno-Precipitation and Western Blots

Extracts were prepared by lysing and homogenizing 20-40 third-instar larval heads in 100μl IP buffer (50mM Tris-HCL pH8, 150mM NaCl, 10% Glycerol, 0.5% Triton X-100, and proteinase inhibitor cocktail). After 30min on ice, debris was pelleted by centrifugation (13,000 rpm, 4°C for 15min) and 220μg of protein extract was diluted to final volume of 500μl in IP buffer then incubated with Goat anti-Su(H) (1:100, Santa Cruz Tech sc-15813) or Rabbit anti-GFP (1:1.000, Invitrogen A11122) overnight at 4°C. 30μl protein G-agarose beads were added and samples incubated for 3h at 4°C before the beads were pelleted, washed 5x in IP buffer, and resuspended in SDS-sample buffer (130mM Tris-Cl, pH8, 20% Glycerol, 4% SDS, 0.02 Bromophenol blue, 2% β-mercaptoethanol). Samples were analyzed on 10% acrylamide SDS-PAGE gels (Bio-Rad) before transfer to Nitrocellulose membrane. Membranes were blocked in TBTM (TBS, + 0.05% tween, 3% milk) for 1h and incubated overnight at 4°C in primary antibodies (Rb anti-GFP 1:500, Invitrogen A11122; Rb anti-Su(H) 1:400 Santa Cruz sc-28713; Mo anti-NICD 1:100, Developmental Studies Hybridoma Bank C17.9C6; Rb anti-Hairless 1:1000, Maier et al., 1999). Following 3x 15min washes at RT (TBS + 0.05 Tween) membranes were incubated 1h with secondary antibodies (1:2000, HRP conjugated Goat anti-Mouse and Goat anti-Rabbit, BioRad) then washed 4x 15min in TBT. For detection, Amersham ECL Western Blotting Detection Reagent (GE Healthcare Life Sciences) was used.

mRNA Extraction and Quantitative RT-PCR

RNA from 20 dissected third instar salivary gland pairs per condition was extracted. Glands were dissected in dissecting media (Shields and Sang M3 insect medium + 5% FBS + 1× Antibiotic-Antimycotic), quickly washed in PBS and incubated in TriZOL for 10min. Then, we added chloroform and incubated for another 10min before spining for 10min at 4°C. The supernatant containing RNA was precipitated in isopropanol overnight at -20°C. After a wash with Ethanol, the pellet was resuspended in DEPC-treated water. Samples were treated with Ambion's DNA-free kit (#AM1906) to eliminate genomic DNA, following manufacturer instructions.

We synthesized cDNA using Oligo(dT)$_{15}$ Primers (Promega C1101) and M-MLV reverse transcriptase (Promega M531A). Quantitative PCR was then performed using LightCycler® 480 SYBR Green I Master PCR Kit (Roche 04707516001) with a Roche Light Cycler. Samples were normalized using the Rpl32 gene as control. Primers used were

mα (GCAGGAGGACGAGGAGGATG and GATCCTGGAATTGCATGGAG)
mß (GCTGGACTTGAAACCGC and AGAAGTGAGCAGCAGCC),
m3 (AGCCCACCCACCTCAAC and GTCTGCAGCTCCATTAGTC)
Rpl32 (ATGCTAAGCTGTCGCACAAATG and GTTCGATCCGTAACCGATGT).

Measuring Chromatin Accessibility by ATAC

ATAC was performed largely as described in (Buenrostro et al., 2015), that is: Ten pairs of salivary glands were dissected from wandering third instar larvae of each genotype in PBS. Glands were transferred in PBS to LoBind microcentrifuge tubes (Eppendorf) treated with 1% BSA to avoid sticking. Glands were washed again in PBS by pelleting at 500xg for one minute and resuspending in 1mL PBS. A two-step lysis was performed where fat and debris were removed in the first step and the salivary gland nuclei were released in the second: First, glands were pelleted and resuspended in 50μL lysis buffer (10mM Tris-HCl, pH7.4, 10mM NaCl, 3mM $MgCl_2$, 0.1% NP-40). After five minutes on ice, the liquid was pipetted up and down but glands always remained intact with fat cells coming unattached. Glands were pelleted again (fat remained floating) and resuspended in 50μL of the same buffer with the addition of 1μL of 10% NP-40 to bring the final concentration to 0.3%. After five minutes on ice and vigorous pipetting, the released nuclei were pelleted at 100xg for five minutes at 4°C. The nuclei were resuspended in 30μL TD buffer (Illumina #FC-121-1030) and 5μL was used to observe the nuclei under a microscope to check the lysis had worked.

The tagmentation reaction was performed by transferring the remaining 25μL of nuclei to PCR tubes containing TD buffer and mixing with 22.5μL nuclease-free water and 2.5μL of Tagment DNA enzyme (Illumina #FC-121-1030) by gentle pipetting. The reaction was incubated at 37°C for 30 minutes, after which the DNA was immediately purified using the Qiagen MinElute PCR purification kit and eluted in 10μL EB.

Custom Nextera PCR primers were used for PCR amplification of DNA (see below, Table S5). 10μL tagmented DNA was combined with 10μL water, 2.5μL of each primer at 25μM concentration, and 25μL NEBNext High-Fidelity 2x PCR Master Mix (NEB #M0541S). Nextera PCR primer 1 was always used with a different Nextera PCR primer 2 for each sample. Five PCR cycles were performed as follows: 72°C, 5 mins; 98°C, 30 secs; five cycles (98°C, 10 secs; 63°C, 30 secs; 72°C, 1 min). After five cycles, the reactions were kept on ice while qPCR using a Roche Lightcycler 480 II system was performed with a 5μL sample, in order to determine the number of additional cycles for each sample. To a single well of a 384 multiwell plate (Roche #04 729 749 001), 5μL of the PCR reaction was combined with 5μL NEBNext High-Fidelity 2x PCR Master Mix, 2μL SYBR green at 4.5x (diluted from 10,000x, Invitrogen #S7563) and 1.5μL of each primer at 4.2μM concentration. 20 qPCR cycles were performed and the software was used to determine how many additional cycles corresponded to the fluorescence reaching closest to one quarter of the maximum. This number of cycles was then performed with the remaining 45μL PCR reaction (always between six and nine additional cycles): 98°C, 30 secs; cycles (98°C, 10 secs; 63°C, 30 secs; 72°C, 1 min). Finally, the DNA was once again purified with the Qiagen MinElute PCR purification kit and eluted in 20μL EB.

The DNA was diluted five-fold before quantification by standard qPCR using SYBR green 2x master-mix (Roche #04707516001). Primers used are listed in Table S5 Fold enrichment of open chromatin was calculated by comparing Cp values obtained from tagmented DNA to those obtained from genomic DNA extracted from salivary glands using the Qiagen DNeasy Blood and Tissue Kit (Qiagen #69504), and by comparing all primer regions tested to the negative control closed chromatin region. In other words, 2 to the power of $[(Cp_{test,naked} - Cp_{test,ATAC}) - (Cp_{negative,naked} - Cp_{negative,ATAC})]$.

Nextera PCR Primers for DNA Amplification:
Nextera PCR primer 1 AATGATACGGCGACCACCGAGATCTACACTCGTCGGCAGCGTCAGATGTG
Nextera PCR primer 2.1 CAAGCAGAAGACGGCATACGAGATTCGCCTTAGTCTCGTGGGCTCGGAGATGT
Nextera PCR primer 2.2 CAAGCAGAAGACGGCATACGAGATCTAGTACGGTCTCGTGGGCTCGGAGATGT
Nextera PCR primer 2.3 CAAGCAGAAGACGGCATACGAGATTTCTGCCTGTCTCGTGGGCTCGGAGATGT
Nextera PCR primer 2.4 CAAGCAGAAGACGGCATACGAGATGCTCAGGAGTCTCGTGGGCTCGGAGATGT
Nextera PCR primer 2.5 CAAGCAGAAGACGGCATACGAGATAGGAGTCCGTCTCGTGGGCTCGGAGATGT
Nextera PCR primer 2.6 CAAGCAGAAGACGGCATACGAGATCATGCCTAGTCTCGTGGGCTCGGAGATGT
Nextera PCR primer 2.7 CAAGCAGAAGACGGCATACGAGATGTAGAGAGGTCTCGTGGGCTCGGAGATGT
Nextera PCR primer 2.8 CAAGCAGAAGACGGCATACGAGATCCTCTCTGGTCTCGTGGGCTCGGAGATGT
Nextera PCR primer 2.9 CAAGCAGAAGACGGCATACGAGATAGCGTAGCGTCTCGTGGGCTCGGAGATGT
Nextera PCR primer 2.10 CAAGCAGAAGACGGCATACGAGATCAGCCTCGGTCTCGTGGGCTCGGAGATGT
Nextera PCR primer 2.11 CAAGCAGAAGACGGCATACGAGATTGCCTCTTGTCTCGTGGGCTCGGAGATGT
Nextera PCR primer 2.12 CAAGCAGAAGACGGCATACGAGATTCCTCTACGTCTCGTGGGCTCGGAGATGT
Nextera PCR primer 2.13 CAAGCAGAAGACGGCATACGAGATATCACGACGTCTCGTGGGCTCGGAGATGT
Nextera PCR primer 2.14 CAAGCAGAAGACGGCATACGAGATACAGTGGTGTCTCGTGGGCTCGGAGATGT

Nextera PCR primer 2.15 CAAGCAGAAGACGGCATACGAGATCAGATCCAGTCTCGTGGGCTCGGAGATGT
Primers Used in qPCR Analysis:
Rab11 intron ACTGAAAATGGGCCGTTTCG AGGAGTGGTAATCGACGGTC
Eip78C EcR AGAAGTAGGGGCCGTCAAGT GTGTAAGACCCGTCGCATTT
Negative 1 GCATTTTTGTGGCAGAGGCA CTCTTTCGGTGTCGCCTTCT
Mst87F ATCCTTTGCCTCTTCAGTCC; AATAATGATACAAAATCTGGTTACGC
mβ gene AGAAGTGAGCAGCAGCCATC GCTGGACTTGAAACCGCACC
mβ peak AGAGGTCTGTGCGACTTGG GGATGGAAGGCATGTGCT
mα peak AAGCCAGTGGACTCTGCTCT TGATCTCCAAGCGGAGTATG
mα gene GCAGGAGGACGAGGAGGATG GATCCTGGAATTGCATGGAG
m3 peak ACACACACAAACACCCATCC CGAGGCAGTAGCCTATGTGA
m3 gene CGTCTGCAGCTCAATTAGTC AGCCCACCCACCTCAACCAG
m8 gene CAATTCCACGAAGCACAGTC GAGGAGCAGTCCATCGAGTT

Isothermal Titration Calorimetry Measurements

Recombinant Su(H) (98-523) and NICD (1762-2142) proteins were overexpressed and purified from bacteria as GST-fusion proteins. Bacteria were harvested by centrifugation and lysed by sonication, and subsequently loaded onto a glutathione-Sepharose column (GE Healthcare). The column was washed with PBS and the GST-fusion proteins were eluted using reduced glutathione. The GST tag was cleaved with Precision Protease (GE Healthcare) per the manufacturer's protocol. An additional GST affinity column removed the GST moiety. Su(H) and NICD constructs were further purified to homogeneity using cation exchange and size exclusion chromatography.

ITC experiments were carried out using a MicroCal VP-ITC microcalorimeter. All Su(H)-NICD and Su(H)-DNA experiments were performed at 25°C and 10°C, respectively, in a buffer composed of 50 mM sodium phosphate pH 6.5 and 150 mM NaCl. Su(H) and NICD proteins were degassed and buffer-matched using dialysis and size exclusion chromatography. A typical Su(H)-DNA binding experiment contained 10 μM Su(H) in the cell and 100 μM DNA in the syringe. A typical Su(H)-NICD binding experiment contained 50 μM Su(H) in the syringe and 5 μM NICD in the cell. The data were analyzed using ORIGIN software and fit to a one-site binding model.

Band Assay

In the experiments where the band intensity was quantified, we used ImageJ (Schneider et al., 2012) to draw a Region Of Interest (ROI) around the band and a control ROI in the same chromosome, and measure the average fluorescence intensity. We expressed the band intensity as a ratio of mean fluorescence intensity in the band and the control region.

Enrichment of Marks at the *E(spl)* Locus

To show the enrichment of marks across the *E(spl)* locus, we used ImageJ (Schneider et al., 2012) to draw a rectangular ROI of a fixed length (3 to 5 microns, as indicated in each figure) and variable width (to cover the whole chromosomal width) in each nucleus. We measured the profile of fluorescence intensity across the width of the ROI in the locus tag channel and the corresponding mark. We normalized the values of each nucleus so that they range from 0 to 1 (minimum value=0, maximum value=1) for better comparison between nucleus and experiments. Finally, we aligned all measurements so that the max intensity of the locus tag channel is in the middle of the profile, before averaging all replicates.

Statistical Analyses

The statistical comparisons of different genotypes were done by student t-test or One-Way ANOVA, depending on the number of comparisons. Error bars in all graphs except box and whiskers, represent the standard error of the mean. p-values are indicated as follows: * 0.05-0.01, ** 0.01-0.001, *** 0.001-0.0001, ****<0.0001. The box and whiskers plots show minimum to maximum values. All details of statistical analyses, including n values, are found in the figure legends.

Developmental Cell, Volume *44*

Supplemental Information

Activation of the Notch Signaling Pathway *In Vivo* Elicits Changes in CSL Nuclear Dynamics

Maria J. Gomez-Lamarca, Julia Falo-Sanjuan, Robert Stojnic, Sohaib Abdul Rehman, Leila Muresan, Matthew L. Jones, Zoe Pillidge, Gustavo Cerda-Moya, Zhenyu Yuan, Sarah Baloul, Phillippe Valenti, Kerstin Bystricky, Francois Payre, Kevin O'Holleran, Rhett Kovall, and Sarah J. Bray

Supplementary material

This PDF file includes:

Supplementary Figures S1 to S7 and legends
Tables S1 to S5 and legends

Other Supplementary Materials for this manuscript includes the following:

Movies S1 to S2

Figure S1

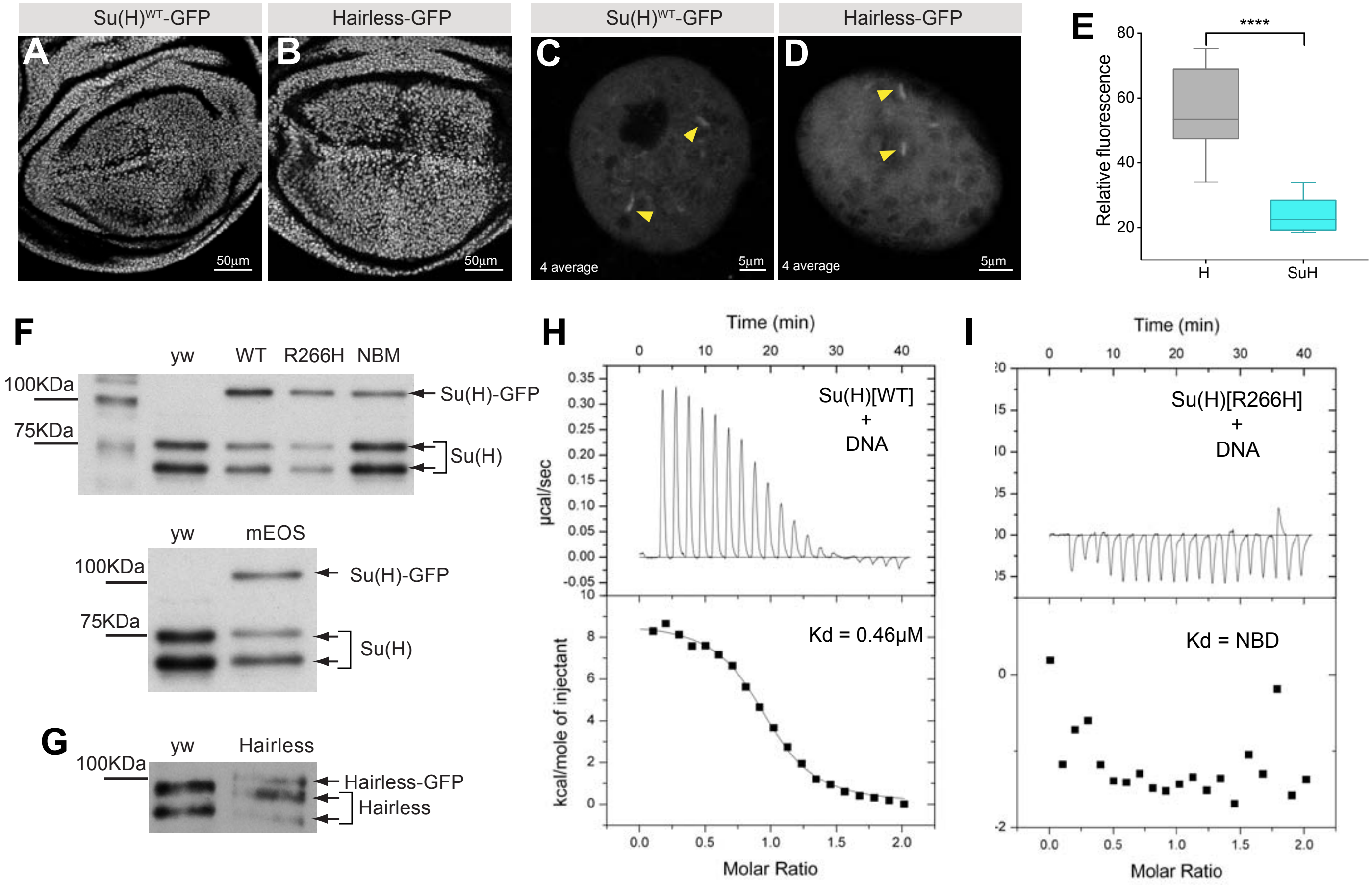

A
B
C
D
E
Su(H)WT-GFP
Hairless-GFP
50µm
5µm
4 average
****
Relative fluorescence
H
SuH
F
yw
WT
R266H
NBM
100KDa
75KDa
Su(H)-GFP
Su(H)
mEOS
G
Hairless
Hairless-GFP
H
I
Time (min)
µcal/sec
kcal/mole of injectant
Molar Ratio
Su(H)[WT]
+
DNA
Kd = 0.46µM
Su(H)[R266H]
+
DNA
Kd = NBD

Figure. S1; Related to Figure 1. Characteristics of Su(H)^{WT}::GFP, Hairless::GFP, Su(H)^{R266H}::GFP

(**A-B**) Wing imaginal disc expressing Su(H)^{WT} (**A**) or Hairless::GFP (**B**). (**C-D**) Salivary gland cells expressing Su(H)^{WT} (**C**) or Hairless::GFP (**D**), imaged with 4 frame averages, note binding at several loci in the polytene chromosomes (arrowheads). (**E**) Fluorescence levels of Su(H)^{WT}::GFP and Hairless::GFP in salivary gland nuclei (n=9, Box and whiskers, Min-to-Max, ****$p < 0.0001$, unpaired two-tailed t test). (**F,G**) Fusion proteins are expressed at similar levels to endogenous. (**F**) Levels of Su(H) detected by western blot in controls (yw) or flies expressing Su(H)^{WT}::GFP, Su(H)^{R266H}::GFP, Su(H)^{NBM}::GFP, Su(H)::mEOS; bands corresponding to endogenous Su(H) or Su(H)::GFP are indicated by arrows. Extracts were prepared from salivary glands of larvae homozygous for the transgene except for Su(H)^{NBM}::GFP which were prepared from heterozygous larva, with only 1 copy of the transgene. (**G**) Levels of Hairless detected in controls (yw) or flies expressing Hairless::GFP, as in F. (**H-I**) Isothermal calorimetry measurements for Su(H)^{WT} (**H**) or Su(H)^{R266H} (**I**) with DNA.

Figure S2

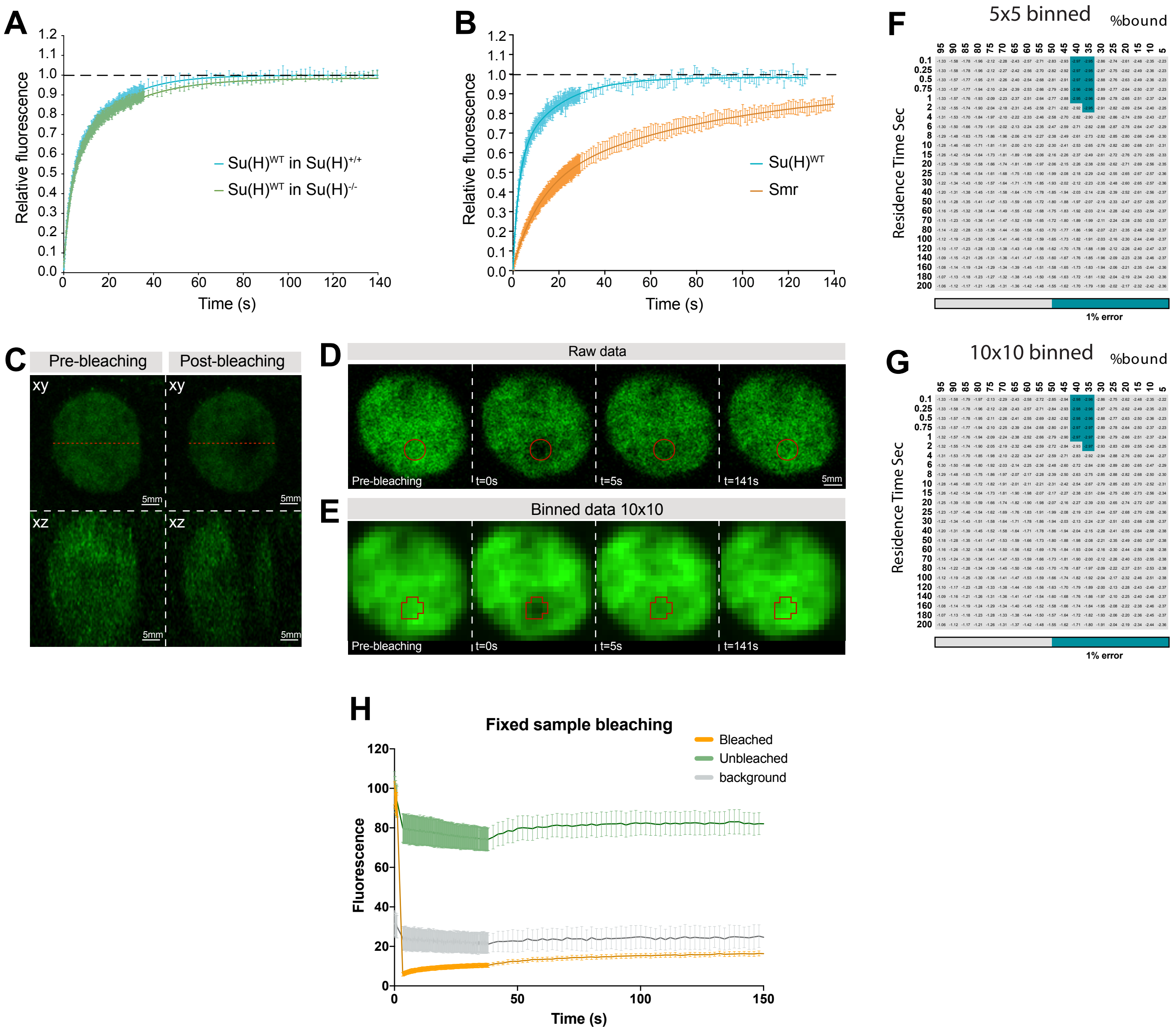
A
Relative fluorescence
Time (s)
Su(H)WT in Su(H)+/+
Su(H)WT in Su(H)-/-
B
Relative fluorescence
Time (s)
Su(H)WT
Smr
C
Pre-bleaching
Post-bleaching
xy
xz
5mm
D
Raw data
Pre-bleaching
t=0s
t=5s
t=141s
5mm
E
Binned data 10x10
Pre-bleaching
t=0s
t=5s
t=141s
F
5x5 binned
%bound
Residence Time Sec
1% error
G
10x10 binned
%bound
Residence Time Sec
1% error
H
Fixed sample bleaching
Bleached
Unbleached
background
Fluorescence
Time (s)

Figure S2; Related to Figure 1. FRAP profiles and extra controls, including SMRTER::YFP FRAP curve

(**A**) Comparison between FRAP curves obtained for $Su(H)^{WT}$-N-eGFP in a WT background or in a transheterozygote flies carrying two Su(H) null alleles, $Su(H)^{SF8}$ and $Su(H)^{A9}$, following point-bleaching at random positions in the nuclei (Mean ± SEM). (**B**) FRAP curves obtained for the indicated proteins, following half nucleus bleaching (Mean ± SEM). (**C**) Bleaching profile following point-bleaching at random positions in the nucleus of a fixed sample. (**D-E**) Example images extracted at the indicated times from FRAP movies showing raw data (**D**) or 10x10 binned data for modelling (**E**). (**F-G**) Comparison of combinations of residence time and percentage of bound molecules giving best-fit to FRAP data, using the same image with 5x5 or 10x10 pixel binning before the modelling. Grey-blue indicates combinations with ≤1% error around the optimal value. (**H**) Whole cell bleaching in a fixed sample shows only a small contribution from reversible photobleaching to the recovery curve, with levels below the background signal (Mean ± SEM).

Figure S3

A

50ms exposure time

	State F1/F2	State F2/B2	State B1a	State B1b
Diff. Coef.	Blurred out 68.5%	0.4µm²/s	0.07µm²/s	0.02µm²/s
% of 10ms loc.		6.3	11	14.2
mean time		111ms	155ms	560ms

B

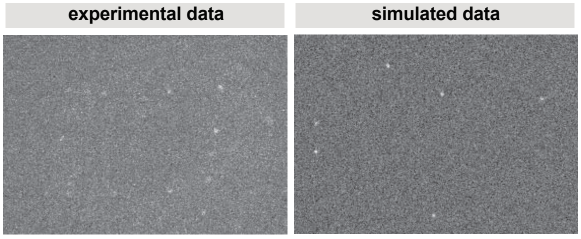

C

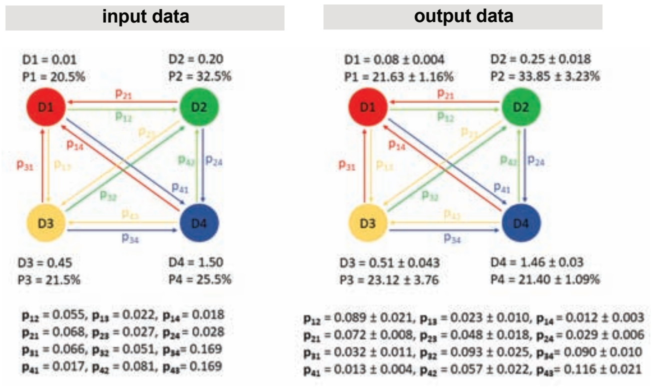

Figure S3; Related to Figure 2. Extra SMT analysis of Su(H)::mEOS.

(**A**) Table of Diffusion coefficients, proportions of molecules belonging to different groups and mean times for each state in Notch^{OFF} conditions, calculated as in (Persson et al., 2013) from 50ms data (no constraints), and adjusted for the proportion of molecules that were blurred out with this exposure setting (See Figure 2A,B) (**B**) Comparison between a real SMT image and a simulated one. (**C**) Diagram of Diffusion coefficients, proportions of molecules belonging to different groups and dwell times for each state in the simulations performed, calculated as in (Persson et al., 2013).

Figure S4

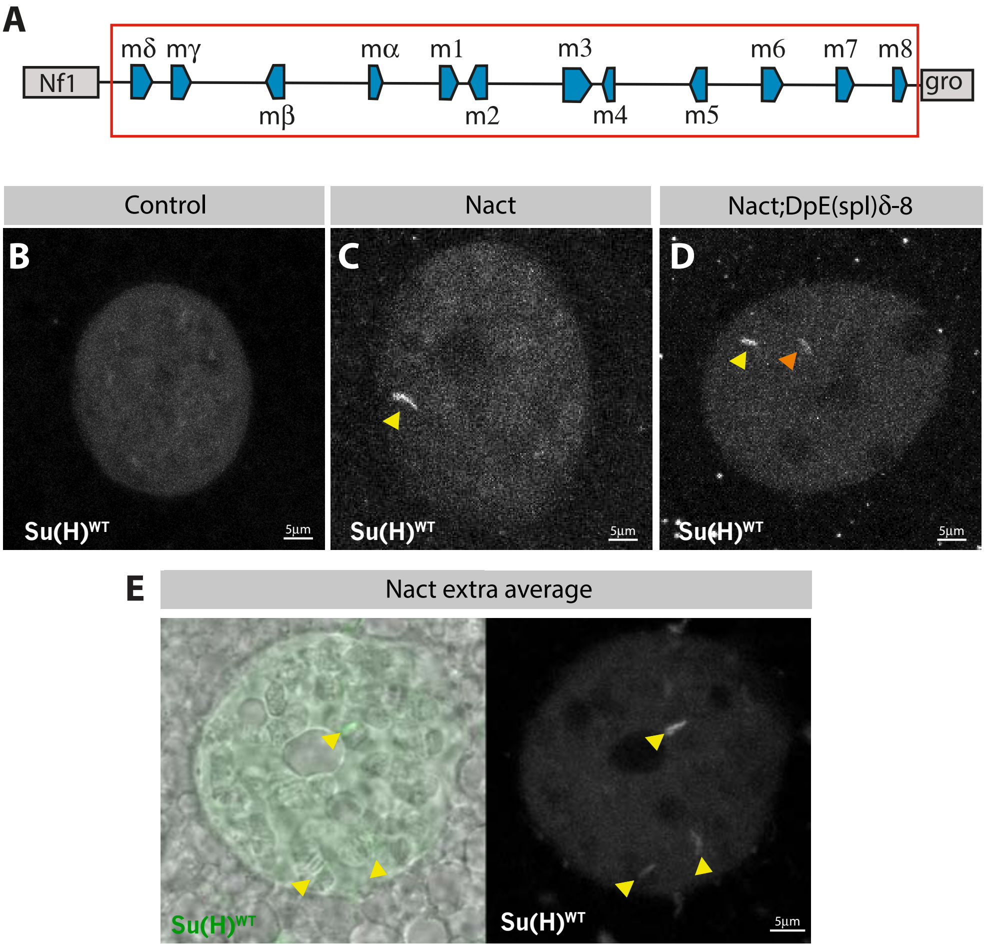

A
Nf1
mδ
mγ
mβ
mα
m1
m2
m3
m4
m5
m6
m7
m8
gro
Control
Nact
Nact;DpE(spl)δ-8
B
C
D
Su(H)WT
5µm
E
Nact extra average

Figure S4; Related to Figure 3. Ectopic site of Su(H):GFP recruitment in nuclei containing duplicated *E(spl)-C*.

(**A**) Schematic representation of the *E(spl)-C* genomic region, indicating the region included in the BAC, inserted in DpE(spl)∂-8. (**B-D**) Live imaging of Su(H)^{WT} in the conditions indicated. When the BAC is present, two sites of Su(H)^{WT}::GFP recruitment are detected in Notch ON cells, at the endogenous *E(spl)-C* (**D**, yellow arrowhead) and at the BAC copy (**D** orange arrowhead), compare to control cells with Notch only (**C**). Note the differing intensity of Su(H)::GFP bands due to only a single copy of the BAC being present. (**E**) Images showing extra SuH(H)::GFP bands observed in Notch ON cells.

Figure S5

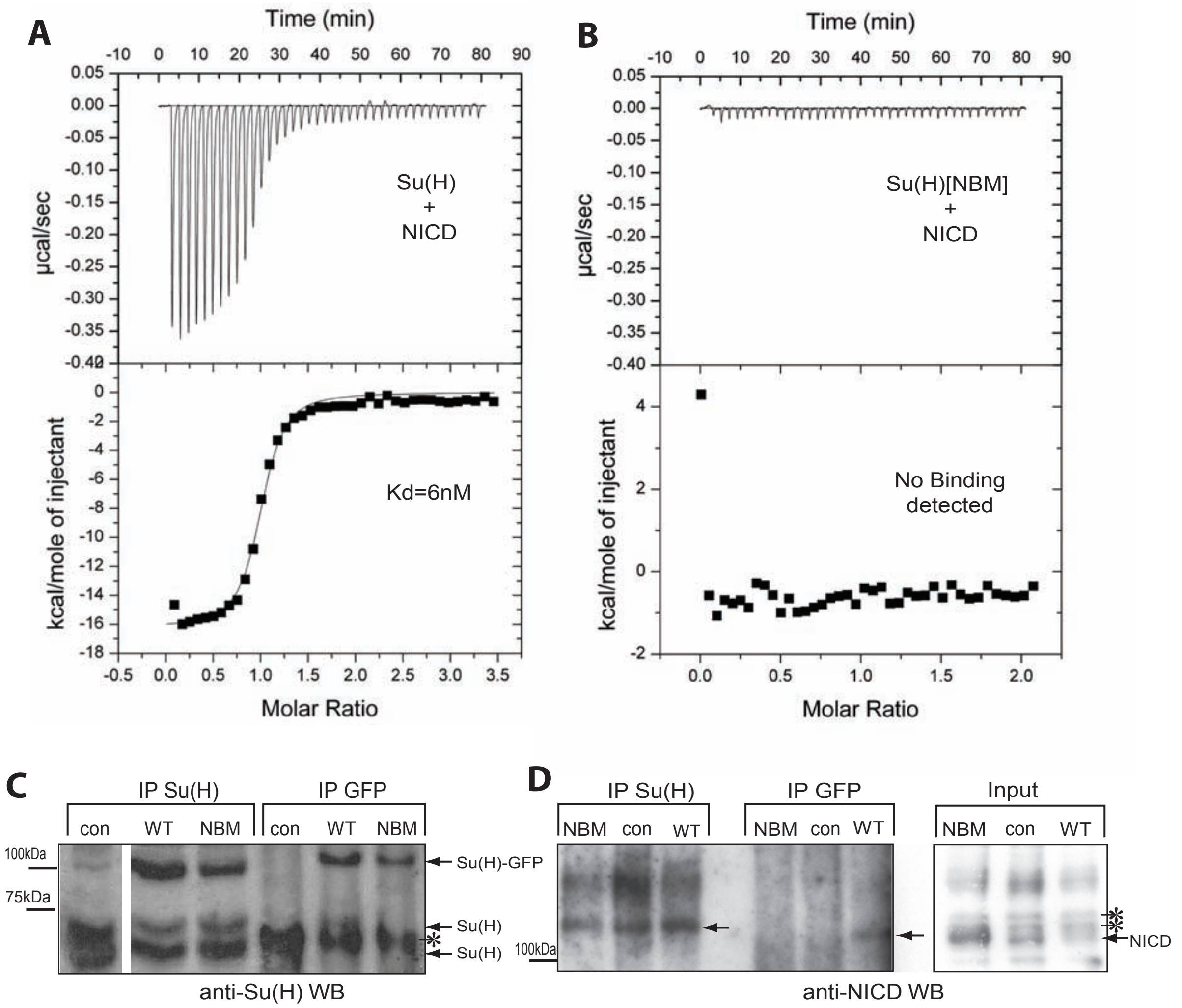

A
Time (min)
µcal/sec
Su(H)
+
NICD
kcal/mole of injectant
Kd=6nM
Molar Ratio
B
Time (min)
µcal/sec
Su(H)[NBM]
+
NICD
kcal/mole of injectant
No Binding
detected
Molar Ratio
C
IP Su(H)
IP GFP
con
WT
NBM
con
WT
NBM
100kDa
75kDa
Su(H)-GFP
Su(H)
*
Su(H)
anti-Su(H) WB
D
IP Su(H)
IP GFP
Input
NBM
con
WT
NBM
con
WT
NBM
con
WT
100kDa
*
NICD
anti-NICD WB

Figure S5; Related to Figure 4. Su(H)^{NBM} mutation blocks binding to NICD

(**A-B**) Isothermal calorimetry measurements of complexes produced between Su(H)^{WT} (**A**) or Su(H)^{NBM} (**B**) and NICD; with Su(H)^{NBM} no interaction is detected. (**C-D**) Co-immunoprecipitation (Co-IP) using anti-Su(H) or anti-GFP antibodies as indicated, and immunoblotted using anti-Su(H) (**C**) or anti-NICD (**D**). In C, positions for endogenous Su(H) or Su(H)::GFP are indicated by arrows, asterisk indicates non-specific band. In (**D**) NICD is indicated by arrows, and is not co-precipitated with Su(H)^{NBM}. Extracts for Co-IPs were prepared from larval heads with salivary glands.

Figure S6

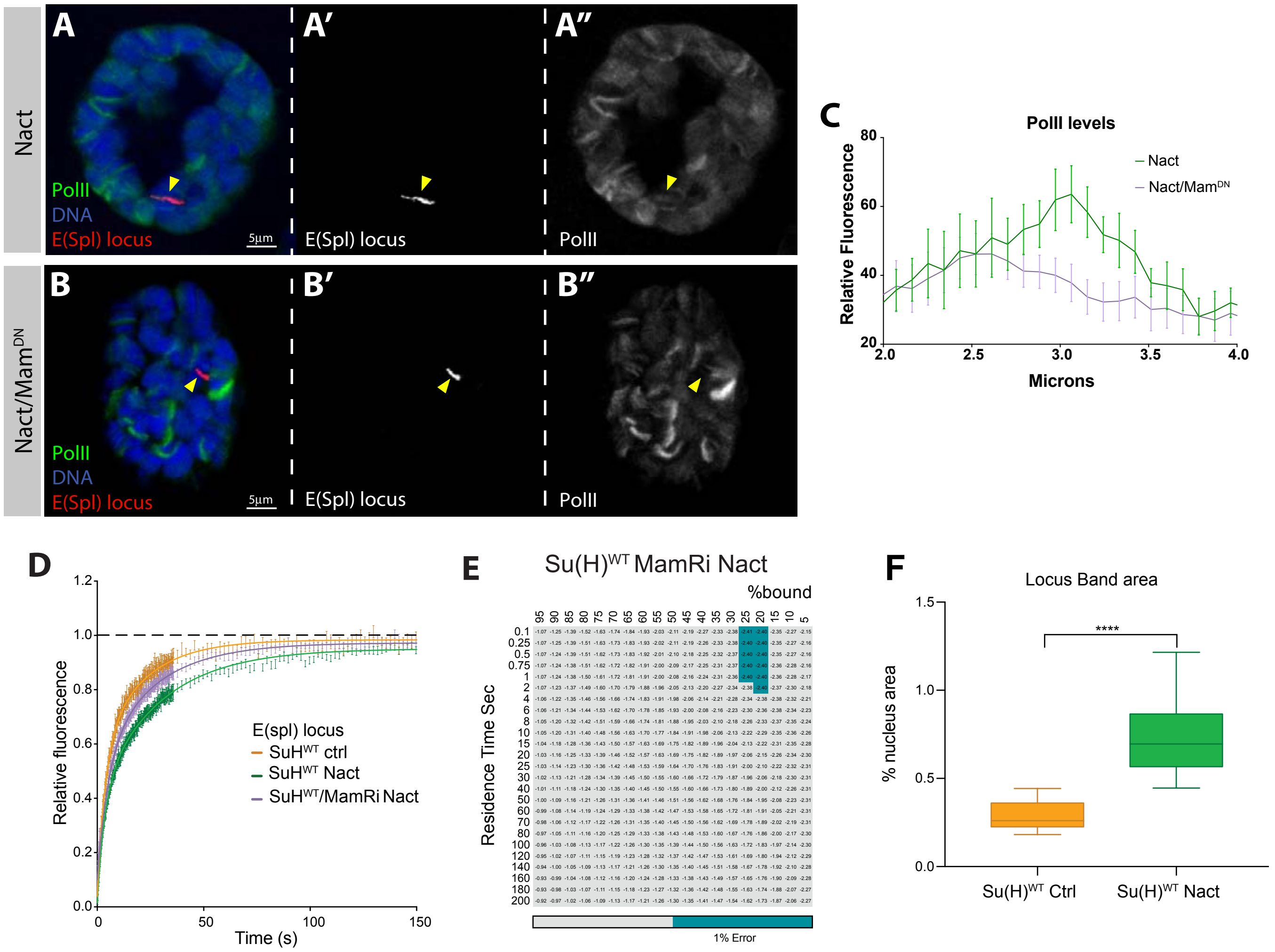

A
A′
A″
Nact
PolII
DNA
E(Spl) locus
5μm
E(Spl) locus
PolII
B
B′
B″
Nact/Mam^DN
PolII
DNA
E(Spl) locus
5μm
E(Spl) locus
PolII
C
PolII levels
Relative Fluorescence
Microns
Nact
Nact/Mam^DN
D
Relative fluorescence
Time (s)
E(spl) locus
SuH^WT ctrl
SuH^WT Nact
SuH^WT/MamRi Nact
E
Su(H)^WT MamRi Nact
%bound
Residence Time Sec
1% Error
F
Locus Band area
% nucleus area
****
Su(H)^WT Ctrl
Su(H)^WT Nact

Figure S6; Related to Figure 4. Effects of Mam depletion

(**A-B**) Co-localization of phosphorylated PolII (Ser10) in salivary gland cells with *E(spl)-C* locus tagged (arrows) in Notch ON cells. MamDN (**B**) impairs recruitment of PolII to *E(spl)-C*. (**C**) Quantification of PolII levels across *E(spl)-C* in indicated genotypes (n=19, Mean ± SEM). (**D**) FRAP curves obtained from focused point bleaching of $Su(H)^{WT}$ specifically at *E(spl)-C* in the conditions indicated (Mean ± SEM). (**E**) Combinations of residence time and percentage of bound molecules giving best-fit to FRAP data, with grey-blue indicating combinations with ≤1% error around the optimal value. (**F**) Quantification of the tagged E(spl) locus area, calculated as percentage of total nucleus area (n=11, Box and whiskers Min-to-Max, ****p < 0.0001, unpaired two-tailed t test).

Figure S7

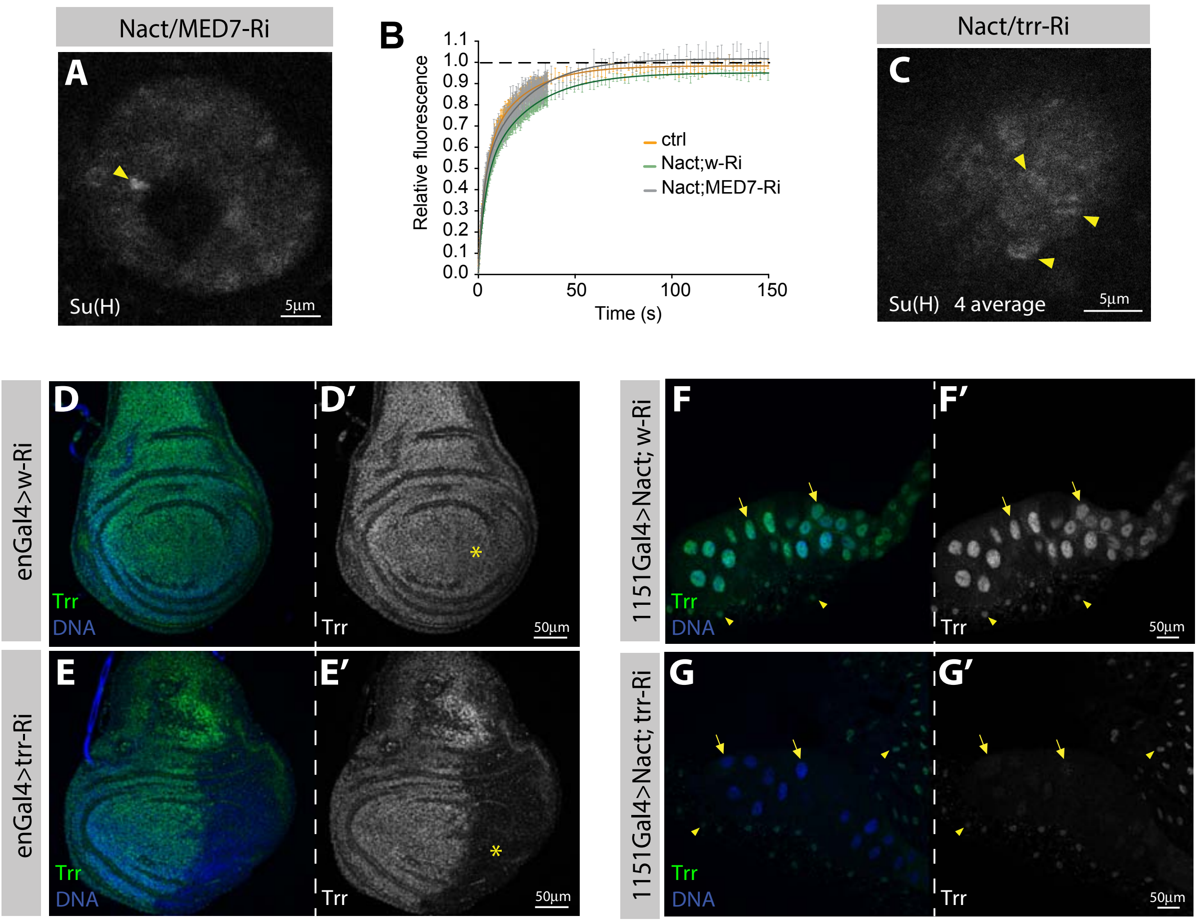

Nact/MED7-Ri
A
Su(H)
5µm
B
Relative fluorescence
1.1
1.0
0.9
0.8
0.7
0.6
0.5
0.4
0.3
0.2
0.1
0.0
0
50
100
150
Time (s)
ctrl
Nact;w-Ri
Nact;MED7-Ri
Nact/trr-Ri
C
Su(H) 4 average
5µm
enGal4>w-Ri
D
Trr
DNA
D′
*
Trr
50µm
enGal4>trr-Ri
E
Trr
DNA
E′
*
Trr
50µm
1151Gal4>Nact; w-Ri
F
Trr
DNA
F′
Trr
50µm
1151Gal4>Nact; trr-Ri
G
Trr
DNA
G′
Trr
50µm

Figure S7; Related to Figure 6. Effects of MED7 depletion and validation of trr-Ri

(**A**) Live imaging of Su(H)^{WT} in the conditions indicated. *MED7* knock-down does not affect Su(H)^{WT}::GFP recruitment in Notch ON cells. (**B**) Comparison between FRAP curves obtained for Su(H)^{WT}::GFP following point-bleaching at the *E(spl)-C* region in the genotypes indicated (Mean ± SEM). Note the faster recovery in the *MED7* KD, compared to the control Nact. (**C**) Live imaging of Su(H)^{WT} with extra averaging, in the conditions indicated, showed that there Su(H)::GFP is still recruited to chromosomes in *trr* KD. (**D-G**) Levels of Trr protein in the genotypes indicated (green in D-G, white in D'G'). *trr* KD reduces the levels of Trr in the wing disc when expressed in the posterior compartment (asterisk, **E**), compared to a WT disc (asterisk, **D**). Also in salivary glands, the expression of a *trr-Ri* reduces the levels of Trr in the polytene cells (arrows, **F-G**) but not in the surrounding fat body (arrowhead).

Supplementary Tables

Table S1; Related to Figure 2. Summary of SMT data analysis for Notch-OFF cells showing four diffusive states, their diffusion coefficients (D), dwell times, proportions and transition probabilities, computed using variational Bayesian treatment of Hidden Markov models based on (Persson et al., 2013).

	State B1	State B2	State F2	State F1	
**D[um^2/s]**					
E1	0.095611	0.25718	0.52097	1.8928	n=8180
E2	0.087384	0.2222	0.46726	1.8028	n=7792
E3	0.089451	0.20991	0.47016	1.8459	n=14064
E4	0.0787	0.2078	0.5302	2.0207	n=7340
**MEAN:**	**0.0877865**	**0.2242725**	**0.4971475**	**1.89055**	
**Proportion of molecules**					
E1	0.25784	0.34973	0.15989	0.23254	
E2	0.23394	0.33737	0.20399	0.2247	
E3	0.1536	0.29858	0.29634	0.25148	
E4	0.2138	0.3548	0.261	0.1704	
**MEAN:**	**0.214795**	**0.33512**	**0.230305**	**0.21978**	
**Av Time (s)**					
E1	0.085075	0.064843	0.036804	0.035313	
E2	0.10514	0.081294	0.034961	0.037498	
E3	0.07615	0.07574	0.046844	0.041131	
E4	0.105	0.088	0.041	0.033	
**MEAN:**	**0.09284125**	**0.07746925**	**0.03990225**	**0.0367355**	
**Transition probability per time step:**					
	**State B1**	**State B2**	**State F2**	**State F1**	
E1	0.88241	0.079774	0.020565	0.017251	*from B1*
	0.11601	0.84567	0.0016371	0.036683	*from B2*
	0.0054658	0.053628	0.7275	0.21341	*from F2*
	0.031774	0.11672	0.1353	0.71621	*from F1*
E2	0.9049	0.054931	0.022124	0.01804	*from B1*
	0.068136	0.87694	0.026886	0.028038	*from B2*
	0.066241	0.051301	0.71329	0.16917	*from F2*
	0.017287	0.081167	0.16881	0.73274	*from F1*
E3	0.86859	0.10375	0.0091448	0.018513	*from B1*
	0.071504	0.86792	0.046039	0.014535	*from B2*

	0.03207	0.065551	0.78635	0.11603	*from F2*
	0.018829	0.026434	0.19813	0.7566	*from F1*
E4	0.9053	0.0615	0.0328	0.0004	*from B1*
	0.0712	0.8868	0.012	0.03	*from B2*
	0.022	0.0964	0.7562	0.1254	*from F2*
	0.0277	0.0339	0.2329	0.7055	*from F1*
**Mean**	0.8903	0.07498875	0.02115845	0.013551	*from B1*
	0.0817125	0.8693325	0.021640525	0.027314	*from B2*
	0.0314442	0.06672	0.745835	0.1560025	*from F2*
	0.0238975	0.06455525	0.183785	0.7277625	*from F1*

Table S2; Related to Figure 2. Summary of SMT data analysis for Notch-OFF cells showing three diffusive states, their diffusion coefficients (D), dwell times, proportions and transition probabilities, computed using variational Bayesian treatment of Hidden Markov models based on (Persson et al., 2013).

	State B1/B2	State F2	State F1	
**D[um^2/s]**				
E1	0.10691	0.32626	1.7732	
E2	0.097838	0.30269	1.6751	
E3	0.12204	0.37456	1.765	
E4	0.098181	0.3352	1.7715	
**MEAN:**	**0.10624225**	**0.3346775**	**1.7462**	
**Proportion of molecules**				
E1	0.32154	0.41432	0.26414	
E2	0.30513	0.43579	0.25909	
E3	0.30707	0.41768	0.27525	
E4	0.33695	0.44183	0.22122	
**MEAN:**	**0.3176725**	**0.427405**	**0.254925**	
**Av Time (s)**				
E1	0.106	0.048726	0.04342	
E2	0.106	0.048726	0.04342	
E3	0.13349	0.05639	0.048199	
E4	0.12925	0.053746	0.044759	
**MEAN:**	**0.118685**	**0.051897**	**0.0449495**	
**Transition probability per time step:**				
	**State B1/B2**	**State F2**	**State F1**	
E1	0.90567	0.081629	0.012699	*from B1/B2*
	0.10964	0.79459	0.095769	*from F2*
	0.024477	0.20621	0.76931	*from F1*
E2	0.90921	0.07733	0.013456	*from B1/B2*
	0.10044	0.81258	0.086985	*from F2*
	0.019252	0.19342	0.78733	*from F1*
E3	0.92512	0.064868	0.01011	*from B1/B2*
	0.093392	0.82258	0.084028	*from F2*
	0.020624	0.18703	0.79234	*from F1*

E4	0.92267	0.075379	0.0019507	*from B1/B2*
	0.099438	0.81381	0.086752	*from F2*
	0.016253	0.20757	0.77618	*from F1*
**Mean**	0.9156675	0.0748015	0.009553925	*from B1/B2*
	0.1007275	0.81089	0.0883835	*from F2*
	0.0201515	0.1985575	0.78129	*from F1*

Table S3; Related to Figure 3. Summary of SMT data analysis for Notch-ON cells showing three diffusive states, their diffusion coefficients (D), dwell times, proportions and transition probabilities, computed using variational Bayesian treatment of Hidden Markov models based on (Persson et al., 2013).

	**State B1/B2**	**State F2**	**State F1**	
**D[um^2/s]**				
E1	0.1303	0.3923	1.5507	
E2	0.1291	0.3752	1.7563	
E3	0.1159	0.3379	1.7095	
E4	0.1365	0.3791	1.8279	
**MEAN:**	**0.12795**	**0.371125**	**1.7111**	
**Proportion of molecules**				
E1	0.3871	0.4328	0.18	
E2	0.3284	0.4008	0.2708	
E3	0.2604	0.4043	0.3352	
E4	0.2579	0.446	0.2961	
**MEAN:**	**0.30845**	**0.420975**	**0.270525**	
**Av Time (s)**				
E1	0.1493	0.0457	0.0327	
E2	0.1193	0.0452	0.0391	
E3	0.118	0.0479	0.0434	
E4	0.1063	0.0514	0.0387	
**MEAN:**	**0.123225**	**0.04755**	**0.038475**	
**Transition probability per time step:**				
	**State B1/B2**	**State F2**	**State F1**	
E1	0.933	0.0566	0.0103	*from B1/B2*
	0.115	0.7811	0.104	*from F2*
	0.0344	0.2717	0.6939	*from F1*
E2	0.9162	0.0628	0.021	*from B1/B2*
	0.1076	0.7788	0.1137	*from F2*
	0.0332	0.2223	0.7445	*from F1*
E3	0.9152	0.0637	0.0211	*from B1/B2*
	0.0802	0.7912	0.1285	*from F2*
	0.0256	0.2048	0.7696	*from F1*

E4	0.9059	0.084	0.01	*from B1/B2*
	0.0719	0.8055	0.1226	*from F2*
	0.0373	0.2209	0.7418	*from F1*
**Mean**	0.917575	0.066775	0.0156	*from B1/B2*
	0.093675	0.78915	0.1172	*from F2*
	0.032625	0.229925	0.73745	*from F1*

Table S4; Related to all figures. Table summarizing genotypes of flies used in each figure.

**Fig1**
Su(H)^{WT}-N-eGFP
Hairless^{WT}-C-eGFP
NRE-GFP
Fkh-GFP
Su(H)^{R266H}-N-GFP
1151-Gal4;;UAS-nls-GFP
Su(H)^{SF8};Su(H)^{WT}-N-eGFP x Su(H)^{A9};Su(H)^{WT}-N-eGFP

**Fig2**
Su(H)^{WT}-N-mEOS3.2

**Fig3**
1151-Gal4;; Su(H)^{WT}-N-GFP
1151-Gal4;; Su(H)^{WT}-N-GFP x UAS-N$^{\Delta ECD}$
1151-Gal4;; Su(H)^{R266H}-N-GFP x UAS-N$^{\Delta ECD}$
1151-Gal4;; Su(H)^{WT}-N-GFP,p31A-mCherry x m8intA
1151-Gal4;; Su(H)^{WT}-N-GFP,p31A-mCherry x UAS-N$^{\Delta ECD}$; m8intA
1151-Gal4;; Su(H)^{WT}-N-mEOS x UAS-N$^{\Delta ECD}$;Su(H)^{WT}-N-mEOS

**Fig 4**
1151-Gal4;; Su(H)^{WT}-N-GFP, p31A-mCherry x m8intA
1151-Gal4;; Su(H)^{WT}-N-GFP,p31A-mCherry x UAS-N$^{\Delta ECD}$; m8intA
1151-Gal4;; Su(H)^{WT}-N-mEOS,p31A-GFP x Su(H)^{WT}-N-mEOS,m8intA
1151-Gal4;; Su(H)^{WT}-N-mEOS,p31A-GFP x UAS-N$^{\Delta ECD}$; Su(H)^{WT}-N-mEOS,m8intA

**Fig5**
1151-Gal4;; Su(H)^{WT}-N-GFP x UAS-N$^{\Delta ECD}$
1151-Gal4;; Su(H)^{NBM}N-GFP x UAS-N$^{\Delta ECD}$
1151-Gal4;UAS-Mam^{DN} x UAS-N$^{\Delta ECD}$; Su(H)^{WT}-N-GFP
1151-Gal4;; Su(H)^{WT}-N-GFP x UAS-N$^{\Delta ECD}$;UAS-H-Ri
1151-Gal4;; Su(H)^{WT}-N-GFP x UAS-H-Ri
1151-Gal4;; Su(H)^{NBM}-N-GFP x UAS-N$^{\Delta ECD}$;UAS-H-Ri
1151-Gal4;; Su(H)^{WT},p31A-mCherry x m8intA
1151-Gal4;; Su(H)^{WT},p31A-mCherry x UAS-N$^{\Delta ECD}$; m8intA

**Fig6**
1151-Gal4;;p31A-mCherry x m8intA
1151-Gal4;;p31A-mCherry x UAS-N$^{\Delta ECD}$; m8intA

1151-Gal4;; Su(H)^{WT}-N-GFP x UAS-LacZ
1151-Gal4;; Su(H)^{WT}-N-GFP x UAS-N$^{\Delta ECD}$
1151-Gal4; Hairless-C-GFP x UAS-p31A-mCherry, m8intA
1151-Gal4; Hairless-C-GFP x UAS-N$^{\Delta ECD}$;UAS-p31A-mCherry, m8intA

**Fig7**
1151-Gal4;; Su(H)^{WT}-N-GFP x UAS-N$^{\Delta ECD}$
1151-Gal4;; Su(H)^{WT}-N-GFP x UAS-E(z)-RNAi
1151-Gal4;; Su(H)^{WT}-N-GFP x UAS-N$^{\Delta ECD}$;UAS-w-Ri
1151-Gal4;; Su(H)^{WT}-N-GFP x UAS-N$^{\Delta ECD}$;UAS-Trr-Ri
1151-Gal4;UAS-Mam^{DN} x UAS-N$^{\Delta ECD}$; Su(H)^{WT}-N-GFP

**SupFig1**
yw
Su(H)^{WT}-N-eGFP
Hairless^{WT}-C-eGFP
Su(H)^{R266H}-N-eGFP
Su(H)^{WT}-N-mEOS3.2

**SupFig2**
Su(H)^{WT}-N-eGFP
Su(H)^{SF8};Su(H)^{WT}-N-eGFP x Su(H)^{A9};Su(H)^{WT}-N-eGFP
Smr-YFP
Hairless-C-GFP

**SupFig3**
Su(H)^{WT}-N-mEOS3.2

**SupFig4**
1151-Gal4;;Su(H)^{WT}-N-GFP
1151-Gal4;; Su(H)^{WT}-N-GFP x UAS-N$^{\Delta ECD}$
1151-Gal4;DpE(Spl)mδ-m8 x UAS-N$^{\Delta ECD}$;Su(H)^{WT}-N-GFP

**SupFig5**
yw
Su(H)^{WT}-N-GFP
Su(H)^{NBM}-N-GFP

**SupFig6**
1151-Gal4 x UAS-N$^{\Delta ECD}$;p31A-mcherry, m8intA
1151-Gal4;UAS-Mam^{DN} x UAS-N$^{\Delta ECD}$; p31A-mCherry, m8intA

1151-Gal4;; $Su(H)^{WT}$,p31A-mCherry x m8intA
1151-Gal4;; $Su(H)^{WT}$,p31A-mCherry x UAS-$N^{\Delta ECD}$; m8intA
1151-Gal4;UAS-Mam^{DN} x UAS-$N^{\Delta ECD}$; $Su(H)^{WT}$-N-GFP

**SupFig7**
1151-Gal4;; $Su(H)^{WT}$ x UAS-$N^{\Delta ECD}$; MED7-Ri
1151-Gal4;; $Su(H)^{WT}$ x UAS-$N^{\Delta ECD}$; trr-Ri
1151-Gal4;; $Su(H)^{WT}$ x UAS-$N^{\Delta ECD}$; w-Ri
en-Gal4, Gal80ts;w-Ri
en-Gal4, Gal80ts;w-Ri x trr-Ri

Table S5; Related to STAR Methods. List of oligonucleotides.

Name	Sequence	Purpose
E(spl)-mα forward	GCAGGAGGACGAGGAGGATG	mRNA levels
E(spl)-mα reverse	GATCCTGGAATTGCATGGAG	mRNA levels
E(spl)-mβ forward	GCTGGACTTGAAACCGC	mRNA levels
E(spl)-mβ reverse	AGAAGTGAGCAGCAGCC	mRNA levels
E(spl)-m3 forward	AGCCCACCCACCTCAAC	mRNA levels
E(spl)-m3 reverse	GTCTGCAGCTCCATTAGTC	mRNA levels
Rpl32 forward	ATGCTAAGCTGTCGCACAAATG	mRNA levels
Rpl32 reverse	GTTCGATCCGTAACCGATGT	mRNA levels
Nextera PCR primer 1	AATGATACGGCGACCACCGAGATCTACACTCGTCGG CAGCGTCAGATGTG	ATAC
Nextera PCR primer 2.1	CAAGCAGAAGACGGCATACGAGATTCGCCTTAGTCT CGTGGGCTCGGAGA	ATAC
Nextera PCR primer 2.2	CAAGCAGAAGACGGCATACGAGATCTAGTACGGTCT CGTGGGCTCGGAG	ATAC
Nextera PCR primer 2.3	CAAGCAGAAGACGGCATACGAGATTTCTGCCTGTCT CGTGGGCTCGGAGA	ATAC
Nextera PCR primer 2.4	CAAGCAGAAGACGGCATACGAGATGCTCAGGAGTCT CGTGGGCTCGGAG	ATAC
Nextera PCR primer 2.5	CAAGCAGAAGACGGCATACGAGATAGGAGTCCGTCT CGTGGGCTCGGAGATGT	ATAC
Nextera PCR primer 2.6	CAAGCAGAAGACGGCATACGAGATCATGCCTAGTCT CGTGGGCTCGGAGA	ATAC
Nextera PCR primer 2.7	CAAGCAGAAGACGGCATACGAGATGTAGAGAGGTCT CGTGGGCTCGGAGATGT	ATAC
Nextera PCR primer 2.8	CAAGCAGAAGACGGCATACGAGATCCTCTCTGGTCT CGTGGGCTCGGAGA	ATAC
Nextera PCR primer 2.9	CAAGCAGAAGACGGCATACGAGATAGCGTAGCGTCT CGTGGGCTCGGAG	ATAC
Nextera PCR primer 2.10	CAAGCAGAAGACGGCATACGAGATCAGCCTCGGTCT CGTGGGCTCGGAGATGT	ATAC
Nextera PCR primer 2.11	CAAGCAGAAGACGGCATACGAGATTGCCTCTTGTCT CGTGGGCTCGGAGATGT	ATAC
Nextera PCR primer 2.12	CAAGCAGAAGACGGCATACGAGATTCCTCTACGTCT CGTGGGCTCGGAGATGT	ATAC
Nextera PCR primer 2.13	CAAGCAGAAGACGGCATACGAGATATCACGACGTCT CGTGGGCTCGGAGATGT	ATAC
Nextera PCR primer 2.14	CAAGCAGAAGACGGCATACGAGATACAGTGGTGTCT CGTGGGCTCGGAGATGT	ATAC
Nextera PCR primer 2.15	CAAGCAGAAGACGGCATACGAGATCAGATCCAGTCT CGTGGGCTCGGAGATGT	ATAC
Rab11 intron forward	ACTGAAAATGGGCCGTTTCG	ATAC
Rab11 intron reverse	AGGAGTGGTAATCGACGGTC	ATAC
Eip78C EcR forward	AGAAGTAGGGGCCGTCAAGT	ATAC

Eip78C EcR reverse	GTGTAAGACCCGTCGCATTT	ATAC
Negative 1 forward	GCATTTTTGTGGCAGAGGCA	ATAC
Negative 1 reverse	CTCTTTCGGTGTCGCCTTCT	ATAC
Mst87F forward	ATCCTTTGCCTCTTCAGTCC	ATAC
Mst87F reverse	AATAATGATACAAAATCTGGTTACGC	ATAC
mβ gene forward	AGAAGTGAGCAGCAGCCATC	ATAC
mβ gene reverse	GCTGGACTTGAAACCGCACC	ATAC
mβ peak forward	AGAGGTCTGTGCGACTTGG	ATAC
mβ peak reverse	GGATGGAAGGCATGTGCT	ATAC
mα peak forward	AAGCCAGTGGACTCTGCTCT	ATAC
mα peak reverse	TGATCTCCAAGCGGAGTATG	ATAC
mα gene forward	GCAGGAGGACGAGGAGGATG	ATAC
mα gene reverse	GATCCTGGAATTGCATGGAG	ATAC
m3 peak forward	ACACACACAAACACCCATCC	ATAC
m3 peak reverse	CGAGGCAGTAGCCTATGTGA	ATAC
m3 gene forward	CGTCTGCAGCTCAATTAGTC	ATAC
m3 gene reverse	AGCCCACCCACCTCAACCAG	ATAC
m8 gene forward	CAATTCCACGAAGCACAGTC	ATAC
m8 gene reverse	GAGGAGCAGTCCATCGAGTT	ATAC
